# Association between gestational weight gain and adverse pregnancy outcomes: cohort analysis from South Asia and Sub-Saharan Africa

**DOI:** 10.1136/bmjph-2024-000900

**Published:** 2025-02-04

**Authors:** Yuri V Sebastião, Ramachandran Thiruvengadam, Rasheda Khanam, Usma Mehmood, Jesmin Pervin, Ayushi, Bapu Koundinya Desiraju, Fatma Kabole, Salahuddin Ahmed, Shaki Aktar, Nabidul Haque Chowdhury, Muhammad Farrukh Qazi, Imran Nisar, Javairia Khalid, Margaret Kasaro, Bellington Vwalika, Waqasuddin Khan, U Tin Nu, Monjur Rahman, Sayedur Rahman, Gary M Shaw, David K Stevenson, Huan Xu, Bihila Abdalla Bakari, Nitya Wadhwa, Ge Zhang, Sunil Sazawal, Nima Aghaeepour, Anisur Rahman, Fyezah Jehan, Abdullah H Baqui, Jeffrey S A Stringer, Shinjini Bhatnagar

**Affiliations:** 1 Division of Global Women's Health, Department of Obstetrics and Gynecology, The University of North Carolina at Chapel Hill, Chapel Hill, North Carolina, USA; 2 Maternal and Child Health Program, Translational Health Science and Technology Institute, Faridabad, Haryana, India; 3 Department of Biochemistry, Pondicherry Institute of Medical Sciences, Pondicherry, India; 4 Department of International Health, Johns Hopkins Bloomberg School of Public Health, Baltimore, Maryland, USA; 5 Department of Pediatrics and Child Health, Medical College, Aga Khan University, Karachi, Pakistan; 6 Maternal and Child Health Division, International Centre for Diarrhoeal Disease Research Bangladesh, Dhaka, Bangladesh; 7 Ministry of Health, Zanzibar, Tanzania, United Republic of; 8 Projahnmo Research Foundation, Dhaka, Bangladesh; 9 Center for Public Health Kinetics, Zanzibar, Tanzania, United Republic of; 10 Department of Obstetrics and Gynaecology, University of Zambia School of Medicine, Lusaka, Zambia; 11 Department of Women's and Children's Health, Uppsala University, Uppsala, Sweden; 12 School of Medicine, Stanford University, Stanford, California, USA; 13 Division of Human Genetics, Cincinnati Children's Hospital Medical Center, Cincinnati, Ohio, USA; 14 Swami Vivekanand Subharti University, Meerut, Uttar Pradesh, India

**Keywords:** Epidemiology, Body Mass Index, Effect Modifier, Epidemiologic

## Abstract

**Introduction:**

Studies of gestational weight gain (GWG) and adverse pregnancy outcomes seldom focus on low-to-middle-income countries (LMICs), despite their high burden of morbidity and mortality. We examined GWG patterns and adverse pregnancy outcomes in a consortium of pregnancy cohorts from LMICs.

**Methods:**

We analysed data from five observational pregnancy cohorts in Bangladesh (two cohorts), India, Pakistan and Zambia. The study population comprised 15 286 singleton pregnancies with two or more maternal antenatal weight measurements. We estimated reference values for GWG using longitudinal models and calculated weight gain for gestational age Z-scores. We then estimated the associated risks of preterm birth, low birth weight, and small for gestational age, stratified by maternal body mass index (BMI), using marginal generalised linear models and plotted non-linear trends in the associations.

**Results:**

The median baseline maternal and gestational age were 24 years (IQR, 21–28) and 13 weeks (IQR 11–16), respectively, with 23% of participants having underweight BMI. The median GWG was 6.8 kg (4.2–9.4) and varied across cohorts from 6.1 kg (3.7–8.5; Bangladesh) to 7.0 kg (4.0–10.0; Zambia). The risk of preterm birth (13%) increased with lower GWG Z-scores among underweight (adjusted risk ratio (ARR), 1.4; 95% CI, 1.1 to 1.9 for lowest Z-score group) and normal BMI participants (ARR, 1.1; 95% CI, 1.0 to 1.2). The risk of low birth weight (25%) increased with lower GWG Z-scores in all BMI strata except obese participants (ARR, 1.7; 95% CI 1.5 to 1.9 among underweight). The risk of small for gestational age (36%) increased with lower GWG Z-scores in all BMI strata (ARR, 1.3; 95% CI 1.2 to 1.4 among underweight). In secondary analyses, alternative measures of GWG (adequacy ratio; INTERGROWTH-21^st^) had associations that were consistent with those from our study-specific Z-scores, except for a less clear association between preterm birth and INTERGROWTH-21^st^ Z-score.

**Conclusion:**

GWG was associated with preterm birth, low birth weight and small for gestational age. Early pregnancy BMI modified the association between GWG and outcomes in the study setting.

**WHAT IS ALREADY KNOWN ON THIS TOPIC**

**WHAT THIS STUDY ADDS**

- Using data from 15 286 pregnancies in Bangladesh, India, Pakistan and Zambia, we estimate the association of gestational weight gain and adverse pregnancy outcomes using weight gain for gestational age standards from the study population.
- Gestational weight gain was associated with preterm birth, low birth weight and small for gestational age, and these associations varied by baseline maternal body mass index (BMI).

**HOW THIS MIGHT AFFECT RESEARCH, PRACTICE OR POLICY**

- Our findings highlight the role that both early pregnancy maternal BMI and gestational weight gain may play in the prevention of adverse pregnancy outcomes in low-to-middle-income country settings.

## Introduction

 Pregnancy outcomes continue to be suboptimal in low-to-middle-income countries (LMICs). In combination, the two regions of South Asia and Sub-Saharan Africa account for over 60% of worldwide preterm birth prevalence and over 70% of worldwide under 5 mortality due to preterm birth.^1 2^ A similarly high burden of both incidence and mortality related to low birth weight and small for gestational age is known to occur in South Asia and Sub-Saharan Africa.^3^,^5^

Gestational weight gain (GWG) is recognised as a risk factor for preterm birth that is potentially amenable to intervention.^6^ Several observational studies in high-income countries have reported an association between inadequate GWG and outcomes such as preterm birth, small for gestational age as well as an association between excessive weight gain and outcomes such as large for gestational age.^7^ Despite growing consensus about the importance of GWG, associations with adverse pregnancy outcomes are seldom quantified in LMICs.^8^

Assessment of GWG is inherently challenging in LMIC settings for the lack of standards and guidelines applicable for these settings. The Institute of Medicine (IOM) 2009 guidelines are intended for use in high-income settings and have not been calibrated for LMICs.^6^ On the other hand, the INTERGROWTH-21st (IG-21) standards that have representation from LMICs have been constructed only for women who start their pregnancy with a normal body mass index (BMI), thus limiting its applicability in settings where underweight women contribute to a high proportion women seeking antenatal care.^9^

In this study, we assessed the patterns of GWG and estimated the associations between GWG and adverse pregnancy outcomes in a consortium of five pregnancy cohorts from LMICs.

## Methods

### Study design and population

**Figure 1:**
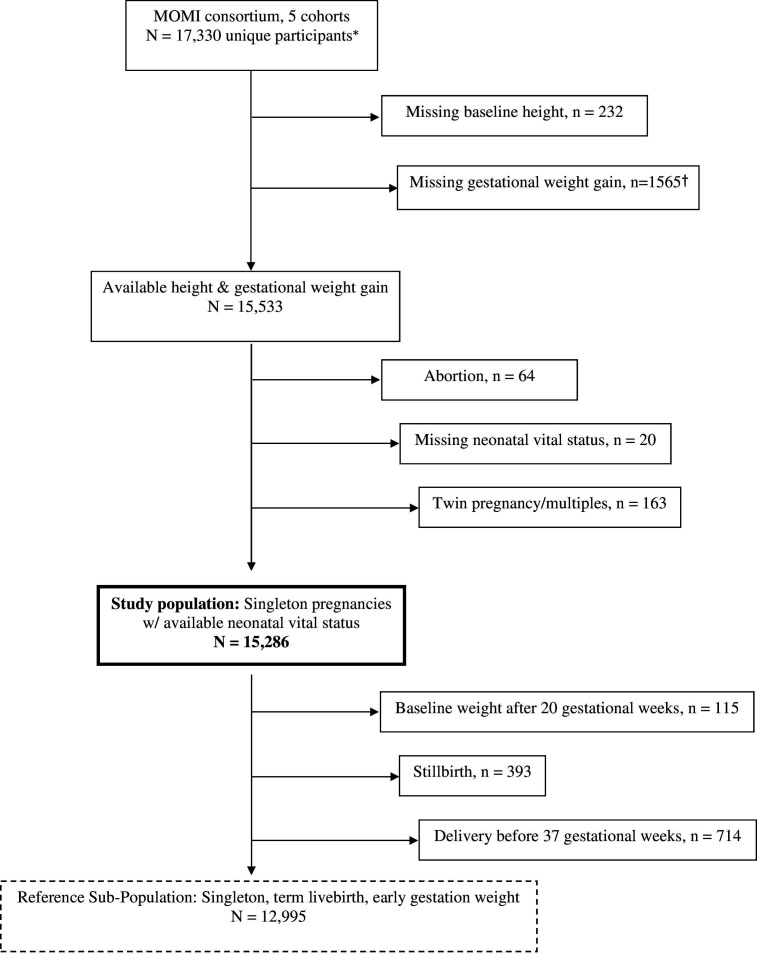
Study cohort selection flowchart. *First available pregnancy: delivery ≤42 weeks; †2+ antenatal visits with maternal weight measurements.

We conducted a retrospective cohort analysis of data from the Multi-Omics for Mothers and Infants Consortium (MOMI). The consortium comprises six longitudinal pregnancy cohorts in five countries: three cohorts from the Alliance for Maternal and Neonatal Health Improvement (AMANHI) in Sylhet (Bangladesh), Karachi (Pakistan) and Pemba (Tanzania); two cohorts from the Global Alliance to Prevent Prematurity and Stillbirth in MATLAB (Bangladesh, Preterm and Stillbirth Study (PreSSMat)) and Lusaka (Zambian Preterm Birth Prevention Study, ZAPPS); and one cohort from the interdisciplinary Group for Advanced Research on Birth outcomes – DBT India Initiative in Gurugram, Haryana (GARBH-Ini).^10 11^ Country-level human development index (HDI), infant (IMR) and maternal mortality (MMR) for the study locations are provided in the online supplemental file 1. The 2022 HDI ranged from 0.540 (Pakistan) to 0.670 (Bangladesh), IMR ranged from 26 (India) to 51/1000 (Pakistan), and MMR ranged from 103 (India) to 154/100 000 (Pakistan). For the present analysis, we have excluded the AMANHI-Tanzania cohort because it did not collect longitudinal maternal weight measurements at follow-up visits. The initial total number of unique participants across five cohorts was 17 330. Study enrolment dates among the participants in each cohort were as follows: August 2014–August 2017 (AMANHI-Bangladesh), August 2014–June 2018 (AMANHI-Pakistan), April 2015–May 2017 (PreSSMat), May 2015–August 2020 (GARBH-Ini) and August 2015–September 2017 (ZAPPS). Among the initial 17330 participants, 15 533 (89 .7%) had at least two valid maternal weight measurements in pregnancy and were therefore eligible for analysis. Participants were enrolled in the respective cohorts before 25 weeks of gestation as assessed by ultrasound examination. Maternal weight was measured by study staff using calibrated weighing machines. Visits with extreme maternal weight change as compared with the baseline weight (weight changes more extreme than −4 kg or +19 kg, based on the 1 and 99 percentiles, respectively) were considered invalid. We excluded 247 (1.6%) participants who had abortions, unknown neonatal vital status or who were pregnant with twins/multiples. In the few cases where data from more than one pregnancy were available for a given participant, we selected the first available pregnancy only. The final study population had 15 286 singleton pregnancies with at least two maternal weight measurements, a known birth outcome and available neonatal vital status. We also defined a subset of 12 995 participants from which to estimate GWG reference values. This was done by excluding a total of 2291 women whose baseline maternal weight was measured after 20 gestational weeks (n=115) or whose pregnancies ended preterm (n=1783) or whose foetus was stillborn (n=393) (figure 1).

### Ethics

The MOMI consortium represents a unique multinational collaboration of diverse birth cohorts, established by combining data from existing studies after their primary data collection was complete. Each participating cohort was originally conducted with approval from its respective Institutional Review Board and written informed consent obtained from all participants. Approval for the ZAPPS cohort was granted by the University of Zambia School of Medicine Biomedical Research Ethics Committee (reference number: 016-04-14) and the University of North Carolina School of Medicine (study number: 14–2113). Approval for PreSSMat was granted by the Research Review and Ethical Review Committees of the International Centre for Diarrhoeal Disease Research, Bangladesh (PR-16039). Approval for GARBH-Ini was granted by the Translational Health Science and Technology Institute; District Civil Hospital, Gurugram; and Safdarjung Hospital, New Delhi (ETHICS/GHG/2014/1.43). Approvals for the AMANHI study were obtained from the International Centre for Diarrhoeal Disease Research, Bangladesh, Aga Khan University, Pakistan; Zanzibar Medical Research and Ethics Committee, Tanzania; and John Hopkins University (Tanzania and Bangladesh). AMANHI protocols were also approved by the WHO Ethics Review Committee.^12^,^15^

### Patient and public involvement

Patients or the public were not involved in the design, conduct, reporting, or dissemination plans of our research.

### Longitudinal description of gestational weight gain (GWG)

For the first objective of estimating GWG reference values, the outcome of interest was change in maternal weight over the course of gestation. At each follow-up visit, maternal weight gain was calculated as measured weight minus baseline weight (kilograms). Baseline was defined as study enrolment or pre-enrolment screening visit, if available. Before modelling GWG longitudinally (described below), the original weight gain measurements were log-transformed (natural log) to minimise skewness. A constant of +5 kg was added to all weight gain measurements to remove negative values before the log transformation. The primary independent variable was gestational age (weeks) at each follow-up visit, and stratification variables were baseline BMI group (underweight, <18.5; normal, 18.5–24.9; overweight, 25–29.9; obese, ≥30) and study cohort (AMANHI-Bangladesh, AMANHI-Pakistan, PreSSMat, GARBH-Ini and ZAPPS).

### Statistical analysis

Frequencies and measures of centrality and distribution were used to describe the study population characteristics overall and by individual cohort. Mixed effects linear regression was used to model the change in gestational weight as a function of gestational age and baseline BMI group, separately by cohort, among participants in the reference subpopulation only. Specifically, we modelled the outcome of GWG with gestational age at follow-up visit as the primary independent variable along with covariates for the main effect of BMI group and the interaction between BMI group and the linear term for gestational age at the visit. The models had a participant level random intercept and a fixed effect for gestational age. To accommodate for non-linearity of effects, the models included restricted quadratic spline terms for gestational age, with cohort-specific basis functions (four knots at 5 p, 35 p, 65 p and 95 p).^1^ From these models, we obtained estimates of the mean and SD for GWG associated with each gestational week, which were specific by cohort and BMI group. Reference percentiles of GWG were then calculated by cohort and BMI group (online supplemental appendix table A1–A20).

### Gestational weight gain (GWG) and adverse outcomes

For the second objective of estimating the association between GWG and adverse pregnancy outcomes, the primary outcomes were: preterm birth (<37 gestational weeks, gestational dating done by ultrasound examination before 25 weeks of pregnancy), low birth weight (<2500 kg), small for gestational age (<10^th^ percentile based on IG-21 standards) and very small for gestational age (<3^rd^ percentile). The following secondary outcomes were included to describe the study population: preterm birth before 32 gestational weeks, birth weight <1500 kg, stillbirth and caesarean delivery. The primary independent variable was the total weight gain for gestational age Z-score, henceforth referred to as weight gain Z-scores. Z-scores were calculated from total GWG and categorised into groups that corresponded to the 0–25^th^, 26–50^th^, 51–75^th^ and 76–100^th^ percentiles of the standard normal distribution. The following covariates were included to adjust for potential confounding of the association between primary outcomes and weight gain Z-score: maternal age, gestational age at enrolment, maternal height, baseline BMI, parous, previous preterm birth, previous stillbirth and maternal years of education. Further details are provided below.

### Missing data

A small proportion of participants in the study population were missing values for either baseline covariates, such as previous preterm birth (1.5% missing), or outcomes, such as birth weight (12% missing) (details in online supplemental file 1). Therefore, we employed multiple imputation to account for the non-monotone missing data.^16^ Missing covariate and outcome data were imputed 50 times using a Markov chain Monte Carlo algorithm that depended on the following variables: maternal age, gestational age at enrolment, maternal height, baseline maternal weight, baseline BMI, parous, previous preterm birth, previous stillbirth, chronic hypertension, diabetes, maternal years of education, gestational age at last antenatal maternal weight measurement, gestational age at delivery, total GWG, GWG Z-score, spontaneous labour, caesarean delivery, hypertension or pre-eclampsia in labour, stillbirth, birth weight and infant sex. All subsequent analyses were performed for each imputed data set and then averaged (using Rubin’s rule to combined standard errors).^17^

### Statistical analysis

#### Gestational weight gain (GWG) Z-scores

Using the estimated mean and SD reference values, we calculated Z-scores of total weight gain for each participant in the overall study population. First, total weight gain for each participant was calculated as last weight before/at delivery minus baseline weight. Second, weight gain Z-scores were calculated as: (total weight gain – reference mean weight gain for the gestational week)/reference SD. These yielded standardised estimates of total weight gain which were specific to each participant’s cohort, BMI group and gestational age at last weight measurement. We then estimated the association between baseline characteristics and GWG-Z scores using marginal linear regression models. Each model included GWG Z-score as the outcome variable, and each individual baseline characteristic as the independent variable of interest. We used *Proc Genmod* in SAS software, with an identity link function, and robust standard errors with study cohort as the clusters. Continuous predictors were categorised using clinically relevant cut-offs or quartiles, as detailed in the online supplemental file 1.

#### Adjusted risk ratio of outcomes

We modelled each of the adverse pregnancy outcomes using Poisson regression with robust standard errors while assuming a binomial distribution.^18^ The primary independent variable was GWG Z-score groups. We categorised Z-scores into four groups that correspond to the cut-offs for the ≤25^th^, 26–50^th^, 51–75^th^, and >75^th^ percentiles of the standard normal distribution, respectively. For each outcome, we first estimated unadjusted risk ratios associated with GWG from a model that included GWG Z-score group as the only independent variable. We then estimated adjusted risk ratios (ARR) by adding covariates to the models that contained weight gain Z-score group. The multivariable modelling was done only for the primary outcomes of interest. We started out with a list of potential covariates for adjustment, which were selected a priori, based on clinical significance and availability across all sites. We then selected a smaller set of covariates for inclusion in adjustment for each outcome based on their association with GWG and each primary outcome in the study population (online supplemental file 1). Continuous covariates were modelled with restricted quadratic splines (three equally spaced knots) to account for non-linearities in the association with outcomes.^19^ The following covariates were included in multivariable models: maternal age, gestational age at enrolment, maternal height, baseline BMI, parous and previous preterm birth.

#### Additional analyses

To assess the nonlinear association between each of the primary outcomes and GWG Z-scores, we first modelled each outcome using marginal logistic regression (generalised estimating equations, GEE), with the weight gain Z-score as a continuous independent variable with restricted quadratic splines (three equally spaced knots) and the above-listed covariates. We obtained predicted probabilities of each outcome for each participant from the marginal logistic models. Next, generalised additive models were used to fit the non-linear curve between the predicted probabilities of each outcome and Z-scores by baseline BMI. We used the *gee, geepack* and *ggplot2* packages in R programming language (V.4.1.3). To assess the robustness of our findings, we repeated the outcome modelling separately by study cohort. We also repeated the outcome modelling among participants without a history of previous preterm birth. We report results from a complete case analysis for each primary outcome, where participants missing data are excluded, in the online supplemental file 1. Finally, we report the following ad hoc analyses in the online supplemental file 1: study population characteristics before exclusion of participants with <2 wt measurements, alternative measures of GWG (IOM adequacy ratio and IG-21 Z-scores) and their associations with outcomes in the study population.^6 9 20^ The supplement is organised as follows. Online supplemental appendix tables A1–A20 list cohort- and BMI-specific GWG percentiles. Online supplemental appendix tables B1–B9 describe cohort selection (B1), missing data (B2), GWG Z-score distribution (B3), reference subpopulation characteristics (B4), cohort-specific outcome ARRs associated with GWG Z-score groups (B5–B8) and outcome ARR among participants without previous preterm birth (B9). Online supplemental appendix figures B1–B7 show BMI- and cohort-specific plots of observed and predicted GWG in the reference subpopulation (B1–B5) and non-linear association plots between GWG Z-score and outcomes (B6–B13). Online supplemental appendix tables C1–C7 describe pre-imputation study population characteristics (C1), outcome risk and ARR (C2–C6) and study population characteristics before excluding participants with <2 wt measurements (C7). Online supplemental appendix tables D0–D5 and figures D1–D5 report results from using IOM GWG guidelines, while online supplemental appendix figure D6 and tables D8–13 report results from using IG-21 Z-scores.

**Table 1:** Study population characteristics and outcomes, overall and by cohort

	Overall(n=15 286)	4: AMANHI-Bangladesh(n=2786)	3: AMANHI-Pakistan(n=2025)	2: PreSSMat(n=3456)	6: GARBH-Ini(n=6004)	1: ZAPPS(n=1015)
Maternal age (years)	24.0 (21.0, 28.0)	23.0 (20.0, 26.0)	26.0 (23.0, 30.0)	25.0 (21.0, 29.0)	23.0 (21.0, 26.0)	27.0 (23.0, 32.0)
Gestational age at enrolment (weeks)	13.0 (11.0, 16.0)	13.4 (11.1, 16.6)	13.4 (10.7, 16.7)	12.1 (11.4, 13.6)	13.1 (9.6, 16.0)	16.3 (13.4, 18.4)
Gestational age at first maternal weight (weeks)	12.9 (10.9, 15.9)	12.4 (9.9, 15.7)	13.4 (10.7, 16.7)	12.1 (11.4, 13.6)	13.1 (9.6, 16.1)	16.3 (13.6, 18.4)
Maternal weight at baseline (kg)	48.3 (42.7, 55.6)	43.7 (40.0, 48.7)	50.5 (44.5, 60.0)	49.5 (44.4, 56.2)	47.7 (42.6, 54.2)	61.0 (54.0, 72.0)
Maternal height (cm)	152.5 (148.6, 156.6)	150.0 (146.2, 153.2)	153.5 (150.0, 158.0)	151.7 (148.2, 155.4)	153.1 (149.2, 156.9)	160.0 (156.0, 164.0)
BMI (kg/m^2^)	20.7 (18.6, 23.5)	19.5 (18.0, 21.4)	21.5 (18.7, 25.2)	21.5 (19.4, 24.3)	20.3 (18.4, 22.9)	23.9 (21.4, 27.6)
Underweight	23.1% (3536)	33.0% (920)	22.4% (453)	15.9% (549)	26.1% (1567)	4.6% (47)
Normal	60.0% (9178)	60.9% (1696)	51.6% (1044)	64.0% (2212)	61.3% (3678)	54.0% (548)
Overweight	13.4% (2048)	5.3% (148)	19.0% (384)	17.3% (598)	10.8% (649)	26.5% (269)
Obese	3.4% (524)	0.8% (22)	7.1% (144)	2.8% (97)	1.8% (110)	14.9% (151)
Parous (1+ previous delivery)	60.5% (9253)	64.2% (1790)	75.9% (1537)	63.0% (2176)	50.6% (3038)	70.1% (712)
Previous preterm birth*	11.2% (1035)	5.8% (103)	9.3% (143)	7.6% (166)	11.4% (346)	38.9% (277)
Previous stillbirth*	7.6% (702)	11.7% (210)	8.3% (128)	4.1% (90)	5.8% (175)	13.9% (99)
Maternal years of education	10.0 (6.0, 13.0)	7.0 (5.0, 9.0)	0.0 (0.0, 8.0)	8.0 (5.0, 10.0)	14.0 (12.0, 15.0)	12.0 (9.0, 12.0)
Chronic hypertension	1.9% (295)	0.2% (6)	5.7% (115)	1.0% (35)	0.3% (20)	11.8% (119)
Diabetes	0.4% (65)	0.3% (8)	0.9% (18)	0.5% (17)	0.2% (12)	1.0% (10)
Total maternal weight measurements	4.0 (3.0, 5.0)	4.0 (3.0, 4.0)	3.0 (3.0, 4.0)	5.0 (4.0, 5.0)	4.0 (3.0, 5.0)	5.0 (4.0, 5.0)
Gestational age at last maternal weight	36.4 (32.4, 38.6)	36.6 (34.0, 37.6)	37.7 (32.7, 38.6)	36.0 (33.0, 38.6)	37.1 (30.7, 39.4)	36.1 (35.4, 36.6)
Total GWG (kg)	6.8 (4.2, 9.4)	6.1 (3.7, 8.5)	6.5 (3.8, 9.5)	7.3 (5.1, 9.8)	6.7 (4.1, 9.5)	7.0 (4.0, 10.0)
Total GWG, participants w/ both second and third trimester weight†	7.2 (4.8, 9.8)	6.3 (3.9, 8.7)	7.0 (4.3, 9.9)	7.6 (5.4, 10.0)	7.4 (5.1, 10.0)	7.4 (4.6, 10.6)
2nd trimester GWG (kg)†	3.4 (1.9, 5.2)	2.9 (1.5, 4.6)	3.5 (1.8, 5.5)	3.5 (2.1, 5.1)	3.7 (2.1, 5.5)	3.0 (1.0, 5.2)
3rd trimester GWG (kg)†	7.1 (4.8, 9.7)	6.3 (3.9, 8.7)	7.0 (4.3, 9.9)	7.5 (5.3, 10.0)	7.3 (5.0, 9.8)	7.4 (4.5, 10.6)
GWG Z-score percentile‡	53.7 (29.1, 74.8)	55.9 (32.1, 73.8)	55.1 (30.6, 73.4)	53.6 (27.7, 76.4)	52.4 (27.9, 75.1)	52.3 (28.1, 74.5)
GWG Z-score percentile (normal BMI)§	53.9 (28.7, 74.4)	56.9 (31.8, 75.3)	55.2 (32.0, 71.4)	53.4 (27.2, 76.0)	52.4 (27.8, 74.2)	52.3 (28.1, 71.3)
IG-21 GWG Z-score percentile (normal BMI)§¶^¶^	9.8 (1.7, 29.2)	4.4 (0.4, 17.7)	9.3 (1.2, 27.3)	13.4 (3.1, 34.2)	11.1 (2.4, 29.6)	14.6 (1.9, 43.3)
IOM adequacy ratio**	60.9 (40.7, 84.4)	51.4 (31.8, 70.3)	63.2 (38.1, 88.9)	67.6 (48.5, 89.8)	59.6 (40.6, 82.5)	74.5 (42.9, 111.4)
IOM adequacy ratio (normal BMI)§	58.8 (38.6, 79.9)	50.6 (30.2, 70.6)	59.7 (36.7, 80.2)	64.9 (46.2, 84.9)	57.7 (37.4, 77.8)	67.6 (42.0, 92.8)
Gestational age at delivery (weeks)	39.1 (38.0, 40.0)	39.1 (38.0, 40.0)	39.0 (37.7, 39.9)	39.0 (38.0, 39.9)	39.1 (38.0, 40.0)	39.6 (38.4, 40.4)
Spontaneous labour	78.5% (12003)	86.5% (2410)	82.2% (1665)	52.7% (1821)	88.0% (5281)	81.4% (826)
Caesarean delivery	26.6% (4060)	13.5% (376)	17.8% (359)	47.4% (1639)	24.2% (1454)	22.8% (231)
Preterm birth <37 weeks	13.0% (1993)	13.1% (364)	15.3% (309)	11.8% (407)	13.2% (790)	12.1% (123)
Preterm birth <32 weeks	1.4% (217)	1.3% (36)	1.1% (22)	0.8% (28)	1.7% (101)	3.0% (30)
Birth weight	2800 (2500, 3092)	2700 (2400, 3000)	2760 (2485, 3050)	2845 (2553, 3140)	2760 (2490, 3040)	3035 (2760, 3380)
Birth weight <2500 g	24.6% (3758)	31.9% (887)	25.5% (516)	19.3% (668)	25.9% (1553)	13.2% (134)
Birth weight <1500 g	1.6% (249)	1.4% (38)	1.2% (24)	1.0% (35)	2.0% (122)	3.1% (31)
SGA <10th centile††	35.8% (5471)	44.2% (1230)	34.6% (702)	29.6% (1021)	39.1% (2338)	17.8% (180)
SGA <3rd centile††	16.8% (2570)	22.8% (635)	15.7% (318)	12.1% (418)	18.8% (1125)	7.4% (75)
Stillbirth	2.6% (396)	3.7% (102)	3.2% (65)	1.5% (52)	2.3% (141)	3.5% (36)

Data shown as % (n) or median (IQR)

* Among N=9253 parous participants.

† Among N=12938 participants with available second and third trimester weight gain

‡ Weight gain for gestational age Z-score based on BMI- and cohort-specific reference GWG values estimated from the study subpopulation

§ Restricted to N=8383 normal BMI participants with final weight measurement at 14–40 weeks of gestation (the target population of IG-21 weight gain for gestational age standards) (https://doi.org/10.1136/bmj.i555)

¶ Weight gain for gestational age Z-score based on IG-21 weight gain for gestational age standards (https://doi.org/10.1136/bmj.i555)

** Ratio of (observed/recommended weight gain)*100. Recommended weight gain calculated as: 1st trimester recommended weight gain + [2nd & 3rd trimester recommended weekly weight gain rate*(final gestational age weeks-13)]. We assumed a 1st trimester weight gain of 2kg for underweight/normal BMI participants and of 0.5kg for overweight/obese. We assumed a second and third trimester weight gain rate of 0.51, 0.42, 0.28 and 0.22 kg/week for underweight, normal, overweight and obese respectively. IOM 2009 guidelines: https://doi.org/10.17226/12584.

†† Among N=15265 with available newborn weight centile data; N=21 excluded due to gestational age outside the IG-21 range of 24+0 and 42+6 weeks’ gestation (N=1 in PreSSMat; N=17 in GARBH-Ini; and N=3 in ZAPPS).

AMANHI, Alliance for Maternal and Neonatal Health Improvement; BMI, body mass index; GARBH-Ini, Group for Advanced Research on Birth outcomes – DBT India Initiative; GWG, Gestational weight gain; IG-21, INTERGROWTH-21st; IOM, Institute of Medicine; PreSSMat, Preterm and Stillbirth Study; SGA, small-for-gestational-age; ZAPPS, Zambian Preterm Birth Prevention Study.

## Results

Among the 15 286 participants in the study population, a combined 41% were from Bangladesh (23% PreSSMat, 18% AMANHI-Bangladesh), 39% from India (GARBH-Ini), 13% from Pakistan (AMANHI-Pakistan) and 7% from Zambia (ZAPPS). Overall, the median maternal age was 24 years (IQR 21, 28) across all cohorts but varied from 23 years (IQR 20, 26; AMANHI-Bangladesh) to 27 years (IQR 23, 32; ZAPPS). The median gestational age at enrolment was 13 weeks (IQR 11, 16), with cohort-specific values ranging from 12.1 weeks (IQR 11.4, 13.6; PreSSMat) to 16 weeks (IQR 13, 18; ZAPPS). The median gestational age at first maternal weight measurement was 12.9 weeks (IQR 10.9, 15.9) and ranged from 12.1 (IQR 11.4, 13.6; PreSSMat) to 16.3 (IQR 13.6, 18.4; ZAPPS). The majority of participants had normal BMI (60% across all sites, ranging from 54% in ZAPPS to 64% in PreSSMat). The proportion of underweight BMI (23%) varied from 5% (ZAPPS) to 33% (AMANHI-Bangladesh). The remaining three cohorts all had >15% prevalence of underweight BMI. The overall proportion of participants who had one or more previous deliveries (parous participants) was 61%, with cohort-specific proportions ranging from 51% (GARBH-Ini) to 76% (AMANHI-Pakistan) (table 1).

**Figure 2:**
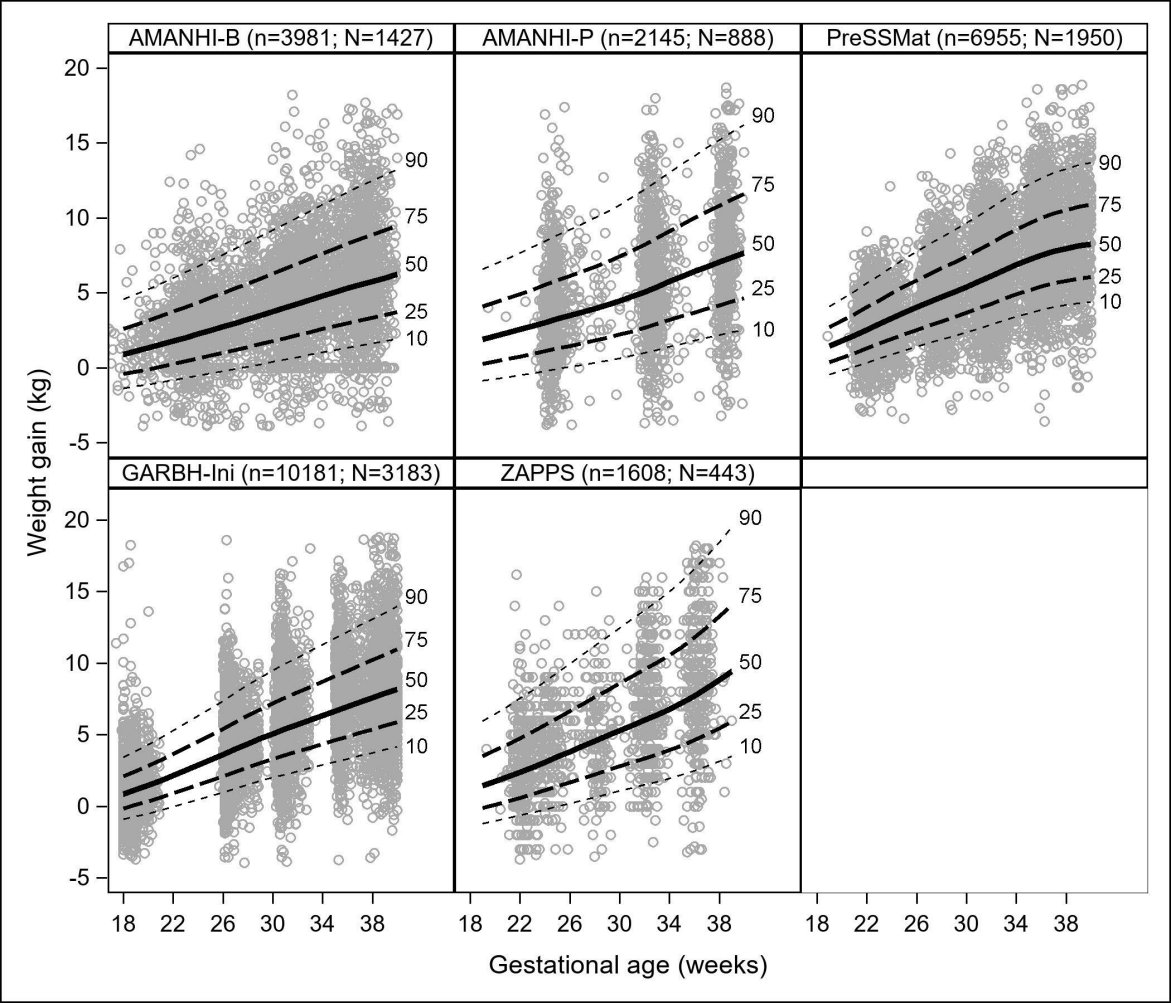
Observed (circles) and predicted values (curves) for maternal weight gain in the reference sub-population, among normal BMI participants only

The median number of maternal weight measurements during follow-up was 4 (IQR 3, 5), ranging across cohorts from three in AMANHI-Pakistan to five in PreSSMat and ZAPPS. The median total GWG among the 15 286 participants was 6.8 kg (IQR 4.2, 9.4). The cohort-specific median GWG ranged from 6.1 kg (IQR 3.7, 8.5) in AMANHI-Bangladesh to 7 kg (IQR 4, 10) in ZAPPS. The overall median GWG for second and third trimesters were 3.4 kg (IQR 1.9, 5.2) and 7.1 kg (IQR 4.8, 9.7), respectively, among 12 938 participants with both second and third trimesters weight gain information (table 1). Detailed estimates of the percentiles of GWG by study cohort and BMI group are presented in the online supplemental file 1. An example of plots of observed and predicted values for weight gain among normal BMI participants in each cohort is shown in figure 2. After standardisation, the mean weight gain Z-score was −0.02 overall (95% CI −0.03 to –0.01). Between-cohort differences in mean GWG Z-scores were negligible, ranging from −0.06 (95% CI −0.13, 0.00) in ZAPPS to 0.00 (95% CI −0.03, 0.04) in PreSSMat. GWG Z-scores were lower among those with advanced maternal age (35+ years; −0.22), those in the highest quartile of gestational age at enrolment (16–24 weeks at enrolment; −0.21) and those with previous preterm birth (−0.13) (online supplemental appendix table B3).

### Gestational weight gain (GWG and adverse pregnancy outcomes

The overall risk of preterm birth <37 weeks was 13%. Cohort-specific estimates ranged from 11.8% (PreSSMat) to 15.3% (AMANHI-Pakistan) (table 1). Among underweight participants, preterm birth risk was higher in both the ≤25^th^ (17.6%; ARR 1.4; 95% CI 1.1, 1.9) and 26–50^th^ GWG percentile groups (13.7%; ARR 1.1; 95% CI 1.0, 1.3), when compared with the 51–75^th^ percentiles (12.3%). Among normal weight participants, risk estimates were higher in both the ≤25^th^ (11.9%) and 26–50^th^ (12.7%) GWG percentile groups (ARR 1.1; 95% CI 1.0, 1.2; and ARR 1.2; 95% CI 1.1, 1.3; respectively) when compared with the 51–75^th^ percentiles (10.8%). Among overweight participants, there were no clear differences in the risk for preterm birth associated with GWG, with percentile group estimates ranging from 12% (51–75^th^) to 13.4% (>75^th^). Similarly, differences were minimal among the obese, with preterm birth risk estimates ranging from 14.2% (≤25^th^) to 15.8% (26–50^th^) (table 2). In cohort-specific analyses, among underweight participants, the risk of preterm birth was highest for the ≤25^th^ GWG percentile group in GARBH-Ini and AMANHI-Pakistan only. Among normal weight participants, results in AMANHI-Pakistan showed higher preterm birth risk in the ≤50^th^ and >75^th^ GWG percentile groups, but there were no such patterns in the other four cohorts (online supplemental appendix table B5).

**Table 2:** Association between gestational weight gain Z-score group and adverse outcomes by BMI

	BMI	Weight gain z-score percentile*	Risk (n/N)	Adjusted risk† % (95% CI)	Adjusted risk ratio† (95% CI)
Preterm birth	Underweight	≤25	123/690	17.6 (13.5, 23.1)	1.44 (1.09, 1.91)
		26–50	147/1002	13.7 (11.6, 16.2)	1.12 (1.00, 1.24)
		51–75	147/1126	12.3 (11.5, 13.1)	1
		>75	96/718	12.1 (9.8, 14.8)	0.99 (0.82, 1.19)
	Normal	≤25	254/2000	11.9 (11.3, 12.4)	1.10 (1.02, 1.17)
		26–50	295/2199	12.7 (12.2, 13.3)	1.18 (1.08, 1.29)
		51–75	304/2701	10.8 (10.1, 11.6)	1
		>75	254/2278	10.7 (9.5, 12.1)	0.99 (0.82, 1.19)
	Overweight	≤25	68/497	12.5 (9.5, 16.3)	1.04 (0.70, 1.53)
		26–50	58/395	14.0 (11.8, 16.6)	1.16 (0.89, 1.52)
		51–75	69/528	12.0 (9.3, 15.6)	1
		>75	91/628	13.4 (11.2, 16.0)	1.11 (0.82, 1.51)
	Obese	≤25	19/128	14.2 (8.5, 23.6)	0.95 (0.46, 1.96)
		26–50	16/93	15.8 (11.8, 21.1)	1.06 (0.45, 2.53)
		51–75	20/118	14.9 (7.7, 28.8)	1
		>75	32/185	15.3 (11.9, 19.6)	1.03 (0.53, 1.99)
Low birth weight	Underweight	≤25	317/690	45.3 (39.0, 52.7)	1.66 (1.47, 1.89)
		26–50	367/1002	35.3 (28.9, 43.1)	1.29 (1.15, 1.45)
		51–75	316/1126	27.3 (24.2, 30.7)	1
		>75	164/718	21.7 (19.0, 24.7)	0.79 (0.70, 0.90)
	Normal	≤25	643/2000	30.9 (27.9, 34.2)	1.49 (1.40, 1.57)
		26–50	575/2199	24.7 (22.5, 27.2)	1.19 (1.06, 1.33)
		51–75	592/2701	20.8 (18.7, 23.1)	1
		>75	362/2278	15.1 (12.0, 19.0)	0.73 (0.61, 0.87)
	Overweight	≤25	106/497	19.1 (14.7, 24.9)	1.35 (1.04, 1.75)
		26–50	75/395	17.7 (16.1, 19.4)	1.25 (1.06, 1.48)
		51–75	84/528	14.1 (12.0, 16.7)	1
		>75	78/628	11.8 (10.1, 13.8)	0.83 (0.70, 0.99)
	Obese	≤25	25/128	17.7 (9.8, 32.3)	1.80 (0.75, 4.35)
		26–50	16/93	15.1 (9.3, 24.4)	1.53 (0.64, 3.66)
		51–75	15/118	9.8 (6.2, 15.8)	1
		>75	23/185	9.6 (7.2, 12.8)	0.98 (0.62, 1.55)
Small for gestational age <10th	Underweight	≤25	400/690	55.7 (53.0, 58.6)	1.31 (1.21, 1.42)
		26–50	504/1001	49.2 (42.0, 57.7)	1.16 (1.05, 1.27)
		51–75	482/1124	42.5 (39.1, 46.1)	1
		>75	255/718	35.3 (30.3, 41.1)	0.83 (0.69, 1.00)
	Normal	≤25	921/1996	45.5 (42.0, 49.3)	1.44 (1.35, 1.52)
		26–50	865/2196	37.8 (35.4, 40.4)	1.19 (1.10, 1.29)
		51–75	896/2696	31.7 (28.2, 35.6)	1
		>75	598/2275	25.1 (22.2, 28.4)	0.79 (0.73, 0.86)
	Overweight	≤25	144/497	27.2 (24.7, 29.9)	1.33 (1.17, 1.51)
		26–50	97/395	23.5 (21.1, 26.2)	1.15 (1.01, 1.30)
		51–75	120/527	20.5 (18.6, 22.6)	1
		>75	108/628	16.1 (13.2, 19.7)	0.79 (0.67, 0.92)
	Obese	≤25	25/126	19.3 (13.1, 28.4)	1.70 (1.13, 2.55)
		26–50	18/93	17.6 (11.7, 26.5)	1.55 (0.98, 2.45)
		51–75	16/118	11.4 (6.8, 19.0)	1
		>75	21/185	10.0 (7.2, 13.8)	0.88 (0.46, 1.65)
Small for gestational age <3rd	Underweight	≤25	219/690	29.7 (27.2, 32.6)	1.55 (1.37, 1.74)
		26–50	272/1001	25.9 (21.1, 31.9)	1.35 (1.09, 1.66)
		51–75	222/1124	19.3 (16.3, 22.7)	1
		>75	109/718	14.8 (12.4, 17.7)	0.77 (0.68, 0.87)
	Normal	≤25	458/1996	21.9 (19.5, 24.5)	1.52 (1.41, 1.63)
		26–50	394/2196	16.5 (13.5, 20.2)	1.15 (0.98, 1.34)
		51–75	425/2696	14.4 (12.6, 16.4)	1
		>75	238/2275	9.6 (7.4, 12.5)	0.66 (0.57, 0.77)
	Overweight	≤25	60/497	9.5 (7.0, 12.7)	1.26 (0.82, 1.94)
		26–50	34/395	6.9 (5.0, 9.6)	0.93 (0.66, 1.30)
		51–75	53/527	7.5 (6.1, 9.1)	1
		>75	40/628	5.4 (4.2, 6.9)	0.72 (0.60, 0.87)
	Obese	≤25	14/126	9.8 (7.0, 13.8)	1.51 (0.74, 3.10)
		26–50	9/93	8.2 (4.9, 13.6)	1.26 (0.66, 2.42)
		51–75	9/118	6.5 (3.8, 11.0)	1
		>75	14/185	6.0 (4.6, 7.9)	0.93 (0.56, 1.56)

* Z-score cut-offs: ≤ −0.67 (≤ 25th percentile); −0.68–0.00 (26–50th percentile); 0.01–0.67 (51–75th percentile); > 0.067 (> 75th percentile)

† Adjusted for maternal age, gestational age at enrolment, maternal height, maternal BMI, parous and previous preterm birth

BMI, body mass index.

The overall risk of low birth weight <2500 g was 24.6%. Cohort-specific estimates of low birth weight ranged from 13.2% (ZAPPS) to 31.9% (AMANHI-Bangladesh) (table 1). Among underweight participants, the risk of low birth weight increased in a stepwise manner when going from the highest to the lowest GWG Z-score percentile group. The risk was highest for those in the ≤25^th^ GWG percentile (45.3% adjusted risk; ARR 1.7; 95% CI 1.5, 1.9) and lowest for those in the >75^th^ GWG percentile (21.7%; ARR 0.8; 95% CI 0.7, 0.9) when compared with those in the 51–75^th^ GWG percentile group (27.3% risk). This pattern of higher risk associated with lower GWG also occurred among normal weight and overweight participants, although risk estimates were lower among normal and overweight. Among the obese, the risk and ARR estimates of low birth weight were generally imprecise (table 2). Results from cohort-specific analyses were consistent with those observed in the overall study population, with some variation in the estimates and wider confidence intervals throughout (online supplemental appendix table B6).

The overall risk of small for gestational age <10^th^ percentile was 35.8%. Cohort-specific estimates ranged from 17.8% (ZAPPS) to 44.2% (AMANHI-Bangladesh) (table 1). Among underweight participants, the risk of small for gestational age ranged from 35.3% in the >75^th^ GWG percentile group (ARR 0.8; 95% CI 0.7, 1.0) to 55.7% in the ≤25^th^ percentile group (ARR 1.3; 95% CI 1.2, 1.4). Among normal weight participants, risk estimates ranged from 25.1% in the >75^th^ GWG percentile group (ARR 0.8; 95% CI 0.7, 0.9) to 45.6% in the ≤25^th^ percentile group (ARR 1.4; 95% CI 1.4, 1.5). Among overweight participants, risk estimates ranged from 16.1% in the >75^th^ GWG percentile group (ARR 0.8; 95% CI 0.7, 0.9) to 27.2% in the ≤25^th^ percentile group (ARR 1.3; 95% CI 1.2, 1.5). Among obese participants, risk estimates ranged from 10% in the >75^th^ GWG percentile group (ARR 0.9; 95% CI 0.5, 1.7) to 19.3% in the ≤25^th^ percentile group (ARR 1.7; 95% CI 1.31, 2.6) (table 2). In cohort-specific analyses, the pattern of higher risk associated with lower GWG was clear among normal weight participants across all sites and, to a lesser extent, among underweight participants in PreSSMat, AMANHI-Bangladesh and GARBH-Ini (online supplemental appendix table B7). Results for overall and cohort-specific risk and ARR estimates for small for gestational age <3^rd^ percentile had similar patterns to those observed for small for gestational age <10^th^ (online supplemental appendix tables 2 and B8).

In general, the above findings were supported by the plots for non-linear associations conducted to evaluate the dose-response relationship between GWG Z-score, as a continuous variable and each of the primary outcomes (online supplemental appendix figures B6–B13 B6-B13). As the GWG Z-score increased, the risk of preterm birth had a clear decreasing trend among underweight participants and a modest decreasing trend among normal BMI participants and less clear trends for overweight and obese participants (with the exception of an increasing trend among the obese in AMANHI-Pakistan that is likely to be due to very low sample size). Consistent with the above-listed risk ratios, the non-linear association plots for low birth weight and small for gestational age showed decreasing risk of outcome with increasing GWG Z scores across all BMI strata but with the clearest trends among underweight and normal BMI participants. Results from analyses restricted to participants without previous preterm birth were consistent with the overall results (online supplemental appendix table B9). Results from complete case analyses were consistent with those from the analyses on multiply-imputed data, with the latter having narrower confidence intervals around the estimates (online supplemental appendix table C3–C6).

### Institute of Medicine (IOM) adequacy ratio

Only 14% of study participants met the IOM-defined adequate range of total GWG across all sites and BMI strata, with cohort-specific proportions ranging from 7.6% (AMANHI-Bangladesh) to 23.3% (ZAPPS). A total of 88.3% of underweight and 87.3% of normal BMI participants fell below the adequate GWG range (online supplemental appendix table D0). After accounting for each participant’s length of gestation, the median GWG adequacy ratio was 61 (IQR 41–84), meaning that 75% of participants had a GWG that was within 84% or less of the recommended GWG. The cohort-specific median GWG adequacy ratio ranged from 52 (IQR 32, 70; AMANHI-Bangladesh) to 75 (IQR 43, 111; ZAPPS) (table 1). After grouping participants into BMI- and cohort-specific quartiles of GWG adequacy ratio, we found results for the association with adverse outcomes that were consistent with those from our study GWG Z-scores. Lower adequacy ratios were associated with increased risk of preterm birth among underweight (17.6% risk; ARR: 1.52; 95% CI 1.27, 1.81 for lowest quartile group) and normal BMI participants (14.5% risk; ARR 1.51; 95% CI 1.42, 1.61 for lowest quartile group). Lower GWG adequacy ratios were associated with increased risk of low birth weight in a stepwise manner among underweight (43.2% risk; ARR 1.55; 95% CI 1.43, 1.69 for lowest quartile group), normal BMI (31.3% risk; ARR 1.59; 95% CI 1.45, 1.74 for lowest quartile group) and overweight participants (18.9% risk; ARR 1.46; 95% CI 1.10, 1.95 for lowest quartile group). Patterns for small for gestational age outcomes were similar to those for low birth weight. Cohort-specific estimates for the association with preterm birth and the distribution of adequacy ratios are provided in online supplemental file 1.

### INTERGROWTH-21^st^ (IG-21) Z-scores

The overall median IG-21 GWG percentile was 9.8 (IQR 1.7, 29.2) among normal BMI participants, meaning that 75% of women with normal BMI had a total GWG below the 30^th^ percentile of IG-21 standards. The cohort-specific median IG-21 percentile ranged from 4.4 (IQR 0.4, 17.7) in AMANHI-Bangladesh to 14.6 (IQR 1.9, 43.3) in ZAPPS. Thus, in general, the IG-21 GWG Z-score standards were much higher than our study-specific Z-score reference values. For instance, our study-specific 50^th^ percentile for GWG at 40 weeks of gestation ranged from 6.2 kg (AMANHI-Bangladesh) to 9.4 kg (ZAPPS), in contrast to the IG-21 standard of 13.2 kg. We then categorised the IG-21 GWG Z-scores into four groups that correspond to the cut-offs for the 0–25^th^, 26–50^th^, 51–75^th^ and 76–100^th^ percentiles of the standard normal distribution. The resulting IG-21 Z-score groups were imbalanced with 71% of participants (n=5978) grouped in the ≤25^th^ group, and just 4% (n=309) grouped in the >75^th^ percentile group. Results for the association between IG-21 GWG Z-score groups and preterm birth were unclear and lacked consistency with those from our study-specific Z-scores. The risk of preterm birth was similar across the ≤25^th^ (12.5%), 26–50^th^ (12.3%) and 51–75^th^ (12.1%) percentile groups, but increased for the >75^th^ percentile (17.7% risk; ARR 1.46; 95% CI 1.10, 1.95). Results for the ARR of low birth weight and small for gestational age associated with IG-21 Z-score groups were consistent with those from our study-specific Z-scores, although point estimates for the risk in each IG-21 group were lower. The ≤25^th^ percentile of IG-21 group had the highest risk of low birth weight (26.6% risk; ARR 1.61; 95% CI 1.31, 1.99) and small for gestational age <10^th^ (37.6% risk; ARR 1.57; 95% CI 1.33, 1.86). Details are presented in the online supplemental appendix D.

## Discussion

We estimated reference values of weight gain for gestational age reference in five prospective pregnancy cohorts in South Asia and Sub-Saharan Africa. We found that lower GWG Z-scores were associated with increased risk of preterm birth, low birth weight and small for gestational age. Whereas the association with small for gestational age occurred across all maternal baseline BMI strata, the association with low birth weight occurred among underweight, normal and overweight, but not obese participants. The association with preterm birth was evident only among underweight and normal weight participants.

Several studies have examined the association between GWG and pregnancy outcomes, but the overwhelming majority of reports focus on North American, European and East Asian populations.^7 20^ Our study focuses on pregnant women in Bangladesh, India, Pakistan and Zambia and provides reference charts of GWG. Given the population-based design of the cohorts included in this analysis, we expect our findings to be generalisable to populations in South Asia and Sub-Saharan Africa. While we demonstrate that our study GWG charts can be used to evaluate outcomes, the use of these charts as prescriptive curves is not warranted given that our observational cohorts had high prevalence of adverse outcomes.

Our results confirm that the extent of GWG and its association with adverse pregnancy outcomes is influenced by early pregnancy BMI. The largest difference in preterm birth risk associated with GWG was observed among underweight participants with estimates of 18% and 12% in the lowest versus highest GWG Z-score groups, respectively. In contrast, the difference in preterm birth risk between lowest versus highest GWG Z-score groups was negligible among normal weight participants (12% vs 11%). These trends are consistent with evidence from European and North American populations.^21 22^ We note that even among participants with normal baseline BMI and GWG Z-scores in the 51–75^th^ percentile, a subgroup expected to have favourable nutritional status, the preterm birth risk was still as high as 11%. This reminds us that there are other important risk factors beyond early pregnancy BMI and GWG in the study population. Nevertheless, our data are an initial step towards identifying opportunities for risk reduction related to GWG in LMIC settings.

Although the cohort-specific findings were generally consistent with results from the combined overall study population, there were some differences between cohorts in the association between GWG Z-score as a continuous variable and the risk of preterm birth. Specifically, among women who were overweight or obese, we observed a trend of increasing risk of preterm birth in the AMANHI-Pakistan and ZAPPS-Zambia cohorts. The reasons for these site-specific variations remain unclear and require further investigation. We acknowledge a lack of precision in risk estimates due to small site-specific sample size for obese participants (n=151 or less). The lack of a balanced sample size across all cohorts limits our ability to assess the extent to which contextual differences in each cohort drive the overall findings. However, the overall similarity in trends displayed in the non-linear risk plots point to robustness of the findings.

We focused our primary analysis on GWG Z-scores derived from reference values within our study population. In secondary analyses, we used two alternative measures of GWG that are based on external standards, namely the GWG adequacy ratio (based on^6^ guidelines) and the IG-21 Z-score.^6 9^ We found that the majority of our study participants only reached 84% or less than the IOM-recommended GWG (the median adequacy ratio was 61; IQR 41–84). Among normal BMI participants, we also found that 75% had a GWG Z-score below the 30^th^ percentile of IG-21 standards. Grouping the adequacy ratio into quartiles resulted in associations with preterm birth, low birth weight and small for gestational age risks that were consistent with our study-specific Z-scores. Grouping the IG-21 Z-scores into four groups defined by quartile cut-offs of the normal distribution (≤25^th^, 26–50^th^, 51–75^th^, >75^th^) also resulted in associations with low birth weight and small for gestational age that were consistent with those from our study-specific Z-scores. Unlike our study-specific GWG Z-scores, we did not find an increased risk of preterm birth in the lowest GWG group defined by IG-21 Z-scores. This may be due to the fact that 40% of our study participants were grouped in the lowest IG-21 GWG group and that a cut-off lower than the ≤25^th^ percentile for this measure would better identify the higher-risk participants.

Our study has limitations. First, we estimated GWG as the difference between last weight measurement at/before delivery and first available weight measurement during antenatal follow-up, rather than prepregnancy weight. As a consequence, our estimates of the total GWG are likely to be underestimates of the net GWG throughout the entire pregnancy starting at conception. However, this underestimation would be minimal as most participants were enrolled before 20 weeks’ gestation. Further, the maternal weight measurements were directly collected prospectively by dedicated study staff with calibrated instruments in each cohort, ensuring measurement accuracy and reliability. Second, we are unable to differentiate between spontaneous and provider-initiated preterm birth due to lack of information about type of preterm birth across sites. We therefore refrained from inferring the implications for preventing spontaneous preterm birth, which generally accounts for at least two-thirds of all preterm births.^23^ In addition, the observational cohort design limits our ability to understand the mechanisms behind the observed associations between GWG and pregnancy outcomes and to discern causality in the observed associations. The potential mechanisms for an association between GWG, preterm birth, low birth weight and small for gestational age are complex and include maternal body composition, nutritional and metabolic components, immune function and inflammation, placental development and function, and foetal growth. Furthermore, these pathways can be modified by underlying maternal chronic disease, behavioural factors, and social and environmental factors.^6^ We adjusted for a set of maternal covariates, identified from the literature, that were measured consistently across all sites to reduce confounding bias. Notably, we did not adjust for chronic hypertension or prepregnancy diabetes because these characteristics were not associated with GWG Z-score in the study population. However, we cannot rule out residual confounding due to unmeasured factors such as low socioeconomic status.^6^

## Conclusion

GWG was associated with preterm birth, low birth weight and small for gestational age in five cohorts of pregnant women in South Asia and Sub-Saharan Africa. The magnitude and direction of association between GWG and adverse pregnancy outcomes differed by outcome and by baseline maternal BMI. Our findings suggest that GWG is an important risk factor for adverse pregnancy outcomes and that early pregnancy BMI modifies the risks associated with GWG in these populations.

## Supplementary material

10.1136/bmjph-2024-000900 online supplemental file 1

## Data Availability

Data are available upon reasonable request.

## References

[1] Blencowe H, Cousens S, Oestergaard MZ (2012). National, regional, and worldwide estimates of preterm birth rates in the year 2010 with time trends since 1990 for selected countries: a systematic analysis and implications. The Lancet.

[2] Liu L, Oza S, Hogan D (2016). Global, regional, and national causes of under-5 mortality in 2000–15: an updated systematic analysis with implications for the Sustainable Development Goals. The Lancet.

[3] Blencowe H, Krasevec J, de Onis M (2019). National, regional, and worldwide estimates of low birthweight in 2015, with trends from 2000: a systematic analysis. Lancet Glob Health.

[4] Katz J, Lee AC, Kozuki N (2013). Mortality risk in preterm and small-for-gestational-age infants in low-income and middle-income countries: a pooled country analysis. The Lancet.

[5] Lee AC, Kozuki N, Cousens S (2017). Estimates of burden and consequences of infants born small for gestational age in low and middle income countries with INTERGROWTH-21^st^ standard: analysis of CHERG datasets. BMJ.

[6] IOM (Institute of Medicine), NRC (National Research Council) (2009). Weight Gain During Pregnancy: Reexamining the Guidelines.

[7] Goldstein RF, Abell SK, Ranasinha S (2017). Association of Gestational Weight Gain With Maternal and Infant Outcomes: A Systematic Review and Meta-analysis. JAMA.

[8] Perumal N, Wang D, Darling AM (2023). Suboptimal gestational weight gain and neonatal outcomes in low and middle income countries: individual participant data meta-analysis. BMJ.

[9] Ismail LC, Bishop DC, Pang R (2016). Gestational Weight Gain Standards Based on Women Enrolled in the Fetal Growth Longitudinal Study of the INTERGROWTH-21st Project. bmj.

[10] Contrepois K, Chen S, Ghaemi MS (2022). Prediction of gestational age using urinary metabolites in term and preterm pregnancies. Sci Rep.

[11] Thiruvengadam R, Desiraju BK, Natchu UCM (2022). Gestational weight gain trajectories in GARBH-Ini pregnancy cohort in North India and a comparative analysis with global references. Eur J Clin Nutr.

[12] Baqui AH, Khanam R, AMANHI (Alliance for Maternal and Newborn Health Improvement) Bio–banking Study group) (2017). Understanding biological mechanisms underlying adverse birth outcomes in developing countries: protocol for a prospective cohort (AMANHI bio-banking) study. J Glob Health.

[13] Bhatnagar S, Majumder PP, Salunke DM (2019). A Pregnancy Cohort to Study Multidimensional Correlates of Preterm Birth in India: Study Design, Implementation, and Baseline Characteristics of the Participants. Am J Epidemiol.

[14] Castillo MC, Fuseini NM, Rittenhouse K (2018). The Zambian Preterm Birth Prevention Study (ZAPPS): Cohort characteristics at enrollment. Gates Open Res.

[15] Hawken S, Ducharme R, Murphy MSQ (2023). Development and external validation of machine learning algorithms for postnatal gestational age estimation using clinical data and metabolomic markers. PLoS One.

[16] Perkins NJ, Cole SR, Harel O (2018). Principled Approaches to Missing Data in Epidemiologic Studies. Am J Epidemiol.

[17] Harel O, Mitchell EM, Perkins NJ (2018). Multiple Imputation for Incomplete Data in Epidemiologic Studies. Am J Epidemiol.

[18] Zou G (2004). A modified poisson regression approach to prospective studies with binary data. Am J Epidemiol.

[19] Howe CJ, Cole SR, Westreich DJ (2011). Splines for Trend Analysis and Continuous Confounder Control. Epidemiology (Sunnyvale).

[20] Hutcheon JA, Bodnar LM, Joseph KS (2012). The bias in current measures of gestational weight gain. Paediatr Perinat Epidemiol.

[21] Voerman E, Santos S, LifeCycle Project-Maternal Obesity and Childhood Outcomes Study Group (2019). Association of Gestational Weight Gain With Adverse Maternal and Infant Outcomes. JAMA.

[22] Bodnar LM, Hutcheon JA, Parisi SM (2015). Comparison of gestational weight gain z-scores and traditional weight gain measures in relation to perinatal outcomes. Paediatr Perinat Epidemiol.

[23] Goldenberg RL, Culhane JF, Iams JD (2008). Epidemiology and causes of preterm birth. The Lancet.

