# Supplementary material for: Association between gestational weight gain and adverse pregnancy outcomes: cohort analysis from South Asia and Sub-Saharan Africa
Source: BMJ Public Health. 2025 Feb 4;3(1):e000900. doi: 10.1136/bmjph-2024-000900 (PMC12107451; doi:10.1136/bmjph-2024-000900)
Supplement: online supplemental file 1 [file bmjph-3-1-s001.pdf]

SUPPLEMENTARY MATERIAL

GESTATIONAL WEIGHT GAIN AND ADVERSE PREGNANCY OUTCOMES IN SOUTH ASIAN AND SUB-SAHARAN AFRICAN WOMEN

Contents

COUNTRY-LEVEL ECONOMIC AND HEALTH PROFILE OF THE STUDY REGIONS 6

APPENDIX A – Estimating total gestational weight gain 7

Longitudinal model of gestational weight gain 7

Estimating percentiles of gestational weight gain 8

Table A1 – Percentiles of gestational weight gain for AMANHI-Bangladesh, Underweight BMI (Kg).. 10

Table A2 – Percentiles of gestational weight gain for AMANHI-Bangladesh, Normal BMI (Kg) 11

Table A3 – Percentiles of gestational weight gain for AMANHI-Bangladesh, Overweight BMI (Kg) 12

Table A4 – Percentiles of gestational weight gain for AMANHI-Bangladesh, Obese BMI (Kg) 13

Table A5 – Percentiles of gestational weight gain for AMANHI-Pakistan, Underweight BMI (Kg) 14

Table A6 – Percentiles of gestational weight gain for AMANHI-Pakistan, Normal BMI (Kg) 15

Table A7 – Percentiles of gestational weight gain for AMANHI-Pakistan, Overweight BMI (Kg) 16

Table A8 – Percentiles of gestational weight gain for AMANHI-Pakistan, Obese BMI (Kg) 17

Table A9 – Percentiles of gestational weight gain for PreSSMat, Underweight BMI (Kg)..... 18

Table A10 – Percentiles of gestational weight gain for PreSSMat, Normal BMI (Kg) 19

Table A11 – Percentiles of gestational weight gain for PreSSMat, Overweight BMI (Kg)..... 20

Table A12 – Percentiles of gestational weight gain for PreSSMat, Obese BMI (Kg).............. 21

Table A13 – Percentiles of gestational weight gain for GARBH-Ini, Underweight BMI (Kg) 22

Table A14 – Percentiles of gestational weight gain for GARBH-Ini, Normal BMI (Kg)........ 23

Table A15 – Percentiles of gestational weight gain for GARBH-Ini, Overweight BMI (Kg) . 24

Table A16 – Percentiles of gestational weight gain for GARBH-Ini, Obese BMI (Kg) 25

Table A17 – Percentiles of gestational weight gain for ZAPPS, Underweight BMI (Kg) 26

Table A18 – Percentiles of gestational weight gain for ZAPPS, Normal BMI (Kg)................ 27

Table A19 – Percentiles of gestational weight gain for ZAPPS, Overweight BMI (Kg)......... 28

Table A20 – Percentiles of gestational weight gain for ZAPPS, Obese BMI (Kg) 29

APPENDIX B – Main Text Supplementary Results Tables and Figures 30

TABLES.. 30

Table B1. Study cohort selection flowchart frequencies, overall and by cohort 30

Table B2. Percentage of participants missing data in the overall study population and by cohort 31

Table B3. Distribution of gestational weight gain Z-score[a] by study population characteristics (N= 15286) 32

Table B4. Reference sub-population population characteristics, overall and by study cohort 33

Table B5. Cohort-specific risk ratios for the association between gestational weight gain Z-score and preterm birth (<37 weeks) 35

Table B6. Cohort-specific risk ratios for the association between gestational weight gain Z-score and low birthweight (<2500 Kg) 36

Table B7. Cohort-specific risk ratios for the association between gestational weight gain Z-score and small for gestational age (<10th percentile) 37

Table B8. Cohort-specific risk ratios for the association between gestational weight gain Z-score and small for gestational age (<3rd percentile) 38

Table B9. Risk ratios for the association between gestational weight gain Z-score and adverse outcomes, among participants without previous preterm birth. 39

Figures 41

Figure B1. Observed (circles) and predicted values (curves) for maternal weight gain in the reference sub-population (AMANHI-Bangladesh). 41

Figure B2. Observed (circles) and predicted values (curves) for maternal weight gain in the reference sub-population (AMANHI-Pakistan). 42

Figure B3. Observed (circles) and predicted values (curves) for maternal weight gain in the reference sub-population (PreSSMat). 43

Figure B4. Observed (circles) and predicted values (curves) for maternal weight gain in the reference sub-population (GARBH-Ini). 44

Figure B5. Observed (circles) and predicted values (curves) for maternal weight gain in the reference sub-population (ZAPPS). 45

Figure B6. Non-linear association between probability of preterm birth and GWG z-score, stratified by baseline BMI. 46

Figure B7. Cohort-specific, non-linear association between probability of preterm birth and GWG z-score, stratified by baseline BMI 47

Figure B8. Non-linear association between probability of low birthweight and GWG z-score, stratified by baseline BMI 48

Figure B9. Cohort-specific non-linear association between probability of low birthweight and GWG z-score, in the overall study population and stratified by baseline BMI 49

Figure B10. Non-linear association between probability of SGA (birthweight <10th centile) and GWG z-score, in the overall study population and stratified by baseline BMI. 50

Figure B11. Cohort-specific, non-linear association between probability of SGA (birthweight <10th centile) and GWG z-score, stratified by baseline BMI. 51

Figure B12. Non-linear association between probability of SGA (birthweight <3rd centile) and GWG z-score, stratified by baseline BMI. ... 52

Figure B13. Cohort-specific, non-linear association between probability of SGA (birthweight <3rd centile) and GWG z-score, stratified by baseline BMI. ... 53

APPENDIX C – RESULTS BEFORE MULTIPLE IMPUTATION OF MISSING DATA ... 54

Table C1. Study population characteristics and outcomes, overall and by cohort. Pre-imputation. ... 54

Table C2. Unadjusted risk of primary outcomes by study population characteristics. Pre-imputation. ... 55

Table C3. Risk ratios for the association between gestational weight gain Z-score and preterm birth (<37 weeks), stratified by maternal BMI group. Pre-imputation ... 56

Table C4. Risk ratios for the association between gestational weight gain Z-score and low birthweight (<2500 g), stratified by BMI group. Pre-imputation ... 57

Table C5. Risk ratios for the association between gestational weight gain Z-score and small for gestational age (<10th percentile), stratified by BMI group. Pre-imputation ... 58

Table C6. Risk ratios for the association between gestational weight gain Z-score and small for gestational age (<3rd percentile), stratified by BMI group. Pre-imputation ... 59

Table C7. Study population characteristics and outcomes, overall and by cohort. Pre-imputation. Before exclusion of participants without 2+ valid maternal weight measurements* ... 60

APPENDIX D – USE OF OTHER MEASURES OF GESTATIONAL WEIGHT GAIN IN THE STUDY POPULATION ... 61

1: Institute of Medicine (IOM) gestational weight gain adequacy ratio ... 61

Table D0. Total gestational weight gain in the study population compared to IOM 2009 guidelines. ... 61

Figure D1. IOM weight gain adequacy ratio distribution in the study population (AMANHI-Bangladesh). ... 63

Figure D2. IOM weight gain adequacy ratio distribution in the study population (AMANHI-Pakistan). ... 64

Figure D3. IOM weight gain adequacy ratio distribution in the study population (PreSSMat). ... 65

Figure D4. IOM weight gain adequacy ratio distribution in the study population (GARBH-Ini). ... 66

Figure D5. IOM weight gain adequacy ratio distribution in the study population (ZAPPS). ... 67

Table D1. Quartile group cut-offs of IOM weight gain adequacy ratio (AMANHI-Bangladesh). ... 68

Table D2. Quartile group cut-offs of IOM weight gain adequacy ratio (AMANHI-Pakistan). ... 69

Table D3. Quartile group cut-offs of IOM weight gain adequacy ratio (PreSSMat). 70

Table D4. Quartile group cut-offs of IOM weight gain adequacy ratio (GARBH-Ini). 71

Table D5. BMI- and cohort-specific quartile groups of IOM weight gain adequacy ratio (ZAPPS). 72

Table D6. Risk ratios for the association between IOM gestational weight adequacy ratio (grouped into quartiles) and adverse outcomes (preterm birth, PTB; low birthweight) 73

Table D7. Risk ratios for the association between IOM gestational weight adequacy ratio (grouped into quartiles) and small-for-gestational age (<10th percentile, SGA10; <3rd percentile, SGA3) 74

2. Weight-gain-for-gestational-age Z-scores using INTERGROWTH-21st standards 75

Figure D6. Study observed maternal weight gain (circles) and predicted percentile curves (blue), with overlayed INTERGROWTH-21st percentiles curves (red) in the reference sub-population. Normal BMI participants. 76

Table D8 – Study-specific percentiles of gestational weight gain for AMANHI-Bangladesh vs INTERGROWTH-21st standards, Normal BMI (Kg) 77

Table D9 – Study-specific percentiles of gestational weight gain for AMANHI-Pakistan vs INTERGROWTH-21st standards, Normal BMI (Kg) 78

Table D10 – Study-specific percentiles of gestational weight gain for PreSSMat vs INTERGROWTH-21st standards, Normal BMI (Kg) 79

Table D11 – Study-specific percentiles of gestational weight gain for GARBH-Ini vs INTERGROWTH-21st standards, Normal BMI (Kg) 80

Table D12 – Study-specific percentiles of gestational weight gain for ZAPPS vs INTERGROWTH-21st standards, Normal BMI (Kg) 81

Table D13. Risk ratios for the association between gestational weight gain and adverse outcomes among normal BMI participants. Weight-gain-for-gestational age Z-scores defined using INTERGROWTH-21st (IG-21) weight gain standards. 82

COUNTRY-LEVEL ECONOMIC AND HEALTH PROFILE OF THE STUDY REGIONS

Cohort, region (country)	Human Development Index, Year: 2022	Maternal mortality rate (per 100,000 live births), Year: 2020	Infant mortality rate (per 1,000 live births), Year: 2022
AMANHI-B, Sylhet (Bangladesh)	0.670	123	24
AMANHI-P, Karachi (Pakistan)	0.540	154	51
PreSSMat, Matlab (Bangladesh)	0.670	123	24
ZAPPS, Lusaka (Zambia)	0.569	135	39
GARBH-Ini, Haryana (India)	0.644	103	26

References

1. Human Development Index by country; https://worldpopulationreview.com/country-rankings/hdi-by-country; accessed on August 30, 2024
2. Maternal Mortality Ratio by country; https://data.unicef.org/wp-content/uploads/2023/02/MMR-maternal-deaths-and-LTR_MMEIG-trends_2000-2020_released-Feb_2023.xlsx; accessed on August 30, 2024

Infant Mortality Rate by country; UN-IGME-2023-Child-Mortality-Report.pdf; https://data.unicef.org/wp-content/uploads/2024/04/UN-IGME-2023-Child-Mortality-Report.pdf; accessed on August 30, 2024

APPENDIX A – Estimating total gestational weight gain

Longitudinal model of gestational weight gain

We modeled the outcome of gestational weight gain with gestational age (GA) at follow-up visit as the primary independent variable along with covariates for the main effect of BMI group and the interaction between BMI group and the linear term for gestational age at the visit. The models had a participant level random intercept and a fixed effect for gestational age. To accommodate for non-linearity of effects, the models included restricted quadratic spline terms for gestational age (4 knots at 5p, 35p, 65p, and 95p)(Howe 2011). Original gestational weight gain measurements in kilograms were log-transformed (natural log) to minimize skewness. A constant of +5kg was added to all weight gain measurements to remove negative values before the log transformation.

The random-intercept model for estimating gestational weight gain is as follows:

$$GWG_{ij} = \beta_{0i} + \beta_1 GA_{ij} + \beta_2 BMI1_{ij} + \beta_3 BMI3_{ij} + \beta_4 BM41_{ij} + \beta_5 GA_{ij} BMI1_{ij} + \beta_6 GA_{ij} BMI3 + \beta_7 GA_{ij} BMI4 + \varepsilon_{ij}$$

- Where i represents the ith study participant, and j represents the jth study follow-up visit (at which maternal weight is measured).
- GWG represents the (natural) log-transformed gestational weight gain.
- ε is the within-participant error (residual) at visit j
- GA is the participant's gestational age at visit j, modeled as a restricted quadratic spline
- BMI1, BMI3, BMI4 are indicator variables (1=yes, 0=No) for maternal baseline BMI (underweight, overweight, and obese respectively).
- $\beta_{0i} = \beta_0 + u_i$ is the random effect for the model intercept. We allow each participant to have her own intercept.
- β_1 is the fixed effect of GA for the rate of weight gain.
- $\beta_2, \beta_3, \beta_4,$ are fixed effects for the difference in weight gain associated with baseline underweight, overweight, and obese versus normal BMI respectively.
- $\beta_5, \beta_6, \beta_7,$ are fixed effects for the interaction between GA and a participant's BMI group.
- The following elements are used to estimate the variance of GWG:
    - $Var(\beta_{0i}) = \sigma^2_{\beta_0}$
    - $Var(\varepsilon_{ij}) = \sigma^2_e$
    - $Var(GWG) = \sigma^2_{\beta_0} + \sigma^2_e$

- The resulting mean and variance estimates are shown in the following table:

Estimates from random-intercept linear regression model gestational weight gain.

	AMANHI-Pakistan			AMANHI-Bangladesh			PreSSMat			GARBH-Ini			ZAPPS		
Model fixed effects	Est	LCL	UCL	Est	LCL	UCL	Est	LCL	UCL	Est	LCL	UCL	Est	LCL	UCL
Intercept	1.056	0.196	1.916	1.144	0.958	1.329	0.861	0.728	0.995	0.867	0.746	0.988	1.000	0.306	1.693
Gestational age (GA)	0.042	0.007	0.077	0.035	0.027	0.043	0.053	0.047	0.058	0.050	0.044	0.057	0.045	0.015	0.076
Spline basis function (_GA)[a]	-0.048	-0.171	0.075	-0.006	-0.021	0.010	-0.029	-0.043	-0.015	-0.006	-0.021	0.009	-0.004	-0.108	0.100
Spline basis function (__GA)[a]	0.060	-0.094	0.215	0.001	-0.031	0.033	0.032	0.008	0.056	-0.031	-0.064	0.002	-0.008	-0.133	0.117
Spline basis function (___GA)[a]	-0.023	-0.118	0.072	-0.007	-0.059	0.045	-0.036	-0.054	-0.018	0.029	0.002	0.056	0.030	-0.052	0.111
BMI group indicator (BMI_1)[b]	0.277	0.151	0.402	0.226	0.146	0.305	0.142	0.098	0.187	0.145	0.115	0.175	0.300	0.039	0.561
BMI group indicator (BMI_2)[b]	0.011	-0.122	0.144	-0.100	-0.262	0.062	-0.138	-0.182	-0.093	-0.073	-0.115	-0.031	-0.003	-0.141	0.135
BMI group indicator (BMI_3)[b]	-0.071	-0.271	0.129	-0.089	-0.516	0.338	-0.181	-0.288	-0.075	-0.158	-0.267	-0.048	0.033	-0.132	0.199
Interaction: GA*BMI_1	-0.002	-0.006	0.001	-0.003	-0.005	-0.001	-0.001	-0.003	0.000	-0.002	-0.003	-0.001	-0.003	-0.011	0.005
Interaction: GA*BMI_3	-0.007	-0.011	-0.003	-0.001	-0.006	0.004	0.000	-0.001	0.002	-0.001	-0.002	0.000	-0.005	-0.009	0.000
Interaction: GA*BMI_4	-0.005	-0.011	0.001	-0.004	-0.017	0.009	-0.001	-0.005	0.002	0.001	-0.002	0.004	-0.008	-0.013	-0.003
Variance estimates															
Random-intercept	0.102			0.080			0.055			0.052			0.098		
Random error (residual)	0.061			0.063			0.017			0.028			0.073		

All estimates are shown as ln(kg).
Standard deviation estimates in Kg. AMANHI-Pakistan: 1.50; AMANHI-Bangladesh: 1.46; PreSSMat: 1.31; GARBH-Ini: 1.33; ZAPSS: 1.51
[a]Restricted quadratic splines with 4 knots at 5, 35, 65, 95 percentiles of gestational age, calculated as:
_GA = $(\max(0, GA\text{-}p5)^2 - \max(0, GA\text{-}p95)^2) / (p95\text{-}p5)$;
__GA = $(\max(0, GA\text{-}p35)^2 - \max(0, GA\text{-}p95)^2) / (p95\text{-}p5)$;
___GA = $(\max(0, GA\text{-}p65)^2 - \max(0, GA\text{-}p95)^2) / (p95\text{-}p5)$;

Estimating percentiles of gestational weight gain

Using the model estimates for the mean and standard deviation (SD) of gestational weight gain, we estimated the percentiles of interest based on the formula: $\text{P}^{\text{th}}\text{ percentile} = \text{mean} + ZSD$, where Z is the Z-score that corresponds to the percentile value in the standard normal distribution, for example, Z=0.675 for the 75th percentile. The 75th percentile of gestational weight gain at 37 weeks gestation, among normal BMI participants in the AMANHI-Pakistan cohort would be calculated as follows: $\text{75th percentile} = \exp\big(2.647 + (0.675)(0.404)\big) = 13.5\ kg$. Likewise, the weight-gain-for-gestational-age Z-score was calculated using the formula: $\text{Z} = (weight\ gain - \text{mean})/\text{SD}$. For example, for an AMANHI-Pakistan participant delivering at 37 weeks, with normal BMI and a total gestational weight gain of 10 kg, the weight-gain-for-gestational-age Z-score would be: $(\ln(10 + c) - 2.647)/0.404$ where c is the constant of 5kg, 2.647 is the (natural) log-transformed mean of gestational weight gain for normal weight participants at 37 weeks, and 0.404 is the standard deviation. Overall, our approach has similarities to those used by others (Hutcheon 2013; Ismail 2016), except that: (i) we estimated mean and standard deviation values using our sample rather than published charts, (ii) our estimates of standard deviation come from random-intercept only models, rather than random intercept and slope models, which did not fit our data well.

References

1. Howe CJ, Cole SR, Westreich DJ, Greenland S, Napravnik S, Eron Jr JJ. Splines for trend analysis and continuous confounder control. Epidemiology (Cambridge, Mass.). 2011 Nov;22(6):874.

2. Hutcheon JA, Platt RW, Abrams B, Himes KP, Simhan HN, Bodnar LM. A weight-gain-for-gestational-age z score chart for the assessment of maternal weight gain in pregnancy. The American of Clinical Nutrition. 2013 May 1;97(5):1062-7.

3. Ismail LC, Bishop DC, Pang R, Ohuma EO, Kac G, Abrams B, Rasmussen K, Barros FC, Hirst JE, Lambert A, Papageorghiou AT. Gestational weight gain standards based on women enrolled in the Fetal Growth Longitudinal Study of the INTERGROWTH-21st Project: a prospective longitudinal cohort study. bmj. 2016 Feb 29;352.

Table A1 – Percentiles of gestational weight gain for AMANHI-Bangladesh, Underweight BMI (Kg)

Gestational age, weeks	p10	p25	p50	p75	p90	Observations, n	%	Cumulative n	Cumulative %
18	-0.69	0.42	1.99	4.02	6.35	12	0.6	12	0.6
19	-0.55	0.59	2.22	4.31	6.72	20	1.0	32	1.5
20	-0.41	0.77	2.45	4.62	7.10	34	1.6	66	3.2
21	-0.26	0.96	2.69	4.93	7.49	53	2.5	119	5.7
22	-0.11	1.15	2.94	5.25	7.89	81	3.9	200	9.5
23	0.05	1.35	3.19	5.58	8.30	144	6.9	344	16.4
24	0.20	1.55	3.45	5.90	8.72	129	6.2	473	22.6
25	0.36	1.74	3.70	6.23	9.13	102	4.9	575	27.4
26	0.52	1.94	3.96	6.57	9.55	80	3.8	655	31.2
27	0.68	2.15	4.22	6.90	9.97	58	2.8	713	34.0
28	0.84	2.35	4.48	7.24	10.39	56	2.7	769	36.7
29	1.00	2.55	4.74	7.58	10.82	69	3.3	838	39.9
30	1.16	2.75	5.01	7.92	11.25	101	4.8	939	44.8
31	1.33	2.96	5.27	8.26	11.68	155	7.4	1094	52.1
32	1.49	3.16	5.54	8.60	12.11	161	7.7	1255	59.8
33	1.65	3.37	5.80	8.94	12.54	122	5.8	1377	65.6
34	1.82	3.58	6.07	9.29	12.97	101	4.8	1478	70.5
35	1.98	3.78	6.33	9.62	13.39	102	4.9	1580	75.3
36	2.13	3.97	6.58	9.94	13.79	107	5.1	1687	80.4
37	2.28	4.15	6.81	10.25	14.18	183	8.7	1870	89.1
38	2.42	4.33	7.04	10.54	14.54	152	7.2	2022	96.4
39	2.55	4.50	7.26	10.82	14.90	53	2.5	2075	98.9
40	2.68	4.67	7.48	11.10	15.25	21	1.0	2096	99.9

Table A2 – Percentiles of gestational weight gain for AMANHI-Bangladesh, Normal BMI (Kg)

Gestational age, weeks	p10	p25	p50	p75	p90	Observations, n	%	Cumulative n	Cumulative %
18	-1.37	-0.44	0.89	2.60	4.56	14	0.4	14	0.4
19	-1.25	-0.28	1.10	2.87	4.90	28	0.7	42	1.1
20	-1.11	-0.11	1.31	3.15	5.25	48	1.2	90	2.3
21	-0.97	0.07	1.54	3.44	5.61	65	1.6	155	3.9
22	-0.83	0.24	1.77	3.74	5.99	129	3.3	284	7.2
23	-0.69	0.43	2.00	4.04	6.37	304	7.7	588	14.8
24	-0.54	0.61	2.24	4.35	6.76	286	7.2	874	22.0
25	-0.39	0.80	2.49	4.66	7.15	188	4.7	1062	26.8
26	-0.24	0.99	2.73	4.98	7.55	140	3.5	1202	30.3
27	-0.09	1.18	2.98	5.30	7.95	88	2.2	1290	32.5
28	0.07	1.38	3.23	5.62	8.36	97	2.5	1387	35.0
29	0.22	1.57	3.48	5.94	8.77	114	2.9	1501	37.8
30	0.38	1.77	3.74	6.27	9.18	179	4.5	1680	42.4
31	0.54	1.97	3.99	6.61	9.60	349	8.8	2029	51.2
32	0.70	2.17	4.25	6.94	10.02	308	7.8	2337	58.9
33	0.86	2.37	4.52	7.28	10.45	231	5.8	2568	64.7
34	1.02	2.58	4.78	7.62	10.88	172	4.3	2740	69.1
35	1.18	2.78	5.04	7.95	11.30	146	3.7	2886	72.8
36	1.34	2.97	5.29	8.28	11.70	226	5.7	3112	78.5
37	1.49	3.16	5.53	8.59	12.10	393	9.9	3505	88.4
38	1.63	3.34	5.76	8.89	12.47	301	7.6	3806	95.9
39	1.77	3.52	5.99	9.18	12.84	120	3.0	3926	99.0
40	1.91	3.69	6.22	9.48	13.22	37	0.9	3963	99.9

Table A3 – Percentiles of gestational weight gain for AMANHI-Bangladesh, Overweight BMI (Kg)

Gestational age, weeks	p10	p25	p50	p75	p90	Observations, n	%	Cumulative n	Cumulative %
18	-1.78	-0.95	0.23	1.75	3.49	2	0.6	2	0.6
19	-1.67	-0.81	0.41	1.98	3.78	1	0.3	3	0.9
20	-1.55	-0.66	0.60	2.22	4.09	2	0.6	5	1.4
21	-1.43	-0.51	0.79	2.47	4.40	7	2.0	12	3.4
22	-1.31	-0.36	0.99	2.73	4.72	14	4.0	26	7.4
23	-1.19	-0.20	1.19	2.99	5.05	27	7.7	53	15.0
24	-1.06	-0.04	1.40	3.26	5.38	24	6.8	77	21.8
25	-0.93	0.12	1.60	3.52	5.72	25	7.1	102	28.9
26	-0.80	0.28	1.81	3.79	6.06	7	2.0	109	30.9
27	-0.67	0.44	2.02	4.06	6.40	11	3.1	120	34.0
28	-0.54	0.61	2.24	4.34	6.75	6	1.7	126	35.7
29	-0.41	0.77	2.45	4.62	7.10	5	1.4	131	37.1
30	-0.28	0.94	2.67	4.89	7.45	18	5.1	149	42.2
31	-0.14	1.11	2.88	5.18	7.80	26	7.4	175	49.6
32	-0.01	1.28	3.10	5.46	8.16	29	8.2	204	57.8
33	0.13	1.45	3.33	5.75	8.52	14	4.0	218	61.8
34	0.27	1.62	3.55	6.03	8.88	20	5.7	238	67.4
35	0.40	1.79	3.76	6.31	9.23	10	2.8	248	70.3
36	0.53	1.95	3.97	6.58	9.57	23	6.5	271	76.8
37	0.65	2.11	4.18	6.84	9.90	37	10.5	308	87.3
38	0.77	2.26	4.37	7.09	10.21	28	7.9	336	95.2
39	0.89	2.41	4.56	7.34	10.52	17	4.8	353	100.0

Table A4 – Percentiles of gestational weight gain for AMANHI-Bangladesh, Obese BMI (Kg)

Gestational age, weeks	p10	p25	p50	p75	p90	Observations, n	%	Cumulative n	Cumulative %
20	-1.70	-0.85	0.36	1.91	3.69	1	1.9	1	1.9
23	-1.38	-0.45	0.88	2.58	4.54	6	11.3	7	13.2
24	-1.27	-0.31	1.05	2.81	4.83	5	9.4	12	22.6
25	-1.16	-0.17	1.23	3.04	5.12	1	1.9	13	24.5
26	-1.05	-0.03	1.41	3.27	5.41	2	3.8	15	28.3
27	-0.94	0.11	1.59	3.51	5.70	1	1.9	16	30.2
29	-0.72	0.39	1.95	3.97	6.29	2	3.8	18	34.0
30	-0.60	0.53	2.14	4.21	6.58	1	1.9	19	35.9
31	-0.49	0.67	2.32	4.45	6.88	3	5.7	22	41.5
32	-0.38	0.81	2.50	4.68	7.18	5	9.4	27	50.9
33	-0.27	0.95	2.69	4.92	7.48	5	9.4	32	60.4
34	-0.15	1.10	2.87	5.16	7.77	1	1.9	33	62.3
35	-0.04	1.23	3.05	5.38	8.06	2	3.8	35	66.0
36	0.06	1.37	3.22	5.60	8.34	3	5.7	38	71.7
37	0.16	1.49	3.38	5.81	8.60	9	17.0	47	88.7
38	0.25	1.61	3.53	6.01	8.85	5	9.4	52	98.1
39	0.34	1.72	3.68	6.20	9.09	1	1.9	53	100.0

Table A5 – Percentiles of gestational weight gain for AMANHI-Pakistan, Underweight BMI (Kg)

Gestational age, weeks	p10	p25	p50	p75	p90	Observations, n	%	Cumulative n	Cumulative %
21	0.32	1.80	3.93	6.73	9.99	1	0.1	1	0.1
23	0.64	2.21	4.47	7.43	10.88	2	0.2	3	0.4
24	0.81	2.42	4.74	7.79	11.35	134	15.5	137	15.9
25	0.97	2.63	5.02	8.16	11.82	144	16.7	281	32.6
26	1.14	2.84	5.30	8.52	12.28	19	2.2	300	34.8
27	1.30	3.04	5.56	8.87	12.72	6	0.7	306	35.5
28	1.46	3.25	5.84	9.23	13.18	4	0.5	310	35.9
29	1.63	3.48	6.13	9.62	13.68	4	0.5	314	36.4
30	1.82	3.72	6.45	10.03	14.21	2	0.2	316	36.6
31	2.03	3.98	6.79	10.48	14.78	6	0.7	322	37.3
32	2.24	4.26	7.16	10.96	15.39	131	15.2	453	52.5
33	2.48	4.56	7.55	11.48	16.06	175	20.3	628	72.8
34	2.72	4.86	7.95	12.01	16.74	14	1.6	642	74.4
35	2.96	5.17	8.35	12.53	17.40	6	0.7	648	75.1
36	3.19	5.46	8.74	13.04	18.05	2	0.2	650	75.3
37	3.41	5.75	9.11	13.53	18.68	7	0.8	657	76.1
38	3.63	6.02	9.48	14.01	19.29	105	12.2	762	88.3
39	3.84	6.29	9.83	14.47	19.88	95	11.0	857	99.3
40	4.04	6.55	10.17	14.93	20.46	6	0.7	863	100.0

Table A6 – Percentiles of gestational weight gain for AMANHI-Pakistan, Normal BMI (Kg)

Gestational age, weeks	p10	p25	p50	p75	p90	Observations, n	%	Cumulative n	Cumulative %
19	-0.89	0.26	1.90	4.06	6.58	1	0.1	1	0.1
21	-0.64	0.58	2.32	4.62	7.29	1	0.1	2	0.1
22	-0.50	0.74	2.54	4.91	7.66	1	0.1	3	0.1
23	-0.37	0.92	2.77	5.21	8.04	5	0.2	8	0.4
24	-0.23	1.10	3.01	5.51	8.43	297	13.9	305	14.2
25	-0.09	1.28	3.24	5.82	8.83	380	17.7	685	31.9
26	0.05	1.45	3.48	6.13	9.22	52	2.4	737	34.4
27	0.19	1.63	3.70	6.43	9.60	20	0.9	757	35.3
28	0.33	1.80	3.93	6.73	9.99	17	0.8	774	36.1
29	0.47	1.99	4.19	7.06	10.41	10	0.5	784	36.6
30	0.64	2.20	4.46	7.42	10.86	6	0.3	790	36.8
31	0.81	2.42	4.75	7.80	11.35	8	0.4	798	37.2
32	0.99	2.66	5.06	8.21	11.87	317	14.8	1115	52.0
33	1.19	2.91	5.39	8.65	12.44	410	19.1	1525	71.1
34	1.40	3.18	5.74	9.10	13.01	60	2.8	1585	73.9
35	1.60	3.43	6.07	9.54	13.58	20	0.9	1605	74.8
36	1.80	3.68	6.41	9.98	14.14	5	0.2	1610	75.1
37	1.99	3.93	6.73	10.40	14.68	8	0.4	1618	75.4
38	2.18	4.17	7.04	10.81	15.20	279	13.0	1897	88.4
39	2.35	4.40	7.34	11.20	15.70	236	11.0	2133	99.4
40	2.53	4.63	7.64	11.60	16.21	12	0.6	2145	100.0

Table A7 – Percentiles of gestational weight gain for AMANHI-Pakistan, Overweight BMI (Kg)

Gestational age, weeks	p10	p25	p50	p75	p90	Observations, n	%	Cumulative n	Cumulative %
23	-0.98	0.14	1.74	3.86	6.32	2	0.3	2	0.3
24	-0.89	0.25	1.90	4.06	6.57	76	10.1	78	10.3
25	-0.80	0.37	2.05	4.26	6.83	150	19.9	228	30.2
26	-0.71	0.48	2.20	4.45	7.08	27	3.6	255	33.8
27	-0.63	0.59	2.34	4.63	7.31	11	1.5	266	35.3
28	-0.54	0.69	2.48	4.82	7.55	4	0.5	270	35.8
29	-0.45	0.81	2.63	5.02	7.81	2	0.3	272	36.1
30	-0.35	0.94	2.80	5.24	8.09	2	0.3	274	36.3
31	-0.24	1.08	2.98	5.48	8.39	4	0.5	278	36.9
32	-0.13	1.23	3.18	5.74	8.72	83	11.0	361	47.9
33	0.00	1.39	3.39	6.01	9.07	161	21.4	522	69.2
34	0.13	1.55	3.60	6.30	9.43	27	3.6	549	72.8
35	0.25	1.71	3.81	6.57	9.78	5	0.7	554	73.5
36	0.37	1.86	4.01	6.83	10.11	2	0.3	556	73.7
37	0.48	2.00	4.19	7.07	10.43	4	0.5	560	74.3
38	0.58	2.13	4.37	7.30	10.72	80	10.6	640	84.9
39	0.68	2.26	4.53	7.52	11.00	109	14.5	749	99.3
40	0.78	2.38	4.69	7.73	11.27	4	0.5	753	99.9

Table A8 – Percentiles of gestational weight gain for AMANHI-Pakistan, Obese BMI (Kg)

Gestational age, weeks	p10	p25	p50	p75	p90	Observations, n	%	Cumulative n	Cumulative %
24	-1.10	-0.01	1.55	3.60	5.99	29	10.4	29	10.4
25	-0.99	0.12	1.73	3.83	6.29	55	19.6	84	30.0
26	-0.89	0.26	1.90	4.06	6.58	8	2.9	92	32.9
27	-0.79	0.38	2.07	4.29	6.86	2	0.7	94	33.6
28	-0.68	0.52	2.24	4.51	7.15	3	1.1	97	34.6
29	-0.57	0.66	2.43	4.76	7.47	1	0.4	98	35.0
30	-0.45	0.81	2.63	5.02	7.80	2	0.7	100	35.7
31	-0.32	0.98	2.85	5.30	8.17	2	0.7	102	36.4
32	-0.18	1.15	3.08	5.61	8.56	39	13.9	141	50.4
33	-0.04	1.34	3.33	5.94	8.98	54	19.3	195	69.6
34	0.12	1.54	3.59	6.28	9.41	8	2.9	203	72.5
37	0.55	2.09	4.32	7.23	10.63	2	0.7	205	73.2
38	0.69	2.27	4.54	7.53	11.01	33	11.8	238	85.0
39	0.82	2.43	4.76	7.82	11.37	39	13.9	277	98.9
40	0.94	2.59	4.97	8.10	11.74	3	1.1	280	100.0

Table A9 – Percentiles of gestational weight gain for PreSSMat, Underweight BMI (Kg)

Gestational age, weeks	p10	p25	p50	p75	p90	Observations, n	%	Cumulative n	Cumulative %
19	0.12	1.03	2.22	3.66	5.20	1	0.1	1	0.1
21	0.67	1.68	3.00	4.60	6.30	32	1.8	33	1.9
22	0.97	2.03	3.42	5.10	6.89	204	11.7	237	13.6
23	1.27	2.38	3.85	5.61	7.49	154	8.8	391	22.4
24	1.56	2.73	4.26	6.11	8.08	59	3.4	450	25.8
25	1.85	3.06	4.67	6.59	8.64	17	1.0	467	26.7
26	2.12	3.38	5.05	7.05	9.19	13	0.7	480	27.5
27	2.38	3.69	5.42	7.49	9.71	108	6.2	588	33.7
28	2.63	3.98	5.77	7.91	10.20	128	7.3	716	41.0
29	2.88	4.27	6.12	8.33	10.69	62	3.6	778	44.5
30	3.14	4.58	6.49	8.77	11.21	24	1.4	802	45.9
31	3.41	4.90	6.87	9.23	11.75	126	7.2	928	53.1
32	3.69	5.23	7.27	9.71	12.32	201	11.5	1129	64.6
33	3.98	5.58	7.68	10.20	12.90	100	5.7	1229	70.4
34	4.26	5.90	8.07	10.67	13.44	27	1.6	1256	71.9
35	4.50	6.19	8.41	11.08	13.93	85	4.9	1341	76.8
36	4.72	6.44	8.72	11.45	14.36	133	7.6	1474	84.4
37	4.90	6.66	8.98	11.76	14.73	82	4.7	1556	89.1
38	5.05	6.83	9.19	12.01	15.02	47	2.7	1603	91.8
39	5.16	6.96	9.34	12.20	15.25	61	3.5	1664	95.3
40	5.24	7.06	9.46	12.34	15.41	61	3.5	1725	98.7

Table A10 – Percentiles of gestational weight gain for PreSSMat, Normal BMI (Kg)

Gestational age, weeks	p10	p25	p50	p75	p90	Observations, n	%	Cumulative n	Cumulative %
19	-0.44	0.36	1.43	2.71	4.07	1	0.0	1	0.0
21	0.06	0.96	2.14	3.56	5.08	95	1.4	96	1.4
22	0.33	1.28	2.53	4.03	5.63	774	11.1	870	12.5
23	0.61	1.60	2.92	4.49	6.18	626	9.0	1496	21.5
24	0.88	1.92	3.30	4.95	6.72	274	3.9	1770	25.5
25	1.14	2.23	3.67	5.40	7.24	107	1.5	1877	27.0
26	1.40	2.53	4.03	5.83	7.75	61	0.9	1938	27.9
27	1.64	2.82	4.37	6.24	8.23	363	5.2	2301	33.1
28	1.87	3.09	4.70	6.63	8.69	536	7.7	2837	40.8
29	2.11	3.37	5.03	7.03	9.16	239	3.4	3076	44.2
30	2.35	3.65	5.38	7.44	9.64	103	1.5	3179	45.7
31	2.61	3.95	5.74	7.87	10.15	480	6.9	3659	52.6
32	2.87	4.27	6.11	8.32	10.68	771	11.1	4430	63.7
33	3.15	4.59	6.50	8.79	11.23	409	5.9	4839	69.6
34	3.41	4.90	6.87	9.23	11.75	101	1.5	4940	71.0
35	3.64	5.17	7.20	9.63	12.22	315	4.5	5255	75.6
36	3.85	5.42	7.49	9.98	12.63	584	8.4	5839	84.0
37	4.03	5.63	7.75	10.28	12.99	365	5.3	6204	89.2
38	4.18	5.80	7.95	10.53	13.28	171	2.5	6375	91.7
39	4.29	5.94	8.12	10.73	13.51	252	3.6	6627	95.3
40	4.38	6.04	8.24	10.87	13.68	217	3.1	6844	98.4

Table A11 – Percentiles of gestational weight gain for PreSSMat, Overweight BMI (Kg)

Gestational age, weeks	p10	p25	p50	p75	p90	Observations, n	%	Cumulative n	Cumulative %
20	-0.79	-0.04	0.95	2.13	3.39	1	0.1	1	0.1
21	-0.56	0.23	1.27	2.52	3.85	24	1.3	25	1.4
22	-0.32	0.51	1.61	2.93	4.33	210	11.6	235	13.0
23	-0.07	0.80	1.96	3.34	4.82	172	9.5	407	22.6
24	0.17	1.08	2.29	3.75	5.30	63	3.5	470	26.1
25	0.40	1.36	2.62	4.14	5.76	23	1.3	493	27.3
26	0.63	1.62	2.94	4.52	6.21	18	1.0	511	28.3
27	0.84	1.88	3.25	4.89	6.64	94	5.2	605	33.5
28	1.05	2.12	3.54	5.23	7.05	125	6.9	730	40.5
29	1.26	2.36	3.83	5.59	7.46	81	4.5	811	45.0
30	1.47	2.62	4.14	5.95	7.90	25	1.4	836	46.3
31	1.70	2.89	4.46	6.34	8.35	158	8.8	994	55.1
32	1.94	3.17	4.79	6.74	8.82	192	10.6	1186	65.7
33	2.18	3.46	5.14	7.16	9.31	99	5.5	1285	71.2
34	2.41	3.73	5.47	7.55	9.77	30	1.7	1315	72.9
35	2.62	3.98	5.76	7.90	10.19	87	4.8	1402	77.7
36	2.81	4.20	6.02	8.22	10.56	146	8.1	1548	85.8
37	2.97	4.38	6.25	8.49	10.88	76	4.2	1624	90.0
38	3.10	4.54	6.44	8.72	11.15	56	3.1	1680	93.1
39	3.21	4.66	6.59	8.89	11.35	58	3.2	1738	96.3
40	3.29	4.76	6.70	9.03	11.51	42	2.3	1780	98.7

Table A12 – Percentiles of gestational weight gain for PreSSMat, Obese BMI (Kg)

Gestational age, weeks	p10	p25	p50	p75	p90	Observations, n	%	Cumulative n	Cumulative %
21	-0.91	-0.18	0.78	1.93	3.15	5	2.1	5	2.1
22	-0.69	0.07	1.08	2.29	3.58	38	15.8	43	17.9
23	-0.48	0.33	1.39	2.66	4.01	19	7.9	62	25.8
24	-0.26	0.58	1.68	3.01	4.43	6	2.5	68	28.3
25	-0.06	0.82	1.97	3.36	4.84	3	1.3	71	29.6
26	0.14	1.05	2.25	3.69	5.24	1	0.4	72	30.0
27	0.32	1.27	2.52	4.01	5.61	16	6.7	88	36.7
28	0.50	1.48	2.77	4.31	5.96	16	6.7	104	43.3
29	0.68	1.69	3.02	4.61	6.32	8	3.3	112	46.7
30	0.87	1.91	3.28	4.93	6.69	2	0.8	114	47.5
31	1.06	2.14	3.56	5.26	7.08	18	7.5	132	55.0
32	1.27	2.38	3.84	5.60	7.48	24	10.0	156	65.0
33	1.48	2.62	4.14	5.96	7.90	18	7.5	174	72.5
34	1.67	2.85	4.42	6.29	8.29	3	1.3	177	73.8
35	1.85	3.06	4.67	6.59	8.64	12	5.0	189	78.8
36	2.00	3.24	4.88	6.85	8.95	14	5.8	203	84.6
37	2.13	3.40	5.07	7.07	9.21	10	4.2	213	88.8
38	2.24	3.52	5.22	7.25	9.42	8	3.3	221	92.1
39	2.32	3.62	5.33	7.39	9.58	10	4.2	231	96.3
40	2.38	3.68	5.41	7.48	9.70	5	2.1	236	98.3

Table A13 – Percentiles of gestational weight gain for GARBH-Ini, Underweight BMI (Kg)

Gestational age, weeks	p10	p25	p50	p75	p90	Observations, n	%	Cumulative n	Cumulative %
18	-0.44	0.41	1.56	2.94	4.43	453	10.8	453	10.8
19	-0.22	0.68	1.88	3.33	4.90	515	12.2	968	23.0
20	0.01	0.96	2.21	3.74	5.38	99	2.4	1067	25.3
21	0.25	1.24	2.56	4.16	5.88	16	0.4	1083	25.7
23	0.76	1.84	3.29	5.04	6.92	1	0.0	1084	25.7
25	1.30	2.49	4.07	5.98	8.04	1	0.0	1085	25.8
26	1.59	2.82	4.48	6.48	8.63	637	15.1	1722	40.9
27	1.88	3.17	4.89	6.98	9.23	289	6.9	2011	47.7
28	2.15	3.50	5.29	7.46	9.80	83	2.0	2094	49.7
29	2.42	3.81	5.67	7.92	10.35	19	0.5	2113	50.2
30	2.66	4.10	6.03	8.35	10.86	88	2.1	2201	52.2
31	2.89	4.38	6.36	8.75	11.34	796	18.9	2997	71.1
32	3.12	4.64	6.68	9.14	11.80	113	2.7	3110	73.8
33	3.34	4.90	6.99	9.53	12.26	28	0.7	3138	74.5
34	3.56	5.17	7.31	9.91	12.71	1	0.0	3139	74.5
35	3.78	5.43	7.63	10.29	13.17	177	4.2	3316	78.7
36	4.00	5.69	7.94	10.68	13.62	116	2.8	3432	81.5
37	4.22	5.95	8.26	11.05	14.07	50	1.2	3482	82.7
38	4.43	6.20	8.57	11.43	14.52	157	3.7	3639	86.4
39	4.64	6.45	8.87	11.80	14.96	245	5.8	3884	92.2
40	4.85	6.70	9.17	12.16	15.39	224	5.3	4108	97.5

Table A14 – Percentiles of gestational weight gain for GARBH-Ini, Normal BMI (Kg)

Gestational age, weeks	p10	p25	p50	p75	p90	Observations, n	%	Cumulative n	Cumulative %
18	-0.93	-0.16	0.86	2.09	3.43	1086	10.7	1086	10.7
19	-0.72	0.08	1.16	2.46	3.86	1168	11.5	2254	22.1
20	-0.50	0.34	1.47	2.83	4.30	213	2.1	2467	24.2
21	-0.28	0.61	1.79	3.22	4.77	20	0.2	2487	24.4
26	0.97	2.09	3.59	5.40	7.36	1624	16.0	4111	40.4
27	1.24	2.42	3.98	5.88	7.92	674	6.6	4785	47.0
28	1.51	2.73	4.36	6.33	8.46	162	1.6	4947	48.6
29	1.76	3.03	4.72	6.77	8.99	54	0.5	5001	49.1
30	2.00	3.31	5.06	7.19	9.48	238	2.3	5239	51.5
31	2.22	3.57	5.38	7.58	9.94	2005	19.7	7244	71.2
32	2.43	3.83	5.70	7.95	10.39	243	2.4	7487	73.6
33	2.65	4.09	6.01	8.33	10.84	50	0.5	7537	74.0
35	3.09	4.61	6.63	9.09	11.74	465	4.6	8002	78.6
36	3.30	4.86	6.94	9.47	12.18	265	2.6	8267	81.2
37	3.52	5.12	7.25	9.84	12.63	124	1.2	8391	82.4
38	3.73	5.37	7.56	10.21	13.07	325	3.2	8716	85.6
39	3.95	5.63	7.87	10.59	13.51	532	5.2	9248	90.8
40	4.16	5.88	8.17	10.95	13.95	603	5.9	9851	96.8

Table A15 – Percentiles of gestational weight gain for GARBH-Ini, Overweight BMI (Kg)

Gestational age, weeks	p10	p25	p50	p75	p90	Observations, n	%	Cumulative n	Cumulative %
18	-1.29	-0.59	0.34	1.46	2.68	167	9.4	167	9.4
19	-1.11	-0.38	0.60	1.78	3.06	225	12.7	392	22.2
20	-0.91	-0.15	0.88	2.12	3.45	27	1.5	419	23.7
21	-0.72	0.09	1.16	2.46	3.87	4	0.2	423	23.9
26	0.39	1.40	2.75	4.39	6.15	264	14.9	687	38.8
27	0.63	1.68	3.10	4.80	6.65	124	7.0	811	45.9
28	0.86	1.96	3.43	5.21	7.12	38	2.2	849	48.0
29	1.08	2.22	3.74	5.59	7.58	12	0.7	861	48.7
30	1.28	2.47	4.04	5.95	8.01	40	2.3	901	50.9
31	1.48	2.69	4.32	6.28	8.41	352	19.9	1253	70.8
32	1.66	2.92	4.59	6.61	8.79	42	2.4	1295	73.2
33	1.85	3.14	4.86	6.94	9.18	7	0.4	1302	73.6
35	2.22	3.58	5.39	7.58	9.95	75	4.2	1377	77.8
36	2.41	3.80	5.66	7.91	10.33	55	3.1	1432	81.0
37	2.59	4.02	5.92	8.23	10.71	22	1.2	1454	82.2
38	2.77	4.24	6.18	8.54	11.09	68	3.8	1522	86.0
39	2.95	4.45	6.44	8.86	11.46	104	5.9	1626	91.9
40	3.13	4.66	6.70	9.17	11.83	97	5.5	1723	97.4
41	3.31	4.87	6.96	9.48	12.20	36	2.0	1759	99.4
42	3.49	5.09	7.22	9.80	12.58	10	0.6	1769	100.0

Table A16 – Percentiles of gestational weight gain for GARBH-Ini, Obese BMI (Kg)

Gestational age, weeks	p10	p25	p50	p75	p90	Observations, n	%	Cumulative n	Cumulative %
18	-1.47	-0.81	0.08	1.15	2.30	23	9.0	23	9.0
19	-1.29	-0.59	0.34	1.47	2.68	21	8.2	44	17.3
20	-1.10	-0.36	0.61	1.80	3.08	6	2.4	50	19.6
21	-0.90	-0.13	0.90	2.14	3.49	1	0.4	51	20.0
26	0.21	1.19	2.49	4.07	5.78	41	16.1	92	36.1
27	0.45	1.47	2.84	4.49	6.28	24	9.4	116	45.5
28	0.68	1.75	3.18	4.90	6.76	1	0.4	117	45.9
29	0.91	2.02	3.50	5.30	7.23	1	0.4	118	46.3
30	1.12	2.27	3.81	5.67	7.67	2	0.8	120	47.1
31	1.32	2.51	4.10	6.01	8.08	52	20.4	172	67.5
32	1.52	2.74	4.38	6.35	8.49	5	2.0	177	69.4
35	2.11	3.44	5.22	7.38	9.71	16	6.3	193	75.7
36	2.30	3.67	5.50	7.72	10.11	12	4.7	205	80.4
37	2.50	3.91	5.79	8.06	10.52	8	3.1	213	83.5
38	2.69	4.14	6.07	8.40	10.92	14	5.5	227	89.0
39	2.89	4.37	6.34	8.74	11.32	9	3.5	236	92.6
40	3.08	4.60	6.62	9.07	11.72	12	4.7	248	97.3

Table A17 – Percentiles of gestational weight gain for ZAPPS, Underweight BMI (Kg)

Gestational age, weeks	p10	p25	p50	p75	p90	Observations, n	%	Cumulative n	Cumulative %
20	0.05	1.48	3.57	6.32	9.54	1	0.7	1	0.7
21	0.26	1.76	3.94	6.81	10.17	1	0.7	2	1.3
22	0.49	2.06	4.32	7.32	10.83	25	16.3	27	17.7
23	0.73	2.36	4.73	7.85	11.51	14	9.2	41	26.8
24	0.97	2.67	5.14	8.40	12.21	12	7.8	53	34.6
25	1.22	2.99	5.56	8.96	12.94	12	7.8	65	42.5
27	1.73	3.64	6.42	10.10	14.40	3	2.0	68	44.4
28	1.98	3.97	6.85	10.66	15.12	7	4.6	75	49.0
29	2.23	4.29	7.27	11.22	15.84	4	2.6	79	51.6
31	2.72	4.92	8.11	12.32	17.25	2	1.3	81	52.9
32	2.96	5.22	8.51	12.85	17.94	23	15.0	104	68.0
33	3.19	5.53	8.91	13.39	18.62	10	6.5	114	74.5
34	3.46	5.87	9.36	13.97	19.38	2	1.3	116	75.8
35	3.75	6.24	9.85	14.63	20.22	2	1.3	118	77.1
36	4.07	6.65	10.40	15.35	21.15	25	16.3	143	93.5
37	4.43	7.11	11.01	16.15	22.18	8	5.2	151	98.7
39	5.20	8.11	12.32	17.89	24.41	2	1.3	153	100.0

Table A18 – Percentiles of gestational weight gain for ZAPPS, Normal BMI (Kg)

Gestational age, weeks	p10	p25	p50	p75	p90	Observations, n	%	Cumulative n	Cumulative %
19	-1.21	-0.13	1.44	3.51	5.93	1	0.1	1	0.1
20	-1.03	0.10	1.74	3.90	6.44	1	0.1	2	0.1
21	-0.85	0.33	2.05	4.32	6.97	30	1.9	32	2.0
22	-0.66	0.58	2.38	4.75	7.52	224	13.9	256	15.9
23	-0.46	0.84	2.72	5.20	8.10	140	8.7	396	24.6
24	-0.25	1.11	3.07	5.66	8.70	133	8.3	529	32.9
25	-0.03	1.38	3.43	6.14	9.32	81	5.0	610	37.9
26	0.18	1.66	3.80	6.63	9.94	24	1.5	634	39.4
27	0.40	1.94	4.17	7.12	10.57	18	1.1	652	40.6
28	0.62	2.22	4.54	7.61	11.20	101	6.3	753	46.8
29	0.84	2.50	4.91	8.10	11.83	49	3.1	802	49.9
30	1.06	2.78	5.28	8.59	12.46	6	0.4	808	50.3
31	1.27	3.06	5.65	9.07	13.08	32	2.0	840	52.2
32	1.48	3.33	6.01	9.55	13.69	239	14.9	1079	67.1
33	1.70	3.61	6.37	10.03	14.31	117	7.3	1196	74.4
34	1.93	3.91	6.77	10.55	14.98	15	0.9	1211	75.3
35	2.19	4.24	7.21	11.14	15.73	31	1.9	1242	77.2
36	2.48	4.61	7.70	11.78	16.56	232	14.4	1474	91.7
37	2.80	5.02	8.24	12.50	17.48	122	7.6	1596	99.3
38	3.14	5.45	8.81	13.25	18.45	10	0.6	1606	99.9
39	3.49	5.91	9.41	14.05	19.47	2	0.1	1608	100.0

Table A19 – Percentiles of gestational weight gain for ZAPPS, Overweight BMI (Kg)

Gestational age, weeks	p10	p25	p50	p75	p90	Observations, n	%	Cumulative n	Cumulative %
20	-1.40	-0.37	1.12	3.08	5.39	1	0.13	1	0.13
21	-1.25	-0.18	1.37	3.42	5.82	15	1.92	16	2.05
22	-1.09	0.02	1.64	3.77	6.27	92	11.79	108	13.85
23	-0.93	0.23	1.91	4.13	6.73	81	10.38	189	24.23
24	-0.77	0.44	2.19	4.50	7.21	51	6.54	240	30.77
25	-0.59	0.66	2.48	4.88	7.70	46	5.90	286	36.67
26	-0.42	0.88	2.77	5.27	8.19	11	1.41	297	38.08
27	-0.25	1.10	3.06	5.65	8.68	12	1.54	309	39.62
28	-0.08	1.32	3.35	6.03	9.17	49	6.28	358	45.90
29	0.08	1.53	3.63	6.40	9.65	28	3.59	386	49.49
30	0.25	1.74	3.91	6.77	10.13	6	0.77	392	50.26
31	0.41	1.95	4.18	7.14	10.59	14	1.79	406	52.05
32	0.57	2.15	4.45	7.49	11.05	99	12.69	505	64.74
33	0.72	2.35	4.72	7.84	11.50	77	9.87	582	74.62
34	0.90	2.57	5.01	8.23	11.99	7	0.90	589	75.51
35	1.09	2.82	5.34	8.66	12.55	20	2.56	609	78.08
36	1.30	3.10	5.70	9.14	13.17	92	11.79	701	89.87
37	1.54	3.40	6.10	9.67	13.85	69	8.85	770	98.72
38	1.79	3.72	6.53	10.23	14.57	7	0.90	777	99.62
39	2.05	4.06	6.97	10.82	15.33	1	0.13	778	99.74

Table A20 – Percentiles of gestational weight gain for ZAPPS, Obese BMI (Kg)

Gestational age, weeks	p10	p25	p50	p75	p90	Observations, n	%	Cumulative n	Cumulative %
21	-1.36	-0.32	1.18	3.17	5.49	7	1.48	7	1.48
22	-1.22	-0.14	1.42	3.48	5.90	72	15.19	79	16.67
23	-1.08	0.04	1.66	3.80	6.31	34	7.17	113	23.84
24	-0.93	0.23	1.91	4.13	6.73	35	7.38	148	31.22
25	-0.78	0.42	2.16	4.47	7.16	30	6.33	178	37.55
26	-0.63	0.61	2.42	4.80	7.60	6	1.27	184	38.82
27	-0.48	0.81	2.67	5.14	8.03	3	0.63	187	39.45
28	-0.34	0.99	2.92	5.47	8.45	29	6.12	216	45.57
29	-0.19	1.18	3.16	5.79	8.86	15	3.16	231	48.73
31	0.08	1.53	3.63	6.41	9.66	9	1.90	240	50.63
32	0.22	1.70	3.86	6.70	10.03	73	15.40	313	66.03
33	0.35	1.87	4.08	6.99	10.41	39	8.23	352	74.26
34	0.49	2.05	4.32	7.32	10.82	3	0.63	355	74.89
35	0.65	2.26	4.59	7.68	11.29	10	2.11	365	77.00
36	0.83	2.49	4.90	8.08	11.81	69	14.56	434	91.56
37	1.03	2.75	5.24	8.53	12.39	35	7.38	469	98.95
38	1.24	3.02	5.60	9.01	13.00	5	1.05	474	100.00

APPENDIX B – Main Text Supplementary Results Tables and Figures

TABLES

Table B1. Study cohort selection flowchart frequencies, overall and by cohort

Node	Overall	AMANHI-Bangladesh	AMANHI-Pakistan	PreSSMat	GARBH-Ini	ZAPPS
MOMI<br>N = 17,330 unique participants[a]	17,330	2,920	2,463	3,582	7,165	1,200
Exclusion arrows: Missing baseline height, Missing gestational weight gain[b]	No height: 232, No GWG: 1565 (TOTAL: 1797; 10.3%)	No height: 10, No GWG: 87 (TOTAL: 97; 3.3%)	No height: 154, No GWG: 269 (TOTAL: 423; 17.1%)	No height: 0 , No GWG: 93 (TOTAL: 93; 2.6%)	No height: 4, No GWG: 1028 (TOTAL: 1032; 14.4%)	No height: 64, No GWG: 88 (TOTAL: 152; 12.6%)
Available height & gestational weight gain	15,533	2,823	2,040	3,489	6,133	1,048
Exclusion arrows: Abortion, Missing neonatal vital status, Twin pregnancy/multiples	Abortion: 64; No neonatal vital status: 20; twin: 163 (TOTAL: 247; 1.6%)	Abortion: 3; twin/unknown n fetus: 34 (TOTAL: 37; 1.3%)	Abortion: 0; twin/unknown n fetus: 15 (TOTAL: 15; 0.7%)	Abortion: 0; twin/unknown n fetus: 33 (TOTAL: 33; 1.0%)	abortion: 61; no neonatal vital status: 16; twin: 52 (TOTAL: 129; 2.1%)	Abortion: 0; no neonatal vital status: 4; twin/unknown n fetus: 29 (TOTAL: 33; 3.1%)
**Study population:** Singleton pregnancies w/ available neonatal vital status	**15,286**	**2,786**	**2,025**	**3,456**	**6,004**	**1,015**
Exclusion arrows: Baseline weight after 20 gestational weeks, stillbirth, Delivery before 37 gestational weeks	First weight measurement after 20 weeks: 115; stillbirth: 393; preterm birth: 1783; TOTAL: 2291 (15%)	First weight measurement after 20 weeks: 32, stillbirth: 101, preterm birth: 331 (TOTAL: 464; 16.6%)	First weight measurement after 20 weeks: 12, stillbirth: 64, preterm birth: 278 (TOTAL: 354; 17.5%)	First weight measurement after 20 weeks: 0, stillbirth: 52, preterm birth: 376 (TOTAL: 428; 12.4%)	First weight measurement after 20 weeks: 3, stillbirth: 141, preterm birth: 714 (TOTAL: 858; 14.3%)	First weight measurement after 20 weeks: 68, stillbirth: 35, preterm birth: 84 (TOTAL: 187; 18.5%)
Reference Sub-Population: Singleton, term livebirth, early gestation weight	**12,995**	2,322	1,671	3,028	5,146	828

GWG, gestational weight gain

[a] First available pregnancy; delivery ≤ 42 weeks

[b] 2+ antenatal visits with maternal weight measurements

Table B2. Percentage of participants missing data in the overall study population and by cohort

	Overall (N=15286)	AMANHI-Bangladesh (N=2786)	AMANHI-Pakistan (N=2025)	PreSSMat (N=3456)	GARBH-Ini (N=6004)	ZAPPS (N = 1015)
Maternal age (years)	0.1%	0.0%	0.0%	0.0%	0.0%	1.9%
Gestational age at enrollment (Wks)	0.0%	0.0%	0.0%	0.0%	0.0%	0.0%
Gestational age at 1st maternal weight measurement (Wks)	0.0%	0.0%	0.0%	0.0%	0.0%	0.0%
Maternal weight at baseline (Kg)	0.0%	0.0%	0.0%	0.0%	0.0%	0.0%
Maternal height (cm)	0.0%	0.0%	0.0%	0.0%	0.0%	0.0%
Body mass index (Kg/m2)	0.0%	0.0%	0.0%	0.0%	0.0%	0.0%
Underweight	0.0%	0.0%	0.0%	0.0%	0.0%	0.0%
Normal	0.0%	0.0%	0.0%	0.0%	0.0%	0.0%
Overweight	0.0%	0.0%	0.0%	0.0%	0.0%	0.0%
Obese	0.0%	0.0%	0.0%	0.0%	0.0%	0.0%
Parous	0.0%	0.0%	0.0%	0.0%	0.0%	0.0%
Previous preterm birth[a]	1.5%	2.3%	0.6%	0.0%	2.9%	0.0%
Previous stillbirth[a]	0.8%	0.0%	0.0%	0.0%	0.3%	8.7%
Maternal years of education	0.1%	0.1%	0.0%	0.0%	0.0%	0.9%
Chronic Hypertension	0.7%	0.3%	0.9%	1.3%	0.1%	3.1%
Diabetes	0.8%	0.4%	0.8%	2.4%	0.0%	1.3%
Total visits maternal weight measurements	0.0%	0.0%	0.0%	0.0%	0.0%	0.0%
Gestational age at last maternal weight measurement	0.0%	0.0%	0.0%	0.0%	0.0%	0.0%
Total gestational weight gain (Kg)	0.0%	0.0%	0.0%	0.0%	0.0%	0.0%
Gestational age at delivery (Wks)	0.0%	0.0%	0.0%	0.0%	0.0%	0.0%
Spontaneous labor	14.0%	0.0%	4.1%	0.0%	31.9%	13.7%
Cesarean delivery	0.6%	0.0%	2.9%	0.0%	0.0%	3.0%
Preterm birth < 37 weeks	0.0%	0.0%	0.0%	0.0%	0.0%	0.0%
Preterm birth < 32 weeks	0.0%	0.0%	0.0%	0.0%	0.0%	0.0%
Birth weight	12.0%	19.7%	15.2%	17.9%	5.6%	1.6%
Birthweight <2500 g	12.0%	19.7%	15.2%	17.9%	5.6%	1.6%
Birthweight <1500 g	12.0%	19.7%	15.2%	17.9%	5.6%	1.6%
SGA <10th centile	13.9%	19.7%	15.3%	17.9%	10.6%	1.7%
SGA <3rd centile	13.9%	19.7%	15.3%	17.9%	10.6%	1.7%
Stillbirth	0.0%	0.0%	0.0%	0.0%	0.0%	0.0%

Table B3. Distribution of gestational weight gain Z-score[a] by study population characteristics (N= 15286)

Characteristic		Mean (95% CI)	Difference (95% CI)
Overall[a]		-0.02 (-0.03, -0.01)	
Cohort	AMANHI-Bangladesh	-0.02 (-0.06, 0.02)	0.04 (-0.03, 0.12)
	AMANHI-Pakistan	-0.03 (-0.07, 0.02)	0.03 (-0.05, 0.11)
	PreSSMat	0.00 (-0.03, 0.04)	0.07 (-0.01, 0.14)
	GARBH-Ini	-0.02 (-0.05, 0.00)	0.04 (-0.03, 0.11)
	ZAPPS	-0.06 (-0.13, 0.00)	0
Maternal age, years	<20	0.09 (-0.05, 0.22)	0.04 (-0.06, 0.14)
	20-24	0.05 (0.01, 0.09)	0
	25-29	-0.06 (-0.08, -0.04)	-0.11 (-0.16, -0.06)
	30-34	-0.17 (-0.26, -0.08)	-0.22 (-0.33, -0.10)
	35+	-0.22 (-0.35, -0.10)	-0.27 (-0.43, -0.11)
Gestational age at enrollment, quartiles	Lowest (5-10)	0.07 (0.03, 0.12)	0
	Second (11-12)	0.06 (0.02, 0.10)	-0.02 (-0.07, 0.04)
	Third (13-16)	0.00 (-0.03, 0.03)	-0.08 (-0.12, -0.03)
	Highest (16-24)	-0.21 (-0.28, -0.14)	-0.29 (-0.39, -0.19)
Maternal height, quartiles	Lowest (100-148)	-0.14 (-0.19, -0.09)	-0.22 (-0.33, -0.12)
	Second (149-152)	-0.04 (-0.07, 0.00)	-0.12 (-0.20, -0.04)
	Third (153-156)	0.01 (-0.01, 0.04)	-0.07 (-0.15, 0.01)
	Highest (157-190)	0.08 (0.01, 0.16)	0
Body mass index (Kg/m2)	Underweight	-0.03 (-0.03, -0.02)	0.00 (-0.02, 0.01)
	Normal	-0.02 (-0.04, -0.01)	0
	Overweight	0.00 (-0.02, 0.02)	0.03 (0.01, 0.04)
	Obese	0.03 (-0.03, 0.09)	0.05 (0.00, 0.11)
Parous	No	0.11 (0.03, 0.18)	0
	Yes	-0.10 (-0.15, -0.05)	-0.21 (-0.32, -0.09)
Previous preterm birth	No	-0.01 (-0.02, 0.00)	0
	Yes	-0.13 (-0.16, -0.10)	-0.12 (-0.15, -0.08)
Previous stillbirth	No	-0.02 (-0.04, -0.00)	0
	Yes	-0.01 (-0.11, 0.09)	0.01 (-0.11, 0.12)
Maternal years of education	Lowest (0-6)	-0.09 (-0.12, -0.05)	-0.13 (-0.19, -0.07)
	Second (7-10)	-0.00 (-0.04, 0.04)	-0.05 (-0.11, 0.02)
	Third (11-13)	-0.03 (-0.15, 0.10)	-0.07 (-0.18, 0.03)
	Highest (14-17)	0.04 (0.01, 0.07)	0
Chronic Hypertension	No	-0.02 (-0.03, -0.00)	0
	Yes	0.00 (-0.16, 0.17)	0.02 (-0.15, 0.20)
Diabetes	No	-0.02 (-0.03, -0.01)	0
	Yes	0.03 (-0.31, 0.36)	0.04 (-0.29, 0.38)

[a] Weight-gain-for-gestational age z-score based on the reference sub-population standards. For each participant, Z-score calculated as total (weight gain – average weight gain)/standard deviation of weight gain based on the calculated standards for their BMI group (underweight, normal, overweight, obese) and cohort.
Estimates from marginal linear models of gestational weight gain z-score (outcome variable). A separate model was run with each characteristic shown as the single predictor variable.

Table B4. Reference sub-population population characteristics, overall and by study cohort

	Overall (N=12995)	4: AMANHI-B (N=2322)	3: AMANHI-P (N= 1671)	2: GAPPS-B (N= 3028)	6: THSTI-I (N=5146)	1: ZAPPS (N = 828)
Maternal age (years)	24.0 (21.0, 28.0)	23.0 (20.0,26.0)	26.0 (23.0,30.0)	24.0 (21.0,29.0)	23.0 (21.0,26.0)	27.0 (23.0,31.0)
Gestational age at enrollment (Wks)	13.0 (11.1, 16.0)	13.4 (11.3,16.6)	13.4 (10.7,16.7)	12.3 (11.4,13.6)	13.1 (9.6,16.1)	16.1 (13.4,18.1)
Gestational age at first maternal weight (Wks)	12.9 (10.9, 15.9)	12.6 (9.9,15.7)	13.4 (10.7,16.7)	12.3 (11.4,13.6)	13.1 (9.7,16.1)	16.1 (13.4,18.1)
Maternal weight at baseline (Kg)	48.4 (42.9, 55.5)	43.9 (40.0,48.9)	50.5 (45.0,60.0)	49.5 (44.4,56.1)	47.8 (42.7,54.0)	61.0 (54.0,72.0)
Maternal height (cm)	152.5 (148.9, 156.6)	150.1 (146.3,153.4)	153.8 (150.0,158.0)	152.0 (148.4,155.5)	153.2 (149.3,157.0)	160.0 (156.0,164.0)
BMI (Kg/m2)	20.7 (18.7, 23.5)	19.5 (18.0,21.5)	21.5 (18.8,25.1)	21.5 (19.4,24.2)	20.3 (18.4,22.8)	23.9 (21.4,27.7)
Underweight	22.8% (2959)	32.3% (749)	21.2% (355)	16.1% (488)	25.8% (1326)	5.0% (41)
Normal	60.7% (7893)	61.5% (1427)	53.1% (888)	64.4% (1950)	61.9% (3184)	53.6% (444)
Overweight	13.2% (1718)	5.5% (127)	18.7% (312)	17.0% (515)	10.7% (552)	25.6% (212)
Obese	3.3% (425)	0.8% (19)	6.9% (116)	2.5% (75)	1.6% (84)	15.8% (131)
Parous (1+ previous delivery)	59.8% (7774)	64.9% (1508)	75.2% (1257)	61.9% (1873)	49.9% (2570)	68.4% (566)
Previous preterm birth[a]	9.5% (735)	5.5% (84)	7.4% (93)	6.0% (112)	9.7% (248)	35.0% (198)
Previous stillbirth[a]	6.9% (534)	10.7% (162)	7.0% (88)	3.9% (73)	5.5% (140)	12.5% (71)
Maternal years of education	10.0 (6.0, 13.0)	7.0 (5.0,9.0)	0.0 (0.0,8.0)	8.0 (5.0,10.0)	14.0 (12.0,15.0)	12.0 (9.0,12.0)
Chronic Hypertension	1.7% (227)	0.2% (5)	5.3% (88)	0.8% (25)	0.3% (13)	11.6% (96)
Diabetes	0.3% (42)	0.3% (6)	0.6% (10)	0.4% (13)	0.1% (5)	0.9% (7)
Total maternal weight measurements	4.0 (4.0, 5.0)	4.0 (4.0,4.0)	4.0 (3.0,4.0)	5.0 (4.0,5.0)	4.0 (3.0,5.0)	5.0 (4.0,5.0)
Gestational age at last maternal weight	37.0 (33.0, 38.9)	36.9 (35.1,37.7)	38.0 (32.9,38.6)	36.3 (34.7,38.9)	38.1 (30.7,39.6)	36.1 (35.9,36.6)
Total GWG (Kg)	7.0 (4.4, 9.7)	6.3 (3.9,8.8)	6.8 (4.0,9.5)	7.6 (5.3,10.0)	6.9 (4.3,9.7)	7.9 (4.5,11.0)
Total GWG, participants w/ both 2nd&3rd trimester weight[b]	7.3 (5.0, 9.9)	6.5 (4.0, 8.8)	7.1 (4.5, 9.9)	7.7 (5.5, 10.2)	7.5 (5.2, 10.1)	8.0 (5.0, 11.0)
2nd trimester GWG (Kg)[b]	3.5 (1.9, 5.2)	2.9 (1.5, 4.5)	3.5 (1.8, 5.5)	3.5 (2.2, 5.2)	3.8 (2.1, 5.5)	3.0 (1.1, 5.4)
3rd trimester GWG (Kg)[b]	7.3 (4.9, 9.8)	6.5 (4.0, 8.8)	7.1 (4.4, 9.9)	7.6 (5.5, 10.1)	7.4 (5.1, 9.9)	8.0 (5.0, 11.0)
GWG Z-score percentile[c]	54.5 (29.7, 75.2)	56.9 (32.6, 74.4)	55.2 (31.4, 72.4)	54.1 (28.0, 76.7)	52.8 (28.6, 75.5)	54.0 (30.3, 76.0)
GWG Z-score percentile (normal BMI)[d]	54.6 (29.4, 74.8)	57.7 (31.8, 75.7)	55.2 (32.3, 70.7)	54.1 (27.6, 76.7)	52.6 (28.0, 74.7)	52.3 (31.4, 72.8)
IG-21 GWG Z-score percentile (normal BMI)[d,e]	10.0 (1.8, 29.2)	4.6 (0.4, 18.0)	9.3 (1.3, 24.6)	14.0 (3.2, 34.2)	11.0 (2.5, 29.7)	15.7 (2.7, 48.6)
IOM adequacy ratio[f]	61.8 (41.7, 84.7)	52.7 (32.6, 71.4)	63.2 (39.7, 87.9)	68.1 (49.0, 90.0)	60.4 (41.6, 82.6)	77.2 (48.2, 114.9)
IOM adequacy ratio (normal BMI)[d]	59.7 (39.8, 80.6)	52.0 (30.8, 71.8)	59.7 (38.2, 79.2)	66.2 (47.1, 85.7)	58.3 (38.7, 78.3)	69.0 (44.3, 97.6)
Gestational age at delivery (Wks)	39.3 (38.4, 40.1)	39.4 (38.6,40.1)	39.3 (38.4,40.0)	39.3 (38.4,40.0)	39.4 (38.4,40.1)	39.7 (39.0,40.4)
Spontaneous labor	79.7% (10356)	86.4% (2006)	84.5% (1412)	55.2% (1671)	88.8% (4571)	84.1% (696)
Cesarean delivery	25.7% (3337)	13.6% (316)	15.5% (259)	45.2% (1369)	23.5% (1207)	22.4% (185)
Preterm birth < 37 weeks	0.0% (0)	0.0% (0)	0.0% (0)	0.0% (0)	0.0% (0)	0.0% (0)
Preterm birth < 32 weeks	0.0% (0)	0.0% (0)	0.0% (0)	0.0% (0)	0.0% (0)	0.0% (0)
Birth weight	2850 (2568, 3136)	2768 (2500,3030)	2845 (2580,3090)	2900 (2640,3200)	2810 (2514,3100)	3148 (2900,3400)
Birthweight <2500 g	17.7% (2305)	24.7% (573)	17.5% (293)	13.6% (412)	19.2% (987)	4.7% (39)
Birthweight <1500 g	0.1% (14)	0.2% (5)	0.0% (0)	0.1% (3)	0.1% (6)	0.0% (0)
SGA <10th centile	37.1% (4817)	45.9% (1065)	36.0% (602)	30.5% (924)	40.6% (2088)	16.7% (138)
SGA <3rd centile	17.4% (2262)	23.8% (552)	16.5% (276)	12.6% (381)	19.4% (999)	6.5% (54)
Stillbirth	0.0% (0)	0.0% (0)	0.0% (0)	0.0% (0)	0.0% (0)	0.0% (0)

Data shown as % (n) or median (IQR)

Abbreviations. BMI, body mass index; IG-21, INTERGROWTH-21st; IOM, Institute of Medicine GWG, Gestational weight gain

[a] Among N=7774 parous participants.

[b] Among N=11226 participants with available 2nd *and* 3rd trimester weight gain

[c] Weight-gain-for-gestational-age Z-score based on BMI- and cohort-specific reference GWG values estimated from the study sub-population

[d] Restricted to N=7109 normal BMI participants with final weight measurement at 14-40 weeks of gestation (the target population of INTERGROWTH-21 weight-gain-for-gestational-age standards) (https://doi.org/10.1136/bmj.i555)

[e] Weight-gain-for-gestational-age Z-score based on INTERGROWTH-21 weight-gain-for-gestational-age standards (https://doi.org/10.1136/bmj.i555)
[f] Ratio of (observed/recommended weight gain)*100. Recommended weight gain calculated as: 1st trimester recommended weight gain + [2nd & 3rd trimester recommended weekly weight gain rate*(final gestational age weeks-13)]. We assumed a 1st trimester weight gain of 2kg for underweight/normal BMI participants and of 0.5kg for overweight/obese. We assumed a 2nd & 3rd trimester weight gain rate of 0.51, 0.42, 0.28, 0.22 kg/week for underweight, normal, overweight, and obese respectively. IOM 2009 guidelines: https://doi.org/10.17226/12584.

Table B5. Cohort-specific risk ratios for the association between gestational weight gain Z-score and preterm birth (<37 weeks)

		AMANHI-Pakistan			AMANHI-Bangladesh			PreSSMat[b]			GARBH-Ini			ZAPPS		
BMI Group	GWG group	Risk (n/N)	Adj Risk,[a] % (95% CI)	Adj RR[a], (95% CI)	Risk (n/N)	Adj Risk,[a] % (95% CI)	Adj RR[a], (95% CI)	Risk (n/N)	Adj Risk,[a] % (95% CI)	Adj RR[a], (95% CI)	Risk (n/N)	Adj Risk,[a] % (95% CI)	Adj RR[a], (95% CI)	Risk (n/N)	Adj Risk,[a] % (95% CI)	Adj RR[a], (95% CI)
1	1	21/79	26.7 (18.6, 38.3)	1.96 (1.13, 3.40)	24/161	14.3 (9.8, 20.9)	1.09 (0.69, 1.70)	12/110	7.7 (4.4, 13.6)	0.75 (0.37, 1.51)	65/334	19.9 (16.0, 24.7)	1.75 (1.26, 2.44)	1/6		
	2	22/135	16.2 (11.1, 23.7)	1.20 (0.69, 2.08)	47/250	16.7 (12.8, 21.8)	1.27 (0.89, 1.82)	16/152	8.7 (5.3, 14.3)	0.84 (0.45, 1.59)	59/449	12.1 (9.5, 15.3)	1.06 (0.75, 1.50)	3/16		
	3	23/153	13.6 (9.1, 20.3)	1	49/342	13.2 (10.1, 17.2)	1	19/168	10.4 (6.7, 16.1)	1.00 (1.00, 1.00)	55/444	11.3 (8.8, 14.7)	1	1/19		
	4	21/86	20.1 (13.6, 29.8)	1.48 (0.88, 2.51)	22/167	11.5 (7.9, 16.8)	0.87 (0.55, 1.38)	9/119	6.3 (3.4, 11.8)	0.61 (0.29, 1.26)	43/340	11.4 (8.5, 15.2)	1.00 (0.69, 1.46)	1/6		
2	1	24/195	10.9 (7.5, 15.9)	1.44 (0.88, 2.35)	40/340	11.3 (8.3, 15.2)	1.04 (0.72, 1.51)	60/505	10.5 (8.1, 13.5)	1.02 (0.73, 1.43)	114/837	12.4 (10.4, 14.8)	1.08 (0.85, 1.37)	16/123	10.8 (6.7, 17.7)	1.03 (0.54, 1.96)
	2	36/259	12.6 (9.2, 17.4)	1.67 (1.06, 2.62)	54/374	13.6 (10.5, 17.5)	1.25 (0.89, 1.76)	68/523	11.3 (9.0, 14.3)	1.11 (0.80, 1.52)	119/901	12.6 (10.6, 14.9)	1.09 (0.86, 1.38)	18/142	11.5 (7.5, 17.8)	1.09 (0.60, 1.99)
	3	32/364	7.6 (5.4, 10.6)	1	64/548	10.8 (8.5, 13.7)	1	65/611	10.2 (8.2, 12.8)	1	121/1009	11.5 (9.7, 13.6)	1	22/169	10.5 (6.7, 16.6)	1
	4	40/226	15.1 (11.2, 20.3)	1.99 (1.31, 3.03)	45/434	10.2 (7.7, 13.4)	0.94 (0.65, 1.35)	56/573	9.4 (7.3, 12.1)	0.92 (0.66, 1.28)	103/931	10.6 (8.8, 12.7)	0.92 (0.72, 1.17)	10/114	6.5 (3.5, 12.2)	0.62 (0.30, 1.28)
3	1	17/94	16.7 (10.7, 26.1)	1.15 (0.61, 2.14)	4/36			19/146	10.3 (6.8, 15.4)	1.16 (0.63, 2.11)	25/159	13.6 (9.3, 19.8)	1.32 (0.77, 2.26)	3/62	2.9 (1.1, 7.3)	0.19 (0.07, 0.52)
	2	10/68	13.3 (7.2, 24.4)	0.91 (0.44, 1.89)	5/24			20/130	14.4 (9.5, 21.9)	1.62 (0.86, 3.06)	13/107	11.0 (6.6, 18.3)	1.06 (0.56, 2.02)	10/66	11.3 (6.0, 21.4)	0.74 (0.37, 1.47)
	3	16/103	14.6 (9.3, 22.9)	1	2/38			16/143	8.9 (5.4, 14.6)	1.00 (1.00, 1.00)	20/178	10.3 (6.8, 15.6)	1	15/66	15.4 (9.2, 25.6)	1
	4	21/119	16.4 (11.0, 24.4)	1.13 (0.62, 2.06)	5/50			25/179	13.6 (9.3, 19.8)	1.53 (0.83, 2.80)	31/205	13.7 (9.8, 19.1)	1.33 (0.79, 2.23)	9/75	7.2 (3.7, 14.0)	0.47 (0.22, 1.00)
4	1	7/31	19.5 (9.7, 39.2)	2.61 (0.78, 8.75)	0/8			4/24	11.5 (4.6, 28.9)	0.77 (0.27, 2.18)	6/26	19.5 (8.6, 43.9)	0.59 (0.23, 1.53)	2/39		
	2	8/33	17.1 (9.3, 31.2)	2.29 (0.75, 6.94)	0/1			3/14	21.7 (7.3, 63.8)	1.46 (0.36, 5.82)	3/26	10.6 (4.0, 28.5)	0.32 (0.09, 1.11)	2/19		
	3	4/42	7.5 (2.6, 21.5)	1	0/1			5/21	14.9 (6.5, 33.9)	1.00 (1.00, 1.00)	8/15	33.0 (17.2, 63.3)	1	3/39		
	4	7/38	14.0 (6.4, 30.8)	1.87 (0.51, 6.90)	3/12			10/38	12.1 (4.8, 30.6)	0.81 (0.33, 2.02)	5/43	11.4 (4.8, 27.0)	0.34 (0.13, 0.94)	7/54		

BMI Category. 1: underweight; 2: normal; 3: overweight; 4: obese

GWG group z-score ranges (percentile). 1: ≤ -0.67 (≤ 25th percentile); 2: -0.68 - 0.00 (26 - 50th percentile); 3: 0.01 - 0.67 (51 - 75th percentile); 4: > 0.67 (> 75th percentile)

[a] Adjusted for maternal age, gestational age at enrollment, maternal height, maternal BMI, parous, previous preterm birth

[b] For PreSSMat only: reporting results from complete-case analysis for PTB among the underweight, overweight, and obese because those participants did not have any missing covariate data

Table B6. Cohort-specific risk ratios for the association between gestational weight gain Z-score and low birthweight (<2500 Kg)

		AMANHI-Pakistan			AMANHI-Bangladesh			PreSSMat			GARBH-Ini			ZAPPS		
BMI Group	GWG group	Risk (n/N)	Adj Risk,[a] % (95% CI)	Adj RR[a], (95% CI)	Risk (n/N)	Adj Risk,[a] % (95% CI)	Adj RR[a], (95% CI)	Risk (n/N)	Adj Risk,[a] % (95% CI)	Adj RR[a], (95% CI)	Risk (n/N)	Adj Risk,[a] % (95% CI)	Adj RR[a], (95% CI)	Risk (n/N)	Adj Risk,[a] % (95% CI)	Adj RR[a], (95% CI)
1	1	41/79	54.2 (43.5, 67.5)	2.11 (1.49, 2.99)	82/161	49.8 (42.0, 59.1)	1.56 (1.24, 1.96)	36/110	28.2 (20.6, 38.6)	1.36 (0.86, 2.15)	157/334	47.1 (41.7, 53.2)	1.77 (1.45, 2.16)	1/4		
	2	44/135	31.7 (24.4, 41.2)	1.23 (0.85, 1.79)	127/250	48.1 (42.0, 55.0)	1.51 (1.23, 1.84)	41/152	25.2 (19.0, 33.5)	1.22 (0.80, 1.86)	150/449	31.5 (27.5, 36.2)	1.19 (0.97, 1.45)	5/15		
	3	43/153	25.7 (19.6, 33.7)	1	113/342	31.9 (27.3, 37.3)	1	35/168	20.7 (14.9, 28.8)	1	123/444	26.6 (22.8, 31.1)	1	2/18		
	4	29/86	28.3 (20.5, 39.1)	1.10 (0.74, 1.65)	41/167	23.0 (17.5, 30.1)	0.72 (0.53, 0.98)	21/119	16.7 (11.0, 25.4)	0.81 (0.48, 1.36)	73/340	20.6 (16.7, 25.4)	0.78 (0.60, 1.00)	0/6		
2	1	65/195	32.9 (26.5, 40.8)	1.70 (1.26, 2.29)	127/340	36.0 (30.7, 42.3)	1.50 (1.20, 1.87)	137/505	25.9 (22.1, 30.2)	1.49 (1.18, 1.89)	290/837	33.5 (30.4, 36.9)	1.44 (1.24, 1.67)	23/123	14.3 (9.6, 21.2)	1.37 (0.78, 2.41)
	2	72/259	25.6 (20.7, 31.6)	1.32 (0.98, 1.78)	127/374	32.0 (27.5, 37.3)	1.33 (1.08, 1.65)	122/523	20.9 (17.7, 24.8)	1.21 (0.95, 1.54)	231/901	24.6 (21.9, 27.6)	1.06 (0.90, 1.24)	23/142	14.4 (9.7, 21.4)	1.38 (0.81, 2.37)
	3	74/364	19.4 (15.6, 24.1)	1	142/548	24.0 (20.6, 28.1)	1	112/611	17.3 (14.4, 20.8)	1	241/1009	23.3 (20.7, 26.1)	1	23/169	10.4 (6.8, 15.9)	1
	4	46/226	18.2 (13.7, 24.2)	0.94 (0.66, 1.33)	100/434	22.1 (18.4, 26.5)	0.92 (0.73, 1.16)	55/573	9.2 (6.9, 12.1)	0.53 (0.38, 0.74)	149/931	15.6 (13.5, 18.2)	0.67 (0.56, 0.81)	12/114	7.8 (4.4, 13.6)	0.75 (0.39, 1.43)
3	1	26/94	24.1 (16.9, 34.5)	1.37 (0.80, 2.35)	6/36			30/146	16.3 (11.3, 23.7)	1.38 (0.80, 2.38)	40/159	22.5 (16.8, 30.2)	1.69 (1.10, 2.58)	3/62	3.4 (1.1, 10.9)	0.30 (0.08, 1.07)
	2	13/68	16.6 (9.3, 29.8)	0.94 (0.47, 1.89)	6/24			26/130	18.5 (12.7, 26.7)	1.56 (0.87, 2.79)	21/107	17.6 (12.0, 25.8)	1.32 (0.80, 2.17)	9/66	11.6 (6.2, 21.7)	1.01 (0.47, 2.21)
	3	20/103	17.6 (11.2, 27.8)	1	6/38			20/143	11.8 (7.4, 19.0)	1	28/178	13.4 (9.5, 18.9)	1	10/66	11.4 (6.5, 20.2)	1
	4	15/119	11.7 (7.3, 18.9)	0.67 (0.34, 1.29)	6/50			17/179	9.3 (5.6, 15.6)	0.79 (0.40, 1.55)	30/205	12.8 (9.0, 18.3)	0.96 (0.59, 1.55)	10/75	11.6 (5.9, 22.6)	1.01 (0.44, 2.33)
4	1	12/31	32.2 (18.6, 55.5)	4.78 (1.54, 14.82)	0/8			5/24	1.7 (0.0, 367.6)	1.33 (0.38, 4.60)	5/26	0.5 (0.0, 563.3)	0.74 (0.25, 2.19)	3/39	5.9 (1.4, 24.9)	1.44 (0.18, 11.39)
	2	8/33	21.0 (10.7, 41.2)	3.12 (0.96, 10.22)	0/1			2/14	2.3 (0.0, 571.6)	1.81 (0.30, 10.94)	3/26	0.3 (0.0, 371.9)	0.53 (0.12, 2.35)	3/19	11.4 (4.1, 31.3)	2.79 (0.40, 19.33)
	3	4/42	6.7 (2.3, 19.8)	1	0/1			3/21	1.3 (0.0, 294.1)	1	6/15	0.6 (0.0, 788.4)	1	2/39	4.1 (0.6, 28.9)	1
	4	4/38	7.2 (1.6, 31.9)	1.07 (0.20, 5.78)	2/12			5/38	0.9 (0.0, 206.1)	0.73 (0.15, 3.67)	6/43	0.4 (0.0, 512.1)	0.57 (0.19, 1.73)	5/54	6.1 (2.6, 14.4)	1.50 (0.24, 9.36)

BMI Category. 1: underweight; 2: normal; 3: overweight; 4: obese

GWG group z-score ranges (percentile). 1: ≤ -0.67 (≤ 25th percentile); 2: -0.68 - 0.00 (26 - 50th percentile); 3: 0.01 - 0.67 (51 - 75th percentile); 4: > 0.67 (> 75th percentile)

[a] Adjusted for maternal age, gestational age at enrollment, maternal height, maternal BMI, parous, previous preterm birth

Table B7. Cohort-specific risk ratios for the association between gestational weight gain Z-score and small for gestational age (<10th percentile)

		AMANHI-Pakistan			AMANHI-Bangladesh			PreSSMat			GARBH-Ini			ZAPPS		
BMI Group	GWG group	Risk (n/N)	Adj Risk,[a] % (95% CI)	Adj RR[a], (95% CI)	Risk (n/N)	Adj Risk,[a] % (95% CI)	Adj RR[a], (95% CI)	Risk (n/N)	Adj Risk,[a] % (95% CI)	Adj RR[a], (95% CI)	Risk (n/N)	Adj Risk,[a] % (95% CI)	Adj RR[a], (95% CI)	Risk (n/N)	Adj Risk,[a] % (95% CI)	Adj RR[a], (95% CI)
1	1	38/79	49.3 (38.9, 62.4)	1.19 (0.88, 1.60)	99/161	59.4 (51.5, 68.5)	1.25 (1.04, 1.50)	62/110	52.0 (42.3, 64.0)	1.46 (1.09, 1.97)	199/334	57.0 (51.7, 62.8)	1.36 (1.17, 1.58)	2/6		
	2	66/135	47.5 (39.3, 57.5)	1.15 (0.89, 1.48)	162/250	63.2 (57.2, 69.9)	1.33 (1.15, 1.55)	54/152	34.3 (27.3, 43.2)	0.96 (0.70, 1.32)	217/448	47.4 (42.9, 52.4)	1.13 (0.98, 1.31)	4/16		
	3	68/153	41.5 (34.4, 50.1)	1	164/342	47.4 (42.2, 53.3)	1	61/168	35.6 (28.6, 44.3)	1	187/442	41.9 (37.4, 46.8)	1	3/19		
	4	30/86	31.3 (23.0, 42.7)	0.76 (0.53, 1.07)	56/167	32.5 (25.9, 40.7)	0.68 (0.53, 0.88)	35/119	29.2 (21.5, 39.9)	0.82 (0.56, 1.20)	134/340	40.0 (34.9, 45.8)	0.95 (0.80, 1.14)	0/6		
2	1	95/195	49.1 (42.2, 57.1)	1.59 (1.28, 1.98)	178/340	50.7 (45.3, 56.8)	1.39 (1.19, 1.63)	205/505	39.8 (35.4, 44.8)	1.53 (1.28, 1.83)	411/834	48.9 (45.5, 52.6)	1.36 (1.22, 1.51)	33/122	26.0 (18.7, 36.2)	1.63 (1.01, 2.61)
	2	100/259	36.9 (31.2, 43.5)	1.20 (0.95, 1.50)	175/374	45.4 (40.4, 50.9)	1.24 (1.06, 1.46)	182/522	33.0 (29.0, 37.5)	1.27 (1.05, 1.53)	369/899	39.4 (36.3, 42.7)	1.09 (0.98, 1.23)	38/142	26.2 (19.7, 34.7)	1.64 (1.06, 2.53)
	3	117/364	30.8 (26.4, 36.0)	1	210/548	36.5 (32.5, 41.0)	1	167/611	26.0 (22.5, 30.0)	1	375/1005	36.0 (33.1, 39.1)	1	28/168	16.0 (11.4, 22.4)	1
	4	62/226	25.5 (20.2, 32.2)	0.83 (0.63, 1.09)	146/434	32.2 (27.9, 37.1)	0.88 (0.74, 1.05)	118/573	20.0 (16.8, 23.9)	0.77 (0.62, 0.96)	257/928	26.8 (24.1, 29.8)	0.75 (0.65, 0.85)	15/114	12.7 (7.8, 20.5)	0.79 (0.44, 1.43)
3	1	29/94	27.7 (19.9, 38.7)	1.20 (0.75, 1.91)	10/36	24.1 (11.8, 49.2)	1.30 (0.54, 3.11)	44/146	27.8 (21.0, 36.8)	1.56 (1.00, 2.44)	47/159	29.0 (22.6, 37.3)	1.34 (0.95, 1.90)	13/62	17.5 (9.8, 31.1)	1.60 (0.74, 3.46)
	2	18/68	24.3 (15.8, 37.1)	1.05 (0.61, 1.80)	6/24	18.6 (8.3, 41.5)	1.00 (0.39, 2.58)	32/130	24.2 (17.7, 33.2)	1.36 (0.84, 2.18)	31/107	27.1 (20.0, 36.8)	1.26 (0.86, 1.84)	10/66	12.0 (6.9, 20.7)	1.09 (0.52, 2.31)
	3	26/103	23.1 (16.1, 33.3)	1	10/38	18.5 (10.0, 34.3)	1	27/143	17.8 (12.4, 25.7)	1	47/177	21.6 (16.6, 28.1)	1	9/66	10.9 (6.2, 19.4)	1
	4	25/119	19.4 (13.3, 28.2)	0.84 (0.51, 1.39)	12/50	21.5 (11.8, 39.2)	1.16 (0.53, 2.56)	22/179	11.7 (7.4, 18.6)	0.66 (0.37, 1.19)	41/205	17.5 (13.2, 23.1)	0.81 (0.56, 1.16)	8/75	9.8 (4.8, 19.8)	0.90 (0.38, 2.12)
4	1	10/31	24.1 (11.4, 50.9)	1.59 (0.62, 4.07)	1/8			6/24			5/25	16.1 (5.4, 47.7)	2.22 (0.39,12.71)	3/38	6.6 (1.7, 25.9)	1.44 (0.22, 9.26)
	2	6/33	18.4 (7.9, 43.0)	1.21 (0.39, 3.81)	0/1			1/14			7/26	15.9 (6.9, 37.1)	2.20 (0.37,12.96)	4/19	11.7 (4.9, 27.9)	2.55 (0.66, 9.82)
	3	8/42	15.2 (7.3, 31.5)	1	0/1			1/21			4/15	7.2 (1.0, 54.5)	1	3/39	4.6 (1.1, 18.5)	1
	4	3/38	5.0 (1.5, 16.6)	0.33 (0.09, 1.23)	3/12			5/38			6/43	7.2 (3.2, 15.8)	0.99 (0.17, 5.64)	5/54	6.6 (2.5, 17.6)	1.44 (0.29, 7.03)

BMI Category. 1: underweight; 2: normal; 3: overweight; 4: obese

GWG group z-score ranges (percentile). 1: ≤ -0.67 (≤ 25th percentile); 2: -0.68 - 0.00 (26 - 50th percentile); 3: 0.01 - 0.67 (51 - 75th percentile); 4: > 0.67 (> 75th percentile)

[a] Adjusted for maternal age, gestational age at enrollment, maternal height, maternal BMI, parous, previous preterm birth

Table B8. Cohort-specific risk ratios for the association between gestational weight gain Z-score and small for gestational age (<3rd percentile)

		AMANHI-Pakistan			AMANHI-Bangladesh			PreSSMat			GARBH-Ini			ZAPPS		
BMI Group	GWG group	Risk (n/N)	Adj Risk,[a] % (95% CI)	Adj RR[a], (95% CI)	Risk (n/N)	Adj Risk,[a] % (95% CI)	Adj RR[a], (95% CI)	Risk (n/N)	Adj Risk,[a] % (95% CI)	Adj RR[a], (95% CI)	Risk (n/N)	Adj Risk,[a] % (95% CI)	Adj RR[a], (95% CI)	Risk (n/N)	Adj Risk,[a] % (95% CI)	Adj RR[a], (95% CI)
1	1	21/79	24.7 (16.5, 37.2)	1.49 (0.89, 2.48)	56/161	33.2 (26.2, 42.1)	1.63 (1.18, 2.24)	33/110	24.7 (17.2, 35.4)	2.15 (1.23, 3.74)	108/334	30.3 (25.5, 35.9)	1.40 (1.09, 1.79)	1/6		
	2	33/135	21.4 (14.9, 30.6)	1.29 (0.81, 2.04)	93/250	34.6 (29.0, 41.3)	1.69 (1.28, 2.24)	29/152	16.8 (11.6, 24.3)	1.46 (0.84, 2.55)	112/448	24.1 (20.3, 28.5)	1.11 (0.87, 1.43)	4/16		
	3	29/153	16.6 (11.8, 23.4)	1	73/342	20.4 (16.4, 25.5)	1	20/168	11.5 (7.3, 18.0)	1	98/442	21.6 (18.0, 26.0)	1	2/19		
	4	12/86	11.6 (6.2, 21.6)	0.70 (0.35, 1.40)	27/167	15.2 (10.5, 21.9)	0.74 (0.49, 1.14)	13/119	11.0 (6.4, 18.7)	0.96 (0.49, 1.87)	57/340	16.9 (13.1, 21.7)	0.78 (0.57, 1.06)	0/6		
2	1	45/195	22.3 (16.7, 29.6)	1.75 (1.19, 2.56)	96/340	26.6 (21.7, 32.7)	1.44 (1.10, 1.89)	94/505	17.9 (14.7, 21.9)	1.61 (1.19, 2.18)	209/834	24.3 (21.5, 27.5)	1.49 (1.24, 1.79)	13/122	6.5 (3.4, 12.6)	1.14 (0.51, 2.52)
	2	55/259	18.8 (14.5, 24.3)	1.47 (1.02, 2.12)	101/374	25.4 (21.3, 30.4)	1.38 (1.08, 1.77)	67/522	11.5 (8.9, 14.7)	1.03 (0.73, 1.43)	161/899	16.6 (14.3, 19.2)	1.02 (0.83, 1.24)	11/142	4.8 (2.5, 9.4)	0.84 (0.40, 1.74)
	3	51/364	12.8 (9.7, 16.8)	1	110/548	18.5 (15.3, 22.3)	1	75/611	11.2 (8.8, 14.1)	1	175/1005	16.3 (14.2, 18.8)	1	14/168	5.7 (3.2, 10.2)	1
	4	23/226	8.9 (5.8, 13.5)	0.70 (0.43, 1.14)	61/434	13.1 (10.1, 17.0)	0.71 (0.52, 0.96)	33/573	5.2 (3.6, 7.6)	0.47 (0.31, 0.72)	115/928	11.6 (9.7, 13.8)	0.71 (0.57, 0.88)	6/114	4.1 (1.8, 9.0)	0.71 (0.28, 1.85)
3	1	9/94	6.9 (3.3, 14.4)	0.98 (0.41, 2.33)	4/36	7.7 (1.5, 39.4)		21/146	11.1 (6.8, 18.3)	2.69 (1.10, 6.57)	20/159	9.9 (6.2, 15.9)	1.08 (0.61, 1.90)	5/62	4.1 (1.1, 15.5)	1.11 (0.26, 4.75)
	2	4/68	4.2 (1.4, 13.1)	0.60 (0.17, 2.07)	3/24	5.9 (1.0, 34.8)		10/130	6.8 (3.6, 12.6)	1.64 (0.60, 4.50)	15/107	11.5 (7.1, 18.7)	1.25 (0.68, 2.31)	2/66	2.0 (0.5, 7.6)	0.54 (0.11, 2.51)
	3	12/103	7.0 (3.5, 14.1)	1	6/38	8.0 (2.7, 23.8)		7/143	4.1 (1.8, 9.6)	1	24/177	9.2 (6.0, 14.2)	1	4/66	3.7 (1.2, 10.8)	1
	4	8/119	4.9 (2.4, 9.8)	0.69 (0.28, 1.70)	4/50	5.9 (1.7, 20.5)		6/179	2.8 (1.1, 7.2)	0.68 (0.20, 2.27)	19/205	7.0 (4.4, 11.1)	0.76 (0.42, 1.36)	3/75	3.7 (1.2, 11.9)	1.01 (0.19, 5.29)
4	1	5/31	5.4 (1.5, 18.7)	1.24 (0.27, 5.78)	1/8			4/24			2/25	0.6 (0.0, 11.7)	0.74 (0.08, 7.19)	2/38	4.5 (0.8, 26.2)	1.23 (0.09, 16.19)
	2	3/33	6.9 (1.9, 24.5)	1.60 (0.32, 7.97)	0/1			0/14			2/26	0.5 (0.0, 7.2)	0.59 (0.04, 9.65)	3/19	7.5 (2.9, 19.7)	2.06 (0.35, 12.06)
	3	3/42	4.3 (1.2, 15.3)	1	0/1			0/21			4/15	0.8 (0.0, 20.6)	1	2/39	3.6 (0.6, 23.9)	1
	4	2/38	2.3 (0.5, 11.5)	0.53 (0.09, 3.05)	1/12			3/38			4/43	0.4 (0.0, 7.1)	0.50 (0.09, 2.72)	3/54	4.2 (1.2, 14.9)	1.17 (0.12, 11.40)

BMI Category. 1: underweight; 2: normal; 3: overweight; 4: obese

GWG group z-score ranges (percentile). 1: ≤ -0.67 (≤ 25th percentile); 2: -0.68 - 0.00 (26 - 50th percentile); 3: 0.01 - 0.67 (51 - 75th percentile); 4: > 0.67 (> 75th percentile)

[a] Adjusted for maternal age, gestational age at enrollment, maternal height, maternal BMI, parous, previous preterm birth

Table B9. Risk ratios for the association between gestational weight gain Z-score and adverse outcomes, among participants without previous preterm birth.

	BMI	Weight gain z-score (percentile)	Risk (n/N)	Adjusted Risk,[a] % (95% CI)	Adjusted Risk Ratio[a] (95% CI)
Preterm birth	Underweight	<= -0.67 (≤25th)	110/655	16.8 (13.3, 21.2)	1.38 (1.10, 1.73)
		-0.68 - 0.00 (26-50th)	129/948	13.0 (10.4, 16.1)	1.07 (0.89, 1.28)
		0.01 - 0.67 (51-75th)	137/1074	12.1 (11.7, 12.6)	1
		> 0.67 (>75th)	87/675	11.8 (9.3, 15.0)	0.97 (0.78, 1.20)
	Normal	<= -0.67 (≤25th)	219/1849	11.5 (11.0, 12.0)	1.13 (1.05, 1.22)
		-0.68 - 0.00 (26-50th)	256/2063	12.1 (11.5, 12.6)	1.19 (1.08, 1.31)
		0.01 - 0.67 (51-75th)	266/2562	10.2 (9.4, 11.0)	1
		> 0.67 (>75th)	217/2145	10.0 (9.1, 11.0)	0.98 (0.84, 1.15)
	Overweight	<= -0.67 (≤25th)	56/435	12.0 (9.0, 15.9)	1.13 (0.81, 1.58)
		-0.68 - 0.00 (26-50th)	47/354	13.1 (10.7, 16.0)	1.23 (0.89, 1.70)
		0.01 - 0.67 (51-75th)	54/479	10.6 (8.0, 14.1)	1
		> 0.67 (>75th)	78/581	12.3 (10.2, 14.9)	1.16 (0.83, 1.63)
	Obese	<= -0.67 (≤25th)	14/102	13.3 (6.3, 27.9)	1.02 (0.42, 2.49)
		-0.68 - 0.00 (26-50th)	11/82	10.9 (5.9, 20.1)	0.83 (0.27, 2.61)
		0.01 - 0.67 (51-75th)	15/95	13.1 (6.7, 25.4)	1
		> 0.67 (>75th)	27/155	14.2 (10.6, 19.0)	1.09 (0.58, 2.03)
Low birthweight	Underweight	<= -0.67 (≤25th)	296/655	44.4 (38.4, 51.4)	1.62 (1.45, 1.80)
		-0.68 - 0.00 (26-50th)	337/948	34.3 (27.5, 42.9)	1.25 (1.07, 1.46)
		0.01 - 0.67 (51-75th)	304/1074	27.5 (24.5, 30.7)	1
		> 0.67 (>75th)	150/675	21.0 (17.9, 24.7)	0.77 (0.66, 0.89)
	Normal	<= -0.67 (≤25th)	584/1849	30.7 (27.6, 34.2)	1.52 (1.42, 1.61)
		-0.68 - 0.00 (26-50th)	525/2063	24.1 (21.6, 26.9)	1.19 (1.07, 1.32)
		0.01 - 0.67 (51-75th)	551/2562	20.3 (18.0, 22.8)	1
		> 0.67 (>75th)	321/2145	14.2 (11.2, 18.1)	0.70 (0.59, 0.84)
	Overweight	<= -0.67 (≤25th)	91/435	18.7 (14.9, 23.5)	1.42 (1.22, 1.66)
		-0.68 - 0.00 (26-50th)	62/354	16.4 (14.6, 18.4)	1.24 (1.05, 1.47)
		0.01 - 0.67 (51-75th)	71/479	13.2 (10.9, 15.8)	1
		> 0.6 (>75th)	70/581	11.2 (9.6, 13.2)	0.85 (0.69, 1.05)
	Obese	<= -0.67 (≤25th)	18/102	16.1 (7.1, 36.3)	1.44 (0.51, 4.00)
		-0.68 - 0.00 (26-50th)	10/82	11.1 (6.7, 18.4)	0.99 (0.42, 2.35)
		0.01 - 0.67 (51-75th)	14/95	11.2 (7.1, 17.7)	1
		> 0.67 (>75th)	21/155	9.8 (7.3, 13.3)	0.88 (0.54, 1.44)
Small-for-gestational-age <10th	Underweight	<= -0.67 (≤25th)	382/655	56.1 (53.2, 59.2)	1.29 (1.20, 1.39)
		-0.68 - 0.00 (26-50th)	480/948	49.6 (42.2, 58.3)	1.14 (1.04, 1.26)
		0.01 - 0.67 (51-75th)	469/1072	43.3 (39.9, 47.1)	1
		> 0.67 (>75th)	238/675	35.0 (30.0, 40.9)	0.81 (0.68, 0.96)
	Normal	<= -0.67 (≤25th)	865/1846	46.3 (42.6, 50.3)	1.44 (1.35, 1.54)
		-0.68 - 0.00 (26-50th)	818/2061	38.0 (35.1, 41.2)	1.18 (1.09, 1.28)
		0.01 - 0.67 (51-75th)	861/2557	32.1 (28.4, 36.4)	1
		> 0.67 (>75th)	569/2142	25.4 (22.5, 28.7)	0.79 (0.73, 0.86)
	Overweight	<= -0.67 (≤25th)	127/435	27.3 (24.3, 30.7)	1.34 (1.16, 1.55)
		-0.68 - 0.00 (26-50th)	89/354	24.0 (21.8, 26.4)	1.18 (1.04, 1.34)
		0.01 - 0.67 (51-75th)	109/478	20.4 (18.2, 22.9)	1
		> 0.67 (>75th)	101/581	16.2 (13.5, 19.4)	0.79 (0.67, 0.94)
	Obese	<= -0.67 (≤25th)	21/102	19.3 (11.7, 31.8)	1.57 (0.90, 2.74)
		-0.68 - 0.00 (26-50th)	15/82	17.5 (10.5, 29.3)	1.42 (0.69, 2.93)
		0.01 - 0.67 (51-75th)	14/95	12.3 (6.7, 22.7)	1
		> 0.67 (>75th)	18/155	9.9 (7.0, 14.0)	0.80 (0.37, 1.72)
Small-for-gestational-age <3rd	Underweight	<= -0.67 (≤25th)	207/655	29.7 (26.8, 32.8)	1.50 (1.33, 1.69)
		-0.68 - 0.00 (26-50th)	258/948	26.0 (21.2, 31.9)	1.31 (1.06, 1.63)
		0.01 - 0.67 (51-75th)	218/1072	19.8 (16.7, 23.5)	1
		> 0.67 (>75th)	101/675	14.6 (12.2, 17.3)	0.74 (0.66, 0.83)
	Normal	<= -0.67 (≤25th)	429/1846	22.2 (19.9, 24.7)	1.51 (1.39, 1.63)
		-0.68 - 0.00 (26-50th)	372/2061	16.6 (13.4, 20.6)	1.13 (0.96, 1.32)
		0.01 - 0.67 (51-75th)	412/2557	14.7 (12.8, 16.9)	1
		> 0.67 (>75th)	225/2142	9.6 (7.3, 12.5)	0.65 (0.56, 0.76)
	Overweight	<= -0.67 (≤25th)	51/435	9.1 (6.1, 13.5)	1.31 (0.76, 2.26)
		-0.68 - 0.00 (26-50th)	33/354	7.2 (5.3, 9.7)	1.05 (0.76, 1.44)
		0.01 - 0.67 (51-75th)	46/478	6.9 (5.4, 8.8)	1
		> 0.67 (>75th)	39/581	5.6 (4.5, 7.1)	0.82 (0.65, 1.03)
	Obese	<= -0.67 (≤25th)	11/102	9.1 (5.3, 15.5)	1.26 (0.51, 3.14)

		-0.68 - 0.00 (26-50th)	6/82	7.0 (3.3, 15.2)	0.98 (0.47, 2.03)
		0.01 - 0.67 (51-75th)	8/95	7.2 (4.2, 12.2)	1
		> 0.67 (>75th)	13/155	6.5 (4.9, 8.5)	0.90 (0.52, 1.56)

[a] Adjusted for maternal age, gestational age at enrollment, maternal height, maternal BMI, parous, and previous preterm birth

Figures

Figure B1. Observed (circles) and predicted values (curves) for maternal weight gain in the reference sub-population (AMANHI-Bangladesh).

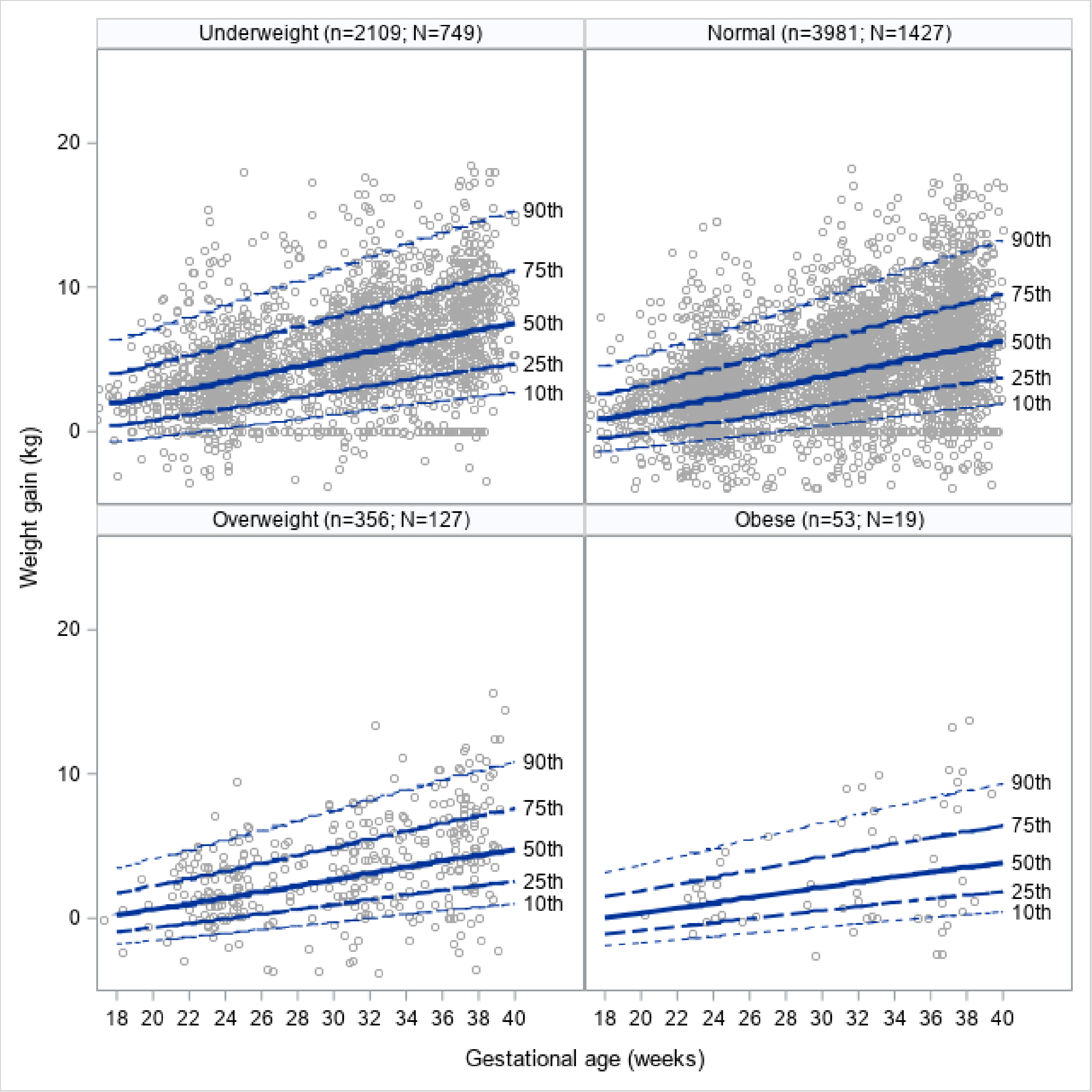

Figure B2. Observed (circles) and predicted values (curves) for maternal weight gain in the reference sub-population (AMANHI-Pakistan).

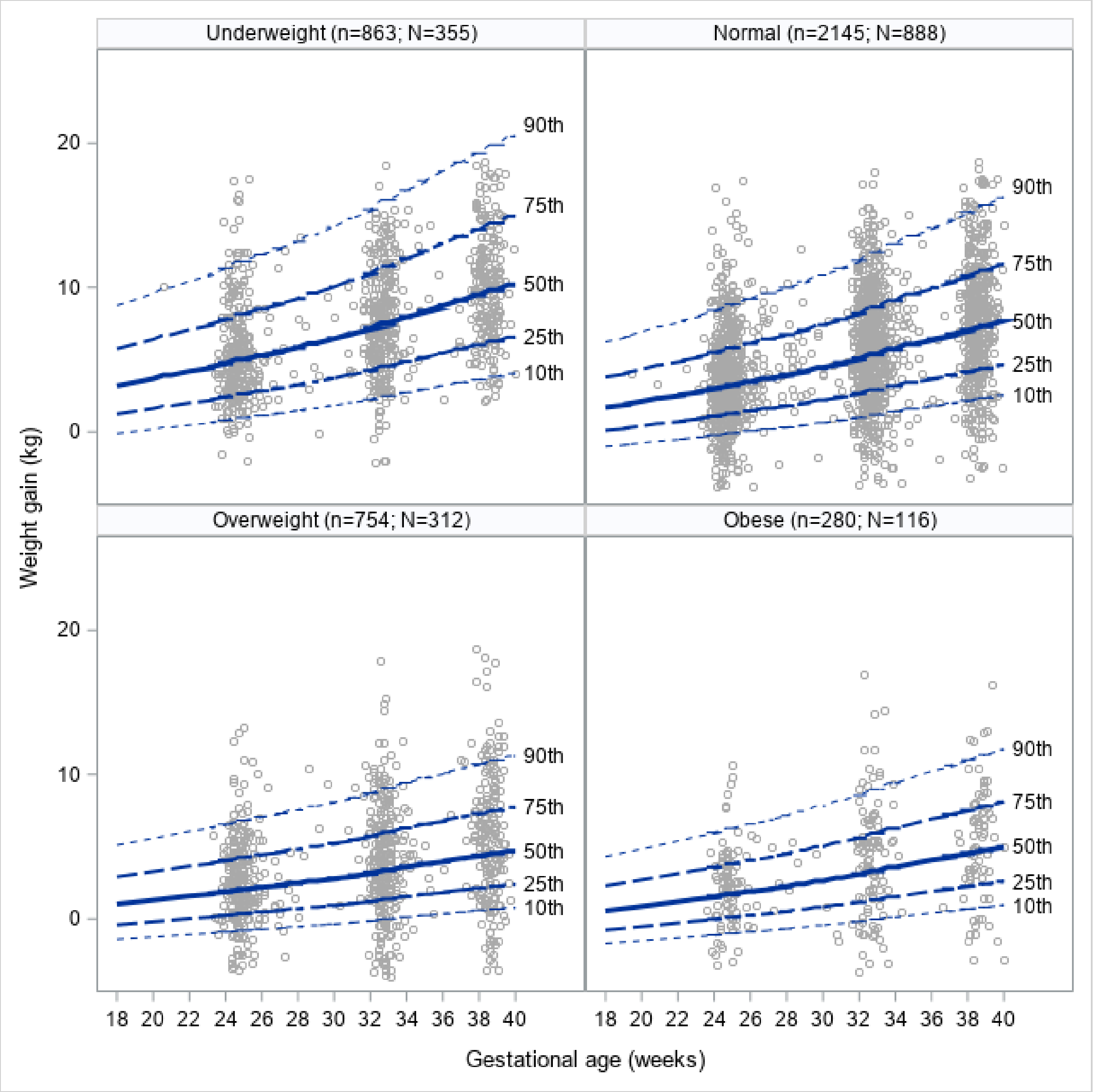

Figure B3. Observed (circles) and predicted values (curves) for maternal weight gain in the reference sub-population (PreSSMat).

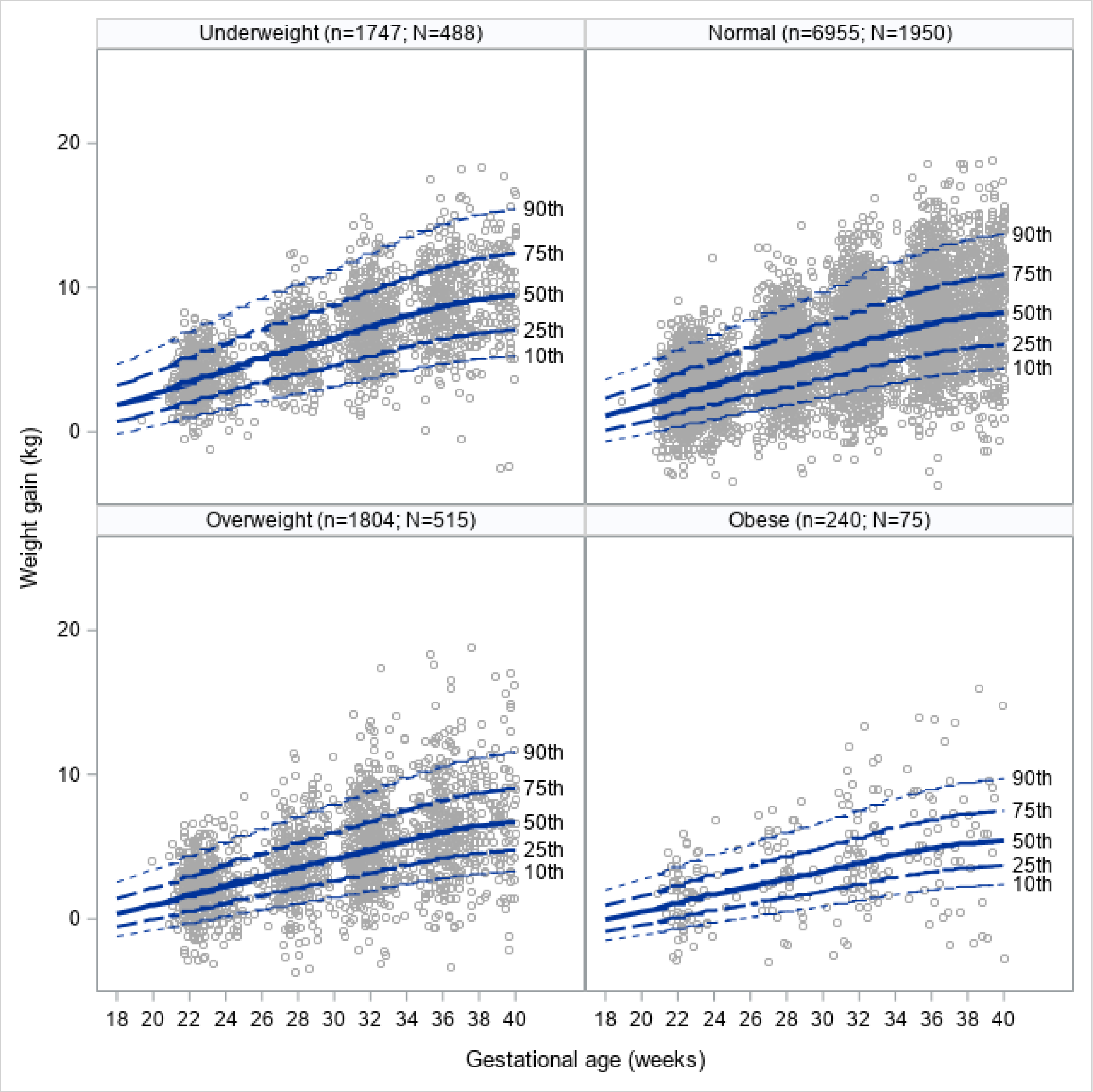

Figure B4. Observed (circles) and predicted values (curves) for maternal weight gain in the reference sub-population (GARBH-Ini).

(Plot for overweight group truncated at 40 weeks due to small sample size, n=36 observations, beyond 40 weeks)

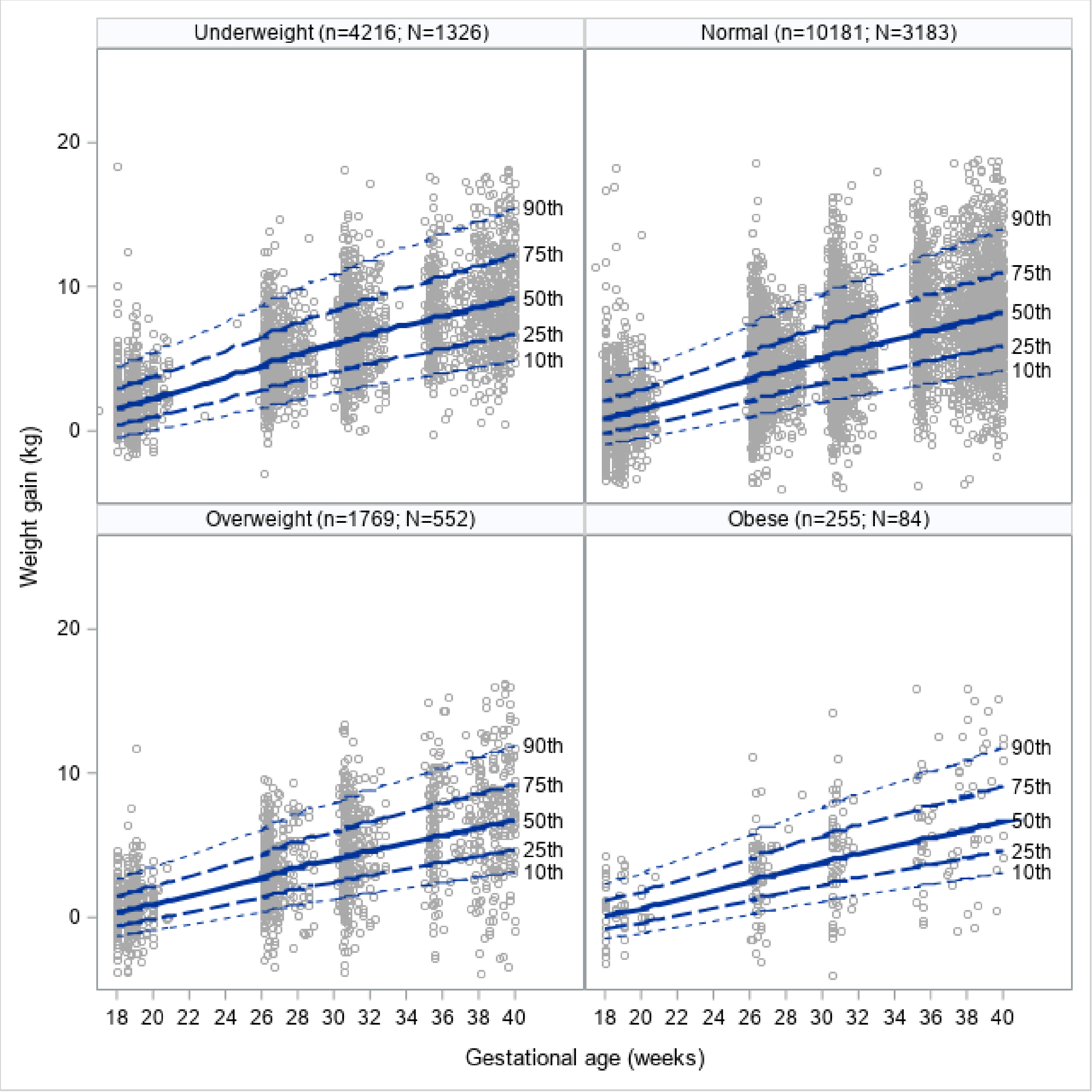

Figure B5. Observed (circles) and predicted values (curves) for maternal weight gain in the reference sub-population (ZAPPS).

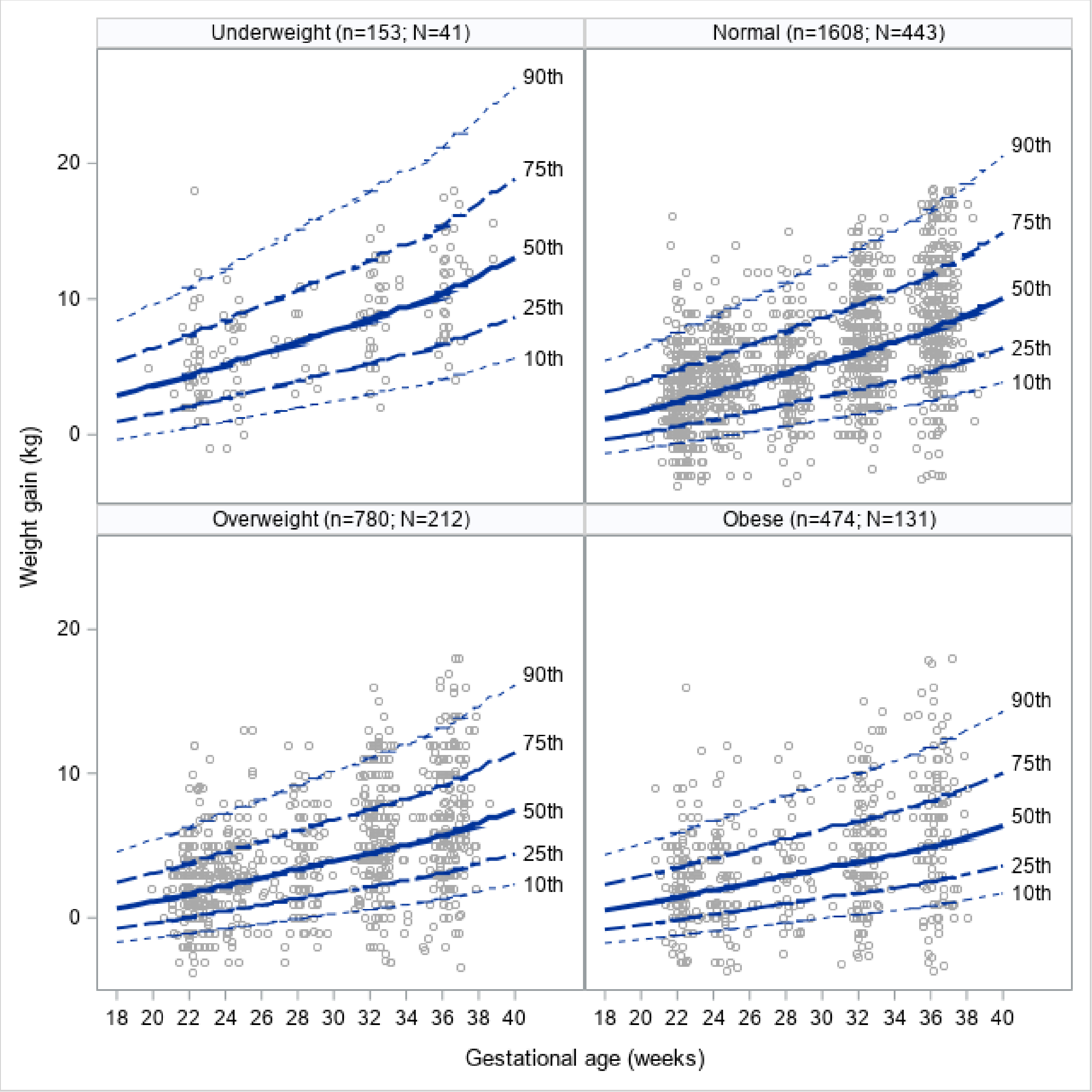

Figure B6. Non-linear association between probability of preterm birth and GWG z-score, stratified by baseline BMI.

Adjusted for: maternal age, gestational age at enrollment, maternal height, previous preterm birth, parous, and maternal years of education.

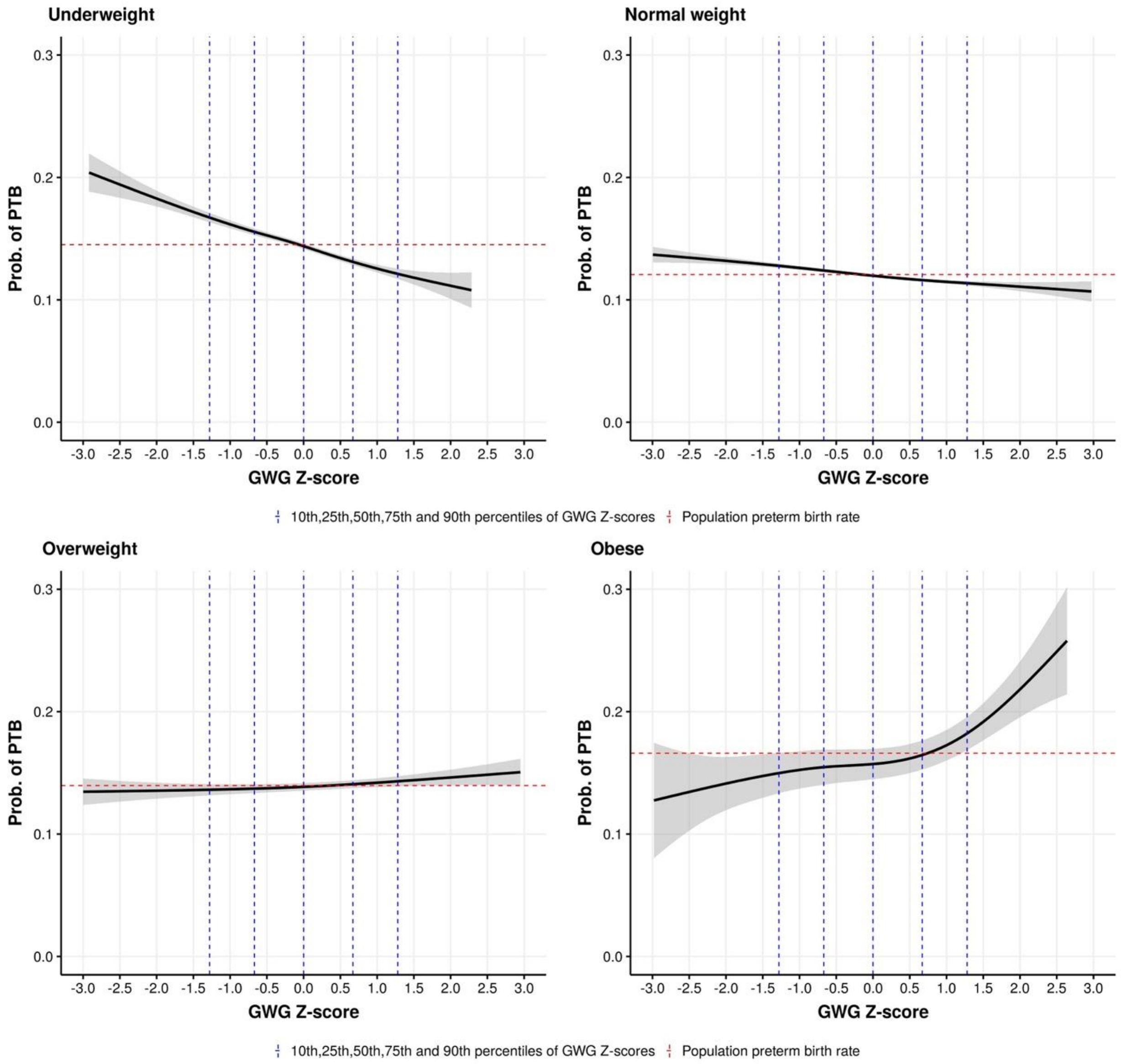

Figure B7. Cohort-specific, non-linear association between probability of preterm birth and GWG z-score, stratified by baseline BMI

Adjusted for: maternal age, gestational age at enrollment, maternal height, previous preterm birth, parous, and maternal years of education.

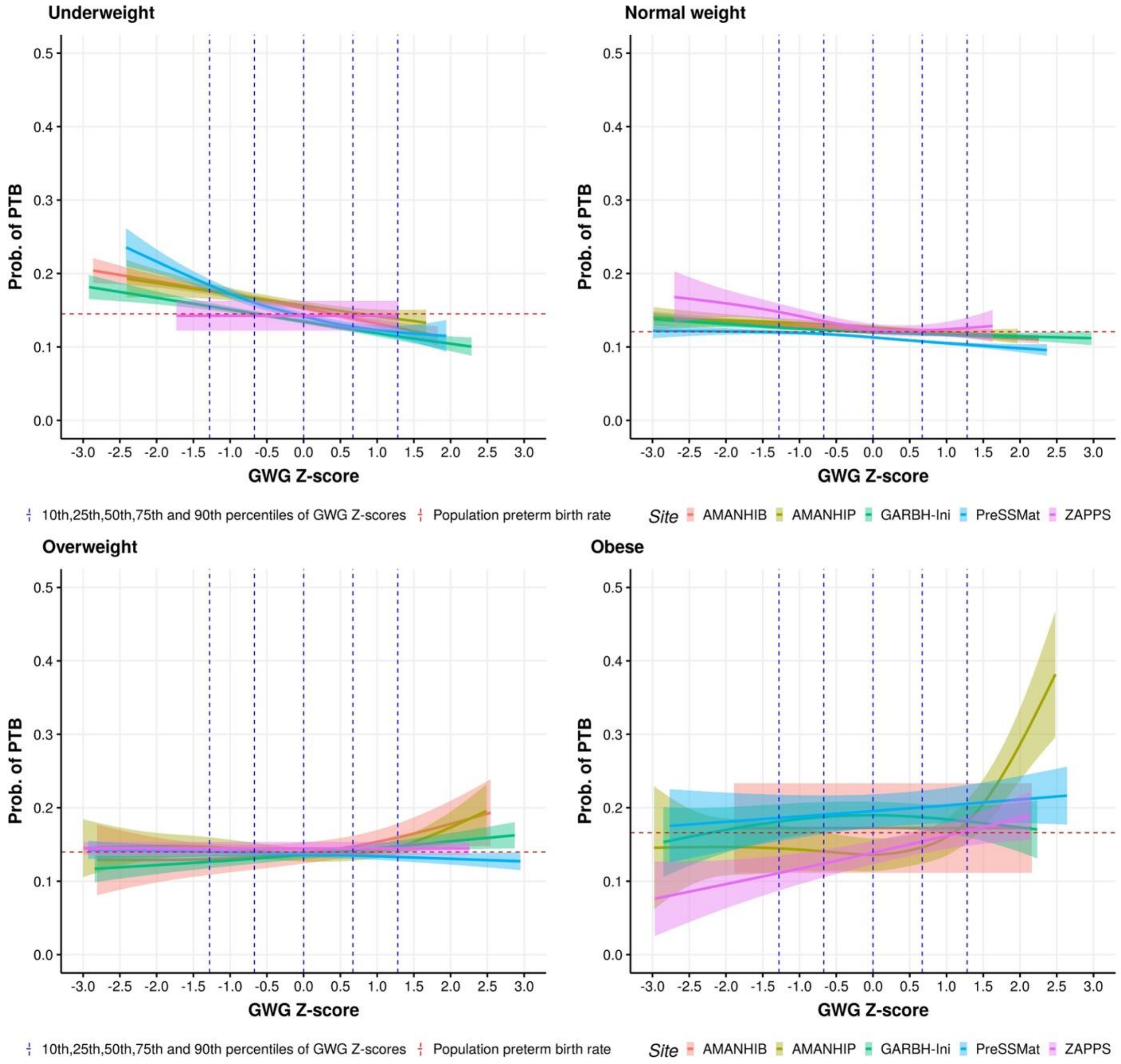

Figure B8. Non-linear association between probability of low birthweight and GWG z-score, stratified by baseline BMI

Adjusted for: maternal age, gestational age at enrollment, maternal height, parous, and previous stillbirth.

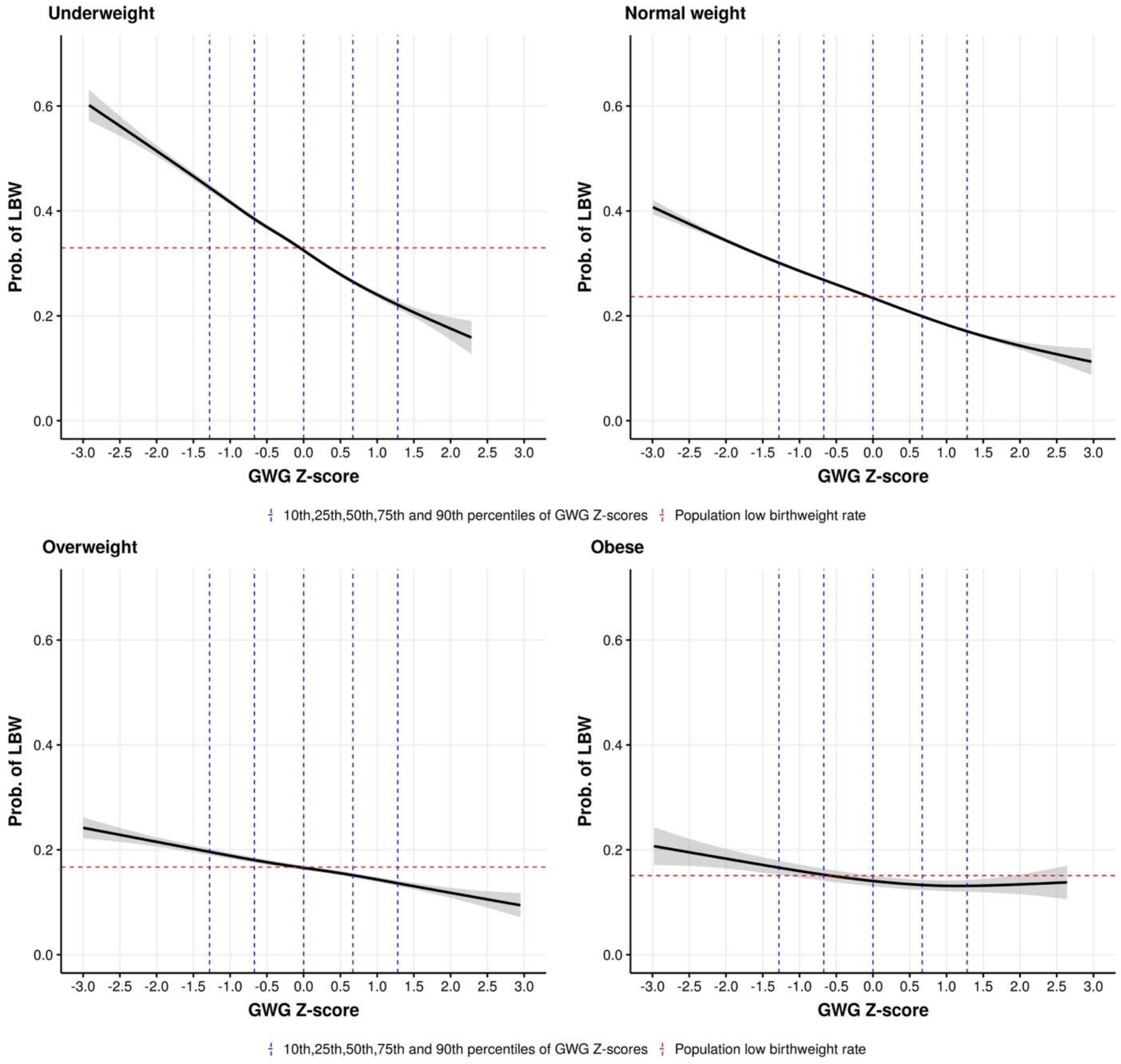

Figure B9. Cohort-specific non-linear association between probability of low birthweight and GWG z-score, in the overall study population and stratified by baseline BMI

Adjusted for: maternal age, gestational age at enrollment, maternal height, parous, and previous preterm birth.

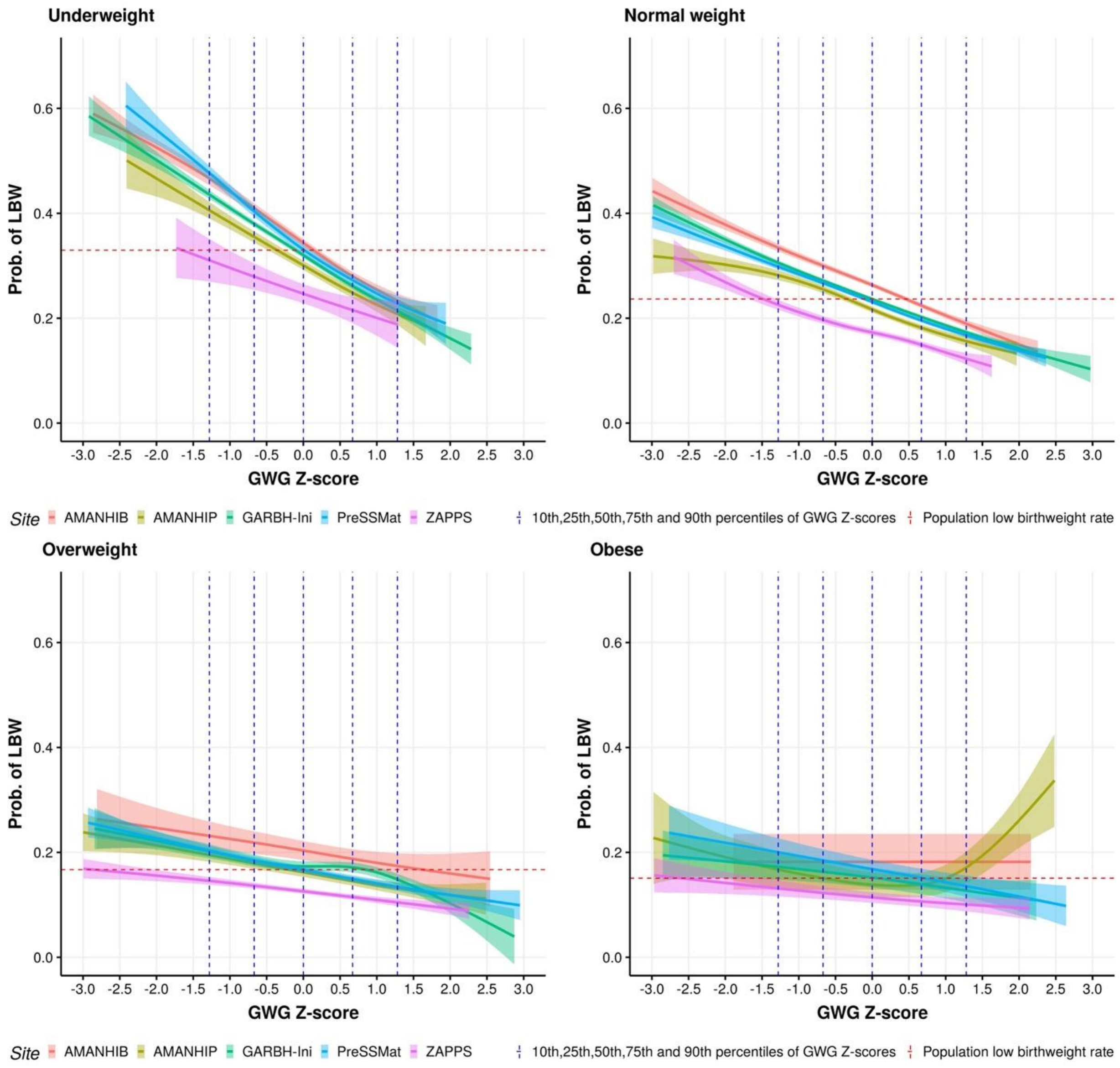

Figure B10. Non-linear association between probability of SGA (birthweight <10th centile) and GWG z-score, in the overall study population and stratified by baseline BMI.

Adjusted for: maternal age, gestational age at enrollment, maternal height, parous, and previous preterm birth.

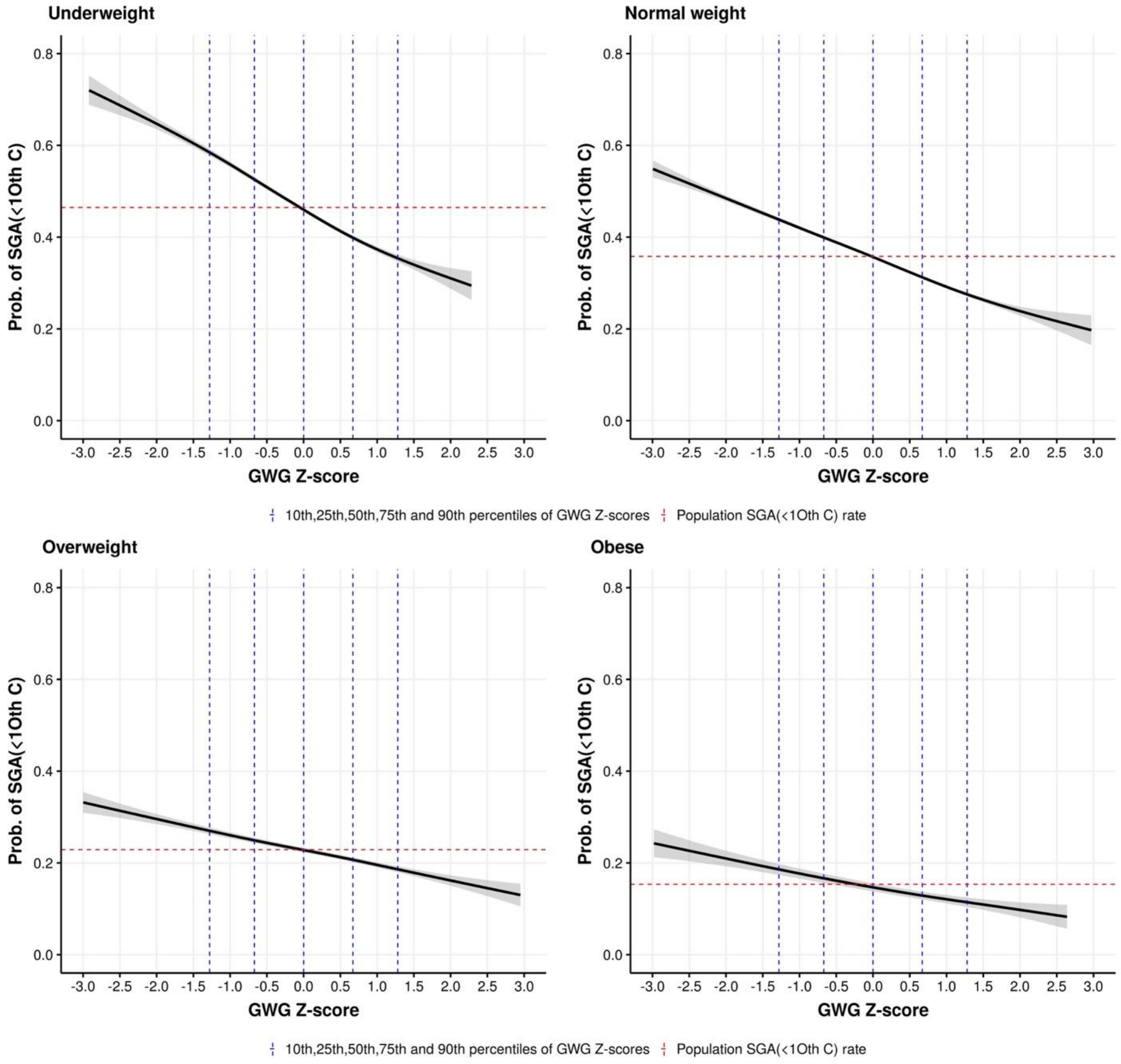

Figure B11. Cohort-specific, non-linear association between probability of SGA (birthweight <10th centile) and GWG z-score, stratified by baseline BMI.

Adjusted for: maternal age, gestational age at enrollment, maternal height, parous, and previous preterm birth.

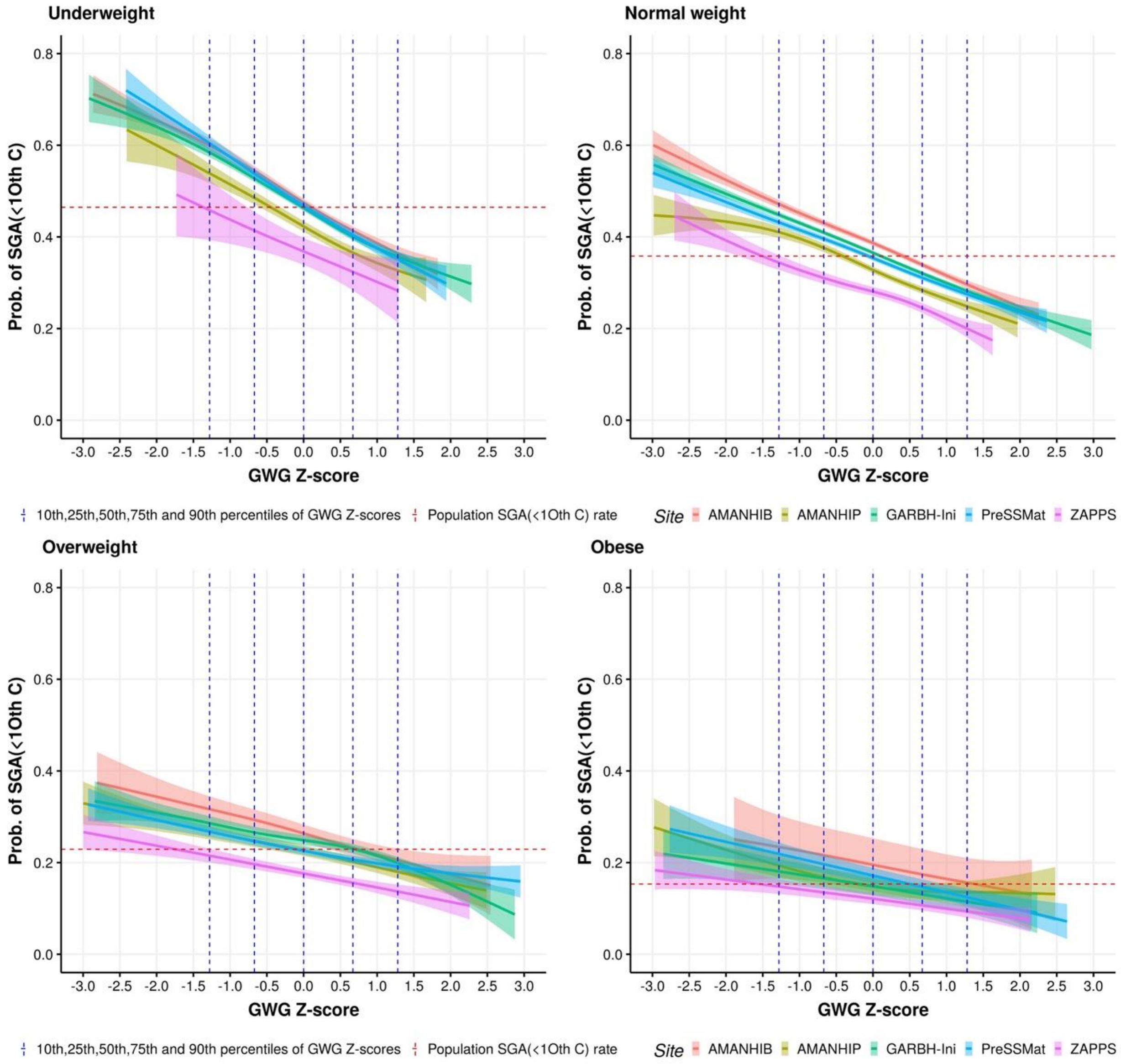

Figure B12. Non-linear association between probability of SGA (birthweight <3rd centile) and GWG z-score, stratified by baseline BMI.

Adjusted for: maternal age, gestational age at enrollment, maternal height, parous, and previous preterm birth.

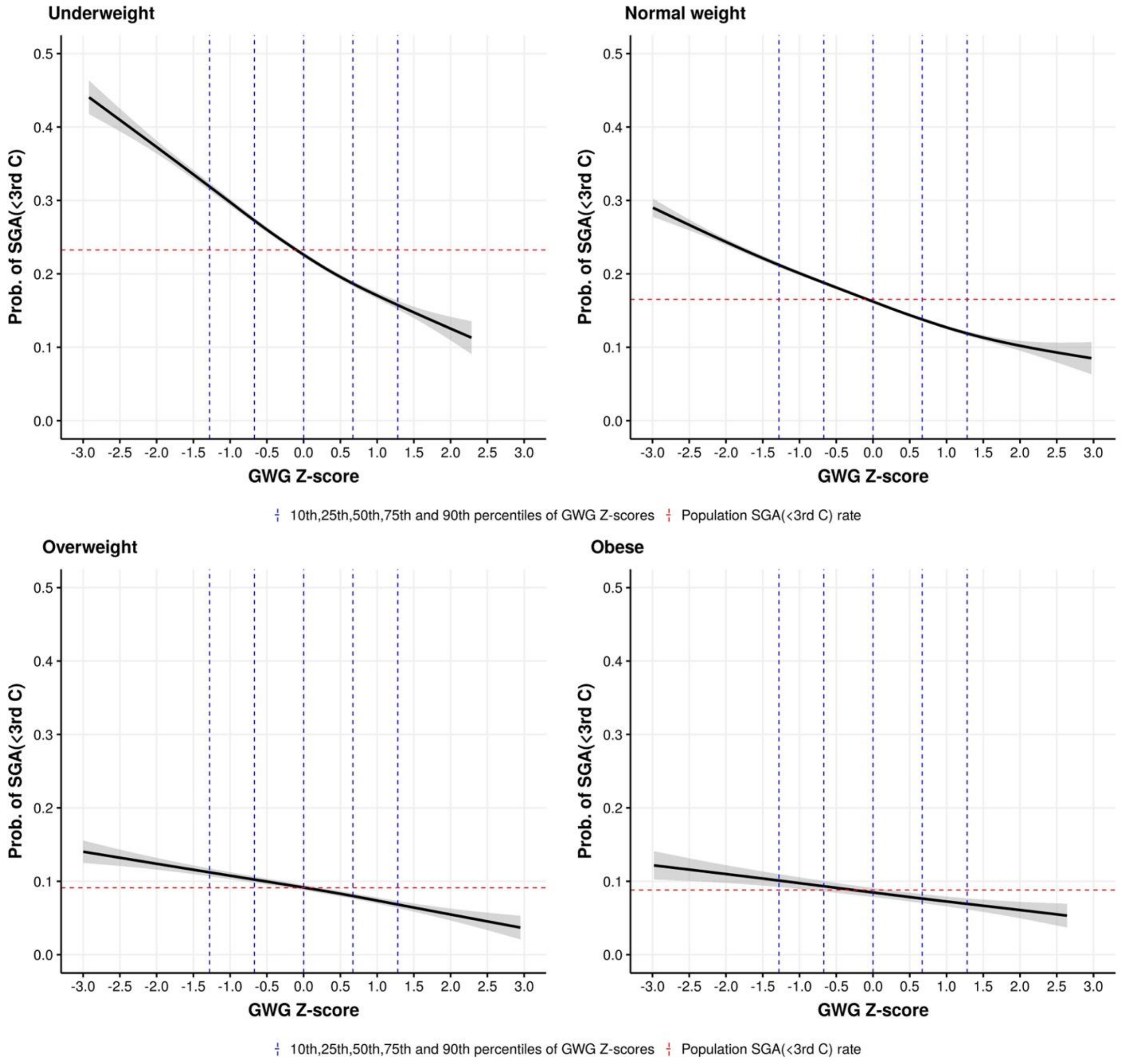

Figure B13. Cohort-specific, non-linear association between probability of SGA (birthweight <3rd centile) and GWG z-score, stratified by baseline BMI.

Adjusted for: maternal age, gestational age at enrollment, maternal height, parous, and previous preterm birth.

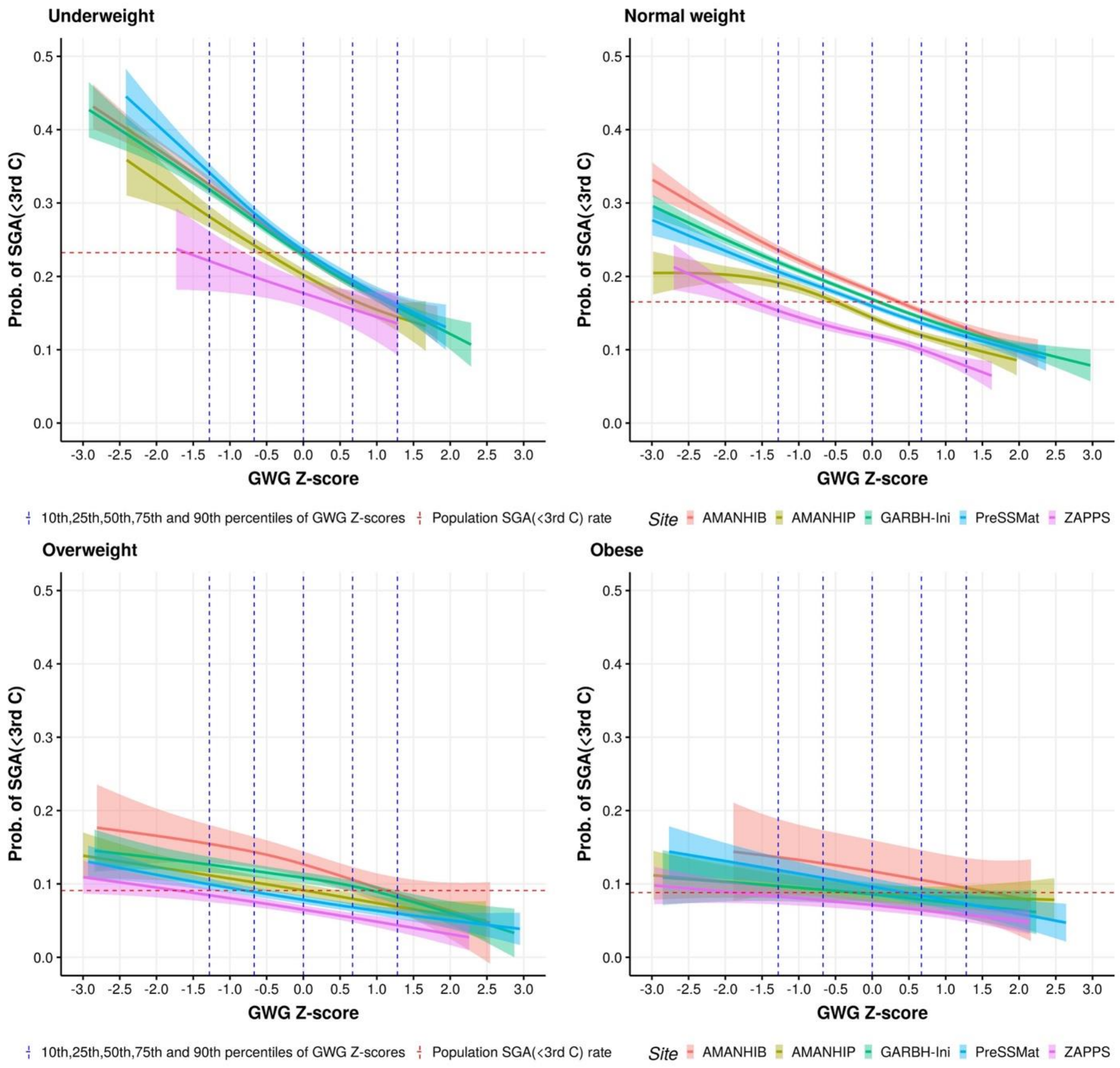

APPENDIX C – RESULTS BEFORE MULTIPLE IMPUTATION OF MISSING DATA

Table C1. Study population characteristics and outcomes, overall and by cohort. Pre-imputation.

	Overall (N=15286)		4: AMANHI-Bangladesh (N=2786)		3: AMANHI-Pakistan (N= 2025)		2: PreSSMat (N= 3456)		6: GARBH-Ini (N= 6004)		1: ZAPPS (N = 1015)	
	N	% (n) or median (IQR)	N	% (n) or median (IQR)	N	% (n) or median (IQR)	N	% (n) or median (IQR)	N	% (n) or median (IQR)	N	% (n) or median (IQR)
Maternal age (years)	15266	24.0 (21.0, 28.0)	2786	23.0 (20.0, 26.0)	2025	26.0 (23.0, 30.0)	3455	25.0 (21.0, 29.0)	6004	23.0 (21.0, 26.0)	996	27.0 (23.0, 32.0)
Gestational age at enrollment (Wks)	15286	13.0 (11.0, 16.0)	2786	13.4 (11.1, 16.6)	2025	13.4 (10.7, 16.7)	3456	12.1 (11.4, 13.6)	6004	13.1 (9.6, 16.0)	1015	16.3 (13.4, 18.4)
Gestational age at first maternal weight (Wks)	15286	12.9 (10.9, 15.9)	2786	12.4 (9.9, 15.7)	2025	13.4 (10.7, 16.7)	3456	12.1 (11.4, 13.6)	6004	13.1 (9.6, 16.1)	1015	16.3 (13.6, 18.4)
Maternal weight at baseline (Kg)	15286	48.3 (42.7, 55.6)	2786	43.7 (40.0, 48.7)	2025	50.5 (44.5, 60.0)	3456	49.5 (44.4, 56.2)	6004	47.7 (42.6, 54.2)	1015	61.0 (54.0, 72.0)
Maternal height (cm)	15286	152.5 (148.6, 156.6)	2786	150.0 (146.2, 153.2)	2025	153.5 (150.0, 158.0)	3456	151.7 (148.2, 155.4)	6004	153.1 (149.2, 156.9)	1015	160.0 (156.0, 164.0)
BMI (Kg/m2)	15286	20.7 (18.6, 23.5)	2786	19.5 (18.0, 21.4)	2025	21.5 (18.7, 25.2)	3456	21.5 (19.4, 24.3)	6004	20.3 (18.4, 22.9)	1015	23.9 (21.4, 27.6)
Underweight	15286	23.1% (3536)	2786	33.0% (920)	2025	22.4% (453)	3456	15.9% (549)	6004	26.1% (1567)	1015	4.6% (47)
Normal	15286	60.0% (9178)	2786	60.9% (1696)	2025	51.6% (1044)	3456	64.0% (2212)	6004	61.3% (3678)	1015	54.0% (548)
Overweight	15286	13.4% (2048)	2786	5.3% (148)	2025	19.0% (384)	3456	17.3% (598)	6004	10.8% (649)	1015	26.5% (269)
Obese	15286	3.4% (524)	2786	0.8% (22)	2025	7.1% (144)	3456	2.8% (97)	6004	1.8% (110)	1015	14.9% (151)
Parous (1+ previous delivery)	15286	60.5% (9253)	2786	64.2% (1790)	2025	75.9% (1537)	3456	63.0% (2176)	6004	50.6% (3038)	1015	70.1% (712)
Previous preterm birth[a]	9115	11.0% (1006)	1748	5.5% (97)	1528	9.2% (141)	2176	7.6% (166)	2951	11.0% (325)	712	38.9% (277)
Previous stillbirth[a]	9182	7.5% (693)	1790	11.7% (210)	1537	8.3% (128)	2176	4.1% (90)	3029	5.7% (174)	650	14.0% (91)
Maternal years of education	15273	10.0 (6.0, 13.0)	2783	7.0 (5.0, 9.0)	2025	0.0 (0.0, 8.0)	3455	8.0 (5.0, 10.0)	6004	14.0 (12.0, 15.0)	1006	12.0 (9.0, 12.0)
Chronic Hypertension	15176	1.9% (287)	2777	0.2% (6)	2007	5.7% (114)	3410	0.9% (32)	5998	0.3% (19)	984	11.8% (116)
Diabetes	15164	0.4% (62)	2776	0.3% (8)	2009	0.8% (17)	3373	0.4% (15)	6004	0.2% (12)	1002	1.0% (10)
Total maternal weight measurements	15286	4.0 (3.0, 5.0)	2786	4.0 (3.0, 4.0)	2025	3.0 (3.0, 4.0)	3456	5.0 (4.0, 5.0)	6004	4.0 (3.0, 5.0)	1015	5.0 (4.0, 5.0)
Gestational age at last maternal weight	15286	36.4 (32.4, 38.6)	2786	36.6 (34.0, 37.6)	2025	37.7 (32.7, 38.6)	3456	36.0 (33.0, 38.6)	6004	37.1 (30.7, 39.4)	1015	36.1 (35.4, 36.6)
Total GWG (Kg)	15286	6.8 (4.2, 9.4)	2786	6.1 (3.7, 8.5)	2025	6.5 (3.8, 9.5)	3456	7.3 (5.1, 9.8)	6004	6.7 (4.1, 9.5)	1015	7.0 (4.0, 10.0)
Total GWG, participants w/ both 2nd&3rd trimester weight[b]	12938	7.2 (4.8, 9.8)	2453	6.3 (3.9, 8.7)	1612	7.0 (4.3, 9.9)	3224	7.6 (5.4, 10.0)	4750	7.4 (5.1, 10.0)	899	7.4 (4.6, 10.6)
2nd trimester GWG (Kg)[b]	12938	3.4 (1.9, 5.2)	2453	2.9 (1.5, 4.6)	1612	3.5 (1.8, 5.5)	3224	3.5 (2.1, 5.1)	4750	3.7 (2.1, 5.5)	899	3.0 (1.0, 5.2)
3rd trimester GWG (Kg)[b]	12938	7.1 (4.8, 9.7)	2453	6.3 (3.9, 8.7)	1612	7.0 (4.3, 9.9)	3224	7.5 (5.3, 10.0)	4750	7.3 (5.0, 9.8)	899	7.4 (4.5, 10.6)
GWG Z-score percentile[c]	15286	53.7 (29.1, 74.8)	2786	55.9 (32.1, 73.8)	2025	55.1 (30.6, 73.4)	3456	53.6 (27.7, 76.4)	6004	52.4 (27.9, 75.1)	1015	52.3 (28.1, 74.5)
GWG Z-score percentile (normal BMI)[d]	8383	53.9 (28.7, 74.4)	1681	56.9 (31.8, 75.3)	1043	55.2 (32.0, 71.4)	2022	53.4 (27.2, 76.0)	3089	52.4 (27.8, 74.2)	548	52.3 (28.1, 71.3)
IG-21 GWG Z-score percentile (normal BMI)[d,e]	8383	9.8 (1.7, 29.2)	1681	4.4 (0.4, 17.7)	1043	9.3 (1.2, 27.3)	2022	13.4 (3.1, 34.2)	3089	11.1 (2.4, 29.6)	548	14.6 (1.9, 43.3)
IOM adequacy ratio[f]	15286	60.9 (40.7, 84.4)	2786	51.4 (31.8, 70.3)	2025	63.2 (38.1, 88.9)	3456	67.6 (48.5, 89.8)	6004	59.6 (40.6, 82.5)	1015	74.5 (42.9, 111.4)
IOM adequacy ratio (normal BMI)[d]	8383	58.8 (38.6, 79.9)	1681	50.6 (30.2, 70.6)	1043	59.7 (36.7, 80.2)	2022	64.9 (46.2, 84.9)	3089	57.7 (37.4, 77.8)	548	67.6 (42.0, 92.8)
Gestational age at delivery (Wks)	15286	39.1 (38.0, 40.0)	2786	39.1 (38.0, 40.0)	2025	39.0 (37.7, 39.9)	3456	39.0 (38.0, 39.9)	6004	39.1 (38.0, 40.0)	1015	39.6 (38.4, 40.4)
Spontaneous labor	13144	79.9% (10507)	2786	86.5% (2410)	1941	82.9% (1609)	3455	52.7% (1820)	4086	96.5% (3941)	876	83.0% (727)
Cesarean delivery	15198	26.5% (4030)	2786	13.5% (376)	1967	17.3% (341)	3456	47.4% (1639)	6004	24.2% (1454)	985	22.3% (220)
Preterm birth < 37 weeks	15286	13.0% (1993)	2786	13.1% (364)	2025	15.3% (309)	3456	11.8% (407)	6004	13.2% (790)	1015	12.1% (123)
Preterm birth < 32 weeks	15286	1.4% (217)	2786	1.3% (36)	2025	1.1% (22)	3456	0.8% (28)	6004	1.7% (101)	1015	3.0% (30)
Birth weight	13459	2800 (2500, 3100)	2238	2700 (2400, 3000)	1717	2780 (2505, 3050)	2836	2850 (2570, 3130)	5669	2763 (2497, 3043)	999	3100 (2800, 3390)
Birthweight <2500 g	13459	23.1% (3114)	2238	29.6% (663)	1717	23.0% (395)	2836	17.9% (509)	5669	25.1% (1425)	999	12.2% (122)
Birthweight <1500 g	13459	1.1% (152)	2238	0.6% (14)	1717	0.4% (7)	2836	0.6% (17)	5669	1.6% (90)	999	2.4% (24)
SGA <10th centile	13155	35.7% (4693)	2238	43.4% (972)	1715	34.7% (595)	2836	29.9% (849)	5368	39.1% (2100)	998	17.7% (177)
SGA <3rd centile	13155	16.4% (2154)	2238	21.7% (485)	1715	15.3% (262)	2836	12.0% (340)	5368	18.5% (993)	998	7.4% (74)
Stillbirth	15286	2.6% (396)	2786	3.7% (102)	2025	3.2% (65)	3456	1.5% (52)	6004	2.3% (141)	1015	3.5% (36)

Abbreviations. BMI, body mass index; IG-21, INTERGROWTH-21st; IOM, Institute of Medicine GWG, Gestational weight gain

[a] Among parous participants.

[b] Among participants with available 2nd *and* 3rd trimester weight gain

[c] Weight-gain-for-gestational-age Z-score based on BMI- and cohort-specific reference GWG values estimated from the study sub-population

[d] Restricted to normal BMI participants with final weight measurement at 14-40 weeks of gestation (the target population of INTERGROWTH-21 weight-gain-for-gestational-age standards) (https://doi.org/10.1136/bmj.i555)

[e] Weight-gain-for-gestational-age Z-score based on INTERGROWTH-21 weight-gain-for-gestational-age standards (https://doi.org/10.1136/bmj.i555)

[f] Ratio of (observed/recommended weight gain)*100. Recommended weight gain calculated as: 1st trimester recommended weight gain + [2nd & 3rd trimester recommended weekly weight gain rate*(final gestational age weeks-13)]. We assumed a 1st trimester weight gain of 2kg for underweight/normal BMI participants and of 0.5kg for overweight/obese. We assumed a 2nd & 3rd trimester weight gain rate of 0.51, 0.42, 0.28, 0.22 kg/week for underweight, normal, overweight, and obese respectively. IOM 2009 guidelines: https://doi.org/10.17226/12584.

Table C2. Unadjusted risk of primary outcomes by study population characteristics. Pre-imputation.

		Preterm birth <37 weeks			Low birthweight <2500 Kg			SGA < 10th centile			SGA < 3rd centile		
		N	Risk, % (95% CI)	RR (95% CI)	N	Risk, % (95% CI)	RR (95% CI)	N	Risk, % (95% CI)	RR (95% CI)	N	Risk, % (95% CI)	RR (95% CI)
Weight gain z-score group (percentile range)	<= -0.67 (≤25th)	3315	14.0 (12.3, 16.0)	**1.16 (1.02, 1.32)**	2874	31.5 (26.4, 37.7)	**1.51 (1.40, 1.62)**	2811	44.9 (39.5, 51.0)	**1.34 (1.27, 1.42)**	2803	22.0 (18.7, 26.0)	**1.44 (1.32, 1.57)**
	-0.68 - 0.00 (26-50th)	3689	14.0 (12.8, 15.3)	**1.16 (1.05, 1.28)**	3261	26.7 (22.5, 31.6)	**1.27 (1.12, 1.44)**	3193	40.7 (35.1, 47.2)	**1.22 (1.15, 1.28)**	3187	19.1 (14.9, 24.6)	**1.25 (1.08, 1.46)**
	0.01 - 0.67 (51-75th)	4473	12.1 (11.5, 12.7)	1	3954	20.9 (17.6, 24.9)	1	3854	33.5 (28.5, 39.3)	1	3847	15.3 (12.4, 18.9)	1
	> 0.67 (>75th)	3809	12.4 (10.7, 14.4)	1.03 (0.87, 1.21)	3370	15.1 (12.2, 18.8)	**0.72 (0.66, 0.79)**	3297	25.6 (21.4, 30.6)	**0.77 (0.74, 0.79)**	3292	10.2 (7.6, 13.6)	**0.66 (0.61, 0.73)**
Maternal age, years	<20	2166	13.3 (10.9, 16.3)	1.04 (0.85, 1.28)	1845	27.5 (20.9, 36.2)	1.14 (0.95, 1.37)	1818	41.4 (35.2, 48.8)	**1.10 (1.00, 1.20)**	1816	21.2 (16.4, 27.3)	**1.21 (1.04, 1.41)**
	20-24	5903	12.8 (11.7, 14.0)	1	5215	24.1 (20.9, 27.8)	1	5063	37.8 (33.0, 43.4)	1	5056	17.5 (14.8, 20.7)	1
	25-29	4569	12.9 (12.1, 13.8)	1.01 (0.92, 1.11)	4046	21.7 (18.6, 25.2)	**0.90 (0.88, 0.92)**	3952	33.9 (30.0, 38.4)	**0.90 (0.86, 0.93)**	3943	14.5 (12.0, 17.5)	**0.83 (0.78, 0.88)**
	30-34	1866	13.6 (12.5, 14.7)	**1.06 (1.02, 1.11)**	1657	20.9 (18.1, 24.1)	**0.87 (0.78, 0.96)**	1631	31.0 (26.2, 36.8)	**0.82 (0.73, 0.92)**	1628	13.6 (10.5, 17.6)	**0.78 (0.66, 0.91)**
	35+	762	14.3 (12.6, 16.3)	1.12 (0.92, 1.36)	677	18.8 (15.4, 22.9)	**0.78 (0.63, 0.96)**	672	26.3 (24.0, 28.9)	**0.70 (0.63, 0.77)**	667	13.4 (10.1, 17.7)	**0.77 (0.65, 0.90)**
Gestational age at enrollment	Lowest (5-10)	3737	16.5 (16.1, 16.9)	1	3344	25.2 (22.6, 28.0)	1	3261	33.7 (30.8, 36.9)	1	3261	15.2 (13.5, 17.1)	1
	Second (11-12)	3815	12.1 (11.0, 13.2)	**0.73 (0.67, 0.79)**	3271	21.5 (17.3, 26.7)	**0.86 (0.72, 1.02)**	3234	33.7 (27.4, 41.5)	1	3233	14.6 (10.6, 20.0)	0.96 (0.76, 1.21)
	Third (13-16)	3991	13.1 (12.0, 14.2)	**0.79 (0.73, 0.87)**	3510	24.1 (20.3, 28.5)	0.96 (0.82, 1.11)	3426	39.2 (33.4, 46.0)	**1.16 (1.02, 1.33)**	3420	19.0 (15.6, 23.3)	**1.25 (1.08, 1.45)**
	Highest (16-24)	3743	11.1 (10.4, 12.0)	**0.68 (0.62, 0.73)**	3334	21.8 (17.5, 27.0)	0.86 (0.70, 1.07)	3234	35.9 (28.9, 44.5)	1.06 (0.86, 1.32)	3215	16.5 (13.0, 21.0)	1.09 (0.88, 1.34)
Maternal height, quartiles	Lowest (100-148)	3825	14.9 (14.4, 15.5)	**1.26 (1.13, 1.39)**	3287	31.9 (28.1, 36.3)	**1.97 (1.63, 2.39)**	3222	46.0 (41.3, 51.3)	**1.70 (1.37, 2.11)**	3220	22.7 (19.2, 26.7)	**2.09 (1.64, 2.66)**
	Second (149-152)	3811	13.5 (12.5, 14.6)	**1.14 (1.02, 1.27)**	3331	24.7 (20.6, 29.4)	**1.52 (1.30, 1.78)**	3261	37.8 (33.4, 42.9)	**1.40 (1.17, 1.68)**	3260	17.5 (14.3, 21.4)	**1.61 (1.32, 1.96)**
	Third (153-156)	3829	11.9 (10.6, 13.4)	1.00 (0.95, 1.06)	3359	20.2 (17.0, 24.0)	**1.25 (1.12, 1.39)**	3271	32.3 (27.3, 38.2)	**1.20 (1.05, 1.36)**	3266	14.8 (11.9, 18.5)	**1.37 (1.21, 1.55)**
	Highest (157-190)	3821	11.9 (10.5, 13.4)	1	3482	16.2 (13.0, 20.2)	1	3401	27.0 (21.4, 34.1)	1	3383	10.9 (8.1, 14.5)	1
Body mass index (Kg/m2)	Underweight	3536	14.5 (12.8, 16.5)	**1.20 (1.08, 1.34)**	3082	31.6 (27.9, 35.8)	**1.42 (1.31, 1.54)**	2999	46.7 (43.1, 50.7)	**1.31 (1.23, 1.40)**	2998	22.9 (20.6, 25.6)	**1.43 (1.30, 1.57)**
	Normal	9178	12.1 (11.6, 12.6)	1.00 (1.00, 1.00)	8102	22.2 (19.4, 25.5)	1	7917	35.6 (31.6, 40.1)	1	7900	16.0 (13.4, 19.3)	1
	Overweight	2048	14.0 (12.9, 15.2)	1.16 (1.07, 1.25)	1819	15.1 (13.1, 17.5)	**0.68 (0.61, 0.76)**	1787	22.7 (19.7, 26.0)	**0.64 (0.59, 0.69)**	1782	8.7 (6.7, 11.2)	**0.54 (0.46, 0.63)**
	Obese	524	16.6 (12.3, 22.4)	**1.38 (1.01, 1.87)**	456	13.8 (9.6, 19.8)	**0.62 (0.45, 0.86)**	452	15.3 (11.4, 20.5)	**0.43 (0.34, 0.54)**	449	9.1 (7.3, 11.2)	**0.57 (0.44, 0.72)**
Parous (1+ previous delivery)	No	6033	11.5 (10.2, 13.0)	1.00 (1.00, 1.00)	5314	26.2 (21.9, 31.4)	1	5152	42.6 (37.4, 48.6)	1	5137	21.3 (17.8, 25.4)	1
	Yes	9253	14.1 (13.0, 15.1)	**1.22 (1.06, 1.41)**	8145	21.2 (18.1, 24.8)	**0.81 (0.71, 0.91)**	8003	31.2 (27.0, 36.0)	**0.73 (0.69, 0.78)**	7992	13.2 (10.7, 16.4)	**0.62 (0.57, 0.69)**
Previous preterm birth	No	14142	12.1 (11.2, 13.1)	1.00 (1.00, 1.00)	12443	22.7 (19.0, 27.2)	1	12164	36.2 (31.4, 41.7)	1	12139	16.6 (13.6, 20.4)	1
	Yes	1006	26.4 (22.7, 30.9)	**2.18 (1.83, 2.60)**	892	29.2 (22.2, 38.2)	1.28 (0.96, 1.71)	879	27.8 (21.7, 35.5)	**0.77 (0.63, 0.94)**	878	12.7 (9.3, 17.5)	**0.77 (0.59, 0.99)**
Previous stillbirth	No	14522	12.7 (12.0, 13.5)	1.00 (1.00, 1.00)	12801	23.1 (19.7, 27.1)	1	12512	35.8 (31.1, 41.3)	1	12488	14.6 (12.2, 17.5)	1
	Yes	693	20.2 (17.1, 23.9)	**1.59 (1.35, 1.87)**	589	26.5 (22.0, 31.9)	**1.15 (1.04, 1.27)**	578	34.4 (27.6, 42.9)	0.96 (0.84, 1.10)	578	17.2 (13.5, 22.1)	1.18 (0.94, 1.48)
Maternal years of education	Lowest (0-6)	3953	14.0 (13.0, 15.1)	**1.13 (1.05, 1.21)**	3340	24.9 (20.6, 29.9)	1.10 (0.91, 1.32)	3339	35.7 (30.9, 41.3)	0.97 (0.83, 1.14)	3335	16.4 (13.1, 20.6)	0.96 (0.76, 1.23)
	Second (7-10)	3999	12.7 (11.2, 14.3)	1.02 (0.91, 1.13)	3308	21.5 (15.5, 29.7)	0.95 (0.69, 1.30)	3307	34.1 (27.1, 42.9)	0.93 (0.74, 1.16)	3304	15.5 (10.7, 22.4)	0.91 (0.63, 1.31)
	Third (11-13)	3668	13.0 (11.4, 14.8)	1.04 (0.92, 1.18)	3342	23.6 (17.5, 31.8)	1.04 (0.82, 1.32)	3233	36.2 (28.7, 45.6)	0.98 (0.85, 1.15)	3219	16.7 (12.6, 22.2)	0.98 (0.83, 1.16)
	Highest (14-17)	3653	12.5 (12.3, 12.6)	1	3458	22.7 (21.4, 24.1)	1	3265	36.8 (33.9, 39.8)	1	3260	17.0 (15.2, 19.0)	1
Chronic Hypertension	No	14889	12.9 (12.1, 13.7)	1	13111	23.2 (19.7, 27.2)	1.00 (1.00, 1.00)	12810	35.9 (31.2, 41.4)	1	12786	16.5 (13.4, 20.1)	1
	Yes	287	21.6 (18.2, 25.7)	**1.67 (1.37, 2.04)**	250	22.8 (15.8, 32.9)	0.98 (0.72, 1.35)	249	24.9 (19.5, 31.8)	**0.69 (0.58, 0.83)**	248	12.5 (8.6, 18.1)	0.76 (0.58, 0.99)
Diabetes	No	15102	13.0 (12.2, 13.8)	1	13308	23.2 (19.8, 27.2)	1.00 (1.00, 1.00)	13004	35.8 (31.0, 41.4)	1	12980	16.4 (13.4, 20.2)	1
	Yes	62	32.3 (22.6, 46.1)	**2.48 (1.74, 3.54)**	50	18.0 (11.5, 28.2)	0.78 (0.54, 1.11)	50	28.0 (20.1, 39.1)	0.78 (0.54, 1.13)	49	14.0 (7.2, 27.2)	0.85 (0.38, 1.92)

Table C3. Risk ratios for the association between gestational weight gain Z-score and preterm birth (<37 weeks), stratified by maternal BMI group. Pre-imputation

BMI Group	Weight gain z-score group (percentile range)	Risk (n/N)	Adjusted Risk,[a] % (95% CI)	Adjusted Risk Ratio[a] (95% CI)
Underweight (n=42 missing)[b]	<= -0.67 (≤25th)	121/682	17.6 (13.0, 23.9)	1.44 (1.07, 1.93)
	-0.68 - 0.00 (26-50th)	146/991	13.7 (11.9, 15.7)	1.12 (1.00, 1.25)
	0.01 - 0.67 (51-75th)	146/1,112	12.3 (11.7, 12.8)	1
	> 0.67 (>75th)	95/709	12.1 (9.8, 14.9)	0.99 (0.80, 1.21)
Normal (n=98 missing)[b]	<= -0.67 (≤25th)	254/1,978	11.9 (11.2, 12.7)	1.11 (1.03, 1.19)
	-0.68 - 0.00 (26-50th)	292/2,177	12.7 (12.2, 13.2)	1.18 (1.08, 1.29)
	0.01 - 0.67 (51-75th)	302/2,681	10.8 (9.8, 11.8)	1
	> 0.67 (>75th)	251/2,244	10.8 (9.7, 11.9)	1.00 (0.84, 1.19)
Overweight (n=23 missing)[b]	<= -0.67 (≤25th)	67/487	12.3 (9.5, 15.9)	1.03 (0.70, 1.51)
	-0.68 - 0.00 (26-50th)	57/392	13.8 (12.5, 15.2)	1.15 (0.86, 1.53)
	0.01 - 0.67 (51-75th)	69/525	12.0 (9.5, 15.2)	1
	> 0.67 (>75th)	90/621	13.4 (11.6, 15.6)	1.12 (0.82, 1.51)
Obese (n=7 missing)[b]	<= -0.67 (≤25th)	19/127	14.2 (8.6, 23.5)	0.92 (0.44, 1.95)
	-0.68 - 0.00 (26-50th)	16/93	15.3 (10.1, 23.1)	1.00 (0.37, 2.69)
	0.01 - 0.67 (51-75th)	20/116	15.4 (7.9, 30.1)	1
	> 0.67 (>75th)	32/181	15.4 (11.7, 20.2)	1.00 (0.47, 2.10)

[a] Adjusted for maternal age, gestational age at enrollment, maternal height, maternal BMI, parous, previous preterm birth, and maternal years of education

[b] Number missing outcome and/or covariate data

Table C4. Risk ratios for the association between gestational weight gain Z-score and low birthweight (<2500 g), stratified by BMI group. Pre-imputation

BMI Group	Weight gain z-score group (percentile range)	Risk (n/N)	Adjusted Risk,[a] % (95% CI)	Adjusted Risk Ratio[a] (95% CI)
Underweight (n=459 missing)[b]	<= -0.67 (≤25th)	267/588	45.1 (41.1, 49.4)	1.76 (1.54, 2.02)
	-0.68 - 0.00 (26-50th)	311/872	34.3 (31.4, 37.6)	1.34 (1.17, 1.54)
	0.01 - 0.67 (51-75th)	264/996	25.6 (23.0, 28.4)	1.00 (1.00, 1.00)
	> 0.67 (>75th)	131/621	20.1 (17.2, 23.4)	0.78 (0.65, 0.94)
Normal (n=1128 missing)[b]	<= -0.67 (≤25th)	535/1,726	30.2 (28.1, 32.5)	1.57 (1.41, 1.74)
	-0.68 - 0.00 (26-50th)	484/1,939	23.7 (21.9, 25.6)	1.23 (1.10, 1.37)
	0.01 - 0.67 (51-75th)	481/2,366	19.3 (17.8, 20.9)	1.00 (1.00, 1.00)
	> 0.67 (>75th)	298/2,019	14.1 (12.7, 15.7)	0.73 (0.64, 0.84)
Overweight (n=246 missing)[b]	<= -0.67 (≤25th)	84/430	18.2 (15.0, 22.2)	1.45 (1.08, 1.94)
	-0.68 - 0.00 (26-50th)	61/352	16.2 (12.8, 20.5)	1.29 (0.94, 1.77)
	0.01 - 0.67 (51-75th)	67/470	12.6 (10.0, 15.7)	1.00 (1.00, 1.00)
	> 0.67 (>75th)	62/550	10.6 (8.3, 13.4)	0.84 (0.61, 1.16)
Obese (n=80 missing)[b]	<= -0.67 (≤25th)	20/108	16.5 (10.8, 25.3)	1.71 (0.88, 3.33)
	-0.68 - 0.00 (26-50th)	12/79	12.4 (7.0, 21.9)	1.29 (0.63, 2.65)
	0.01 - 0.67 (51-75th)	13/100	9.6 (5.5, 17.0)	1.00 (1.00, 1.00)
	> 0.67 (>75th)	18/157	9.1 (5.9, 13.9)	0.94 (0.48, 1.84)

[a] Adjusted for maternal age, gestational age at enrollment, maternal height, maternal BMI, parous, and previous stillbirth

[b] Number missing outcome and/or covariate data

Table C5. Risk ratios for the association between gestational weight gain Z-score and small for gestational age (<10th percentile), stratified by BMI group. Pre-imputation

BMI Group	Weight gain z-score group (percentile range)	Risk (n/N)	Adjusted Risk,[a] % (95% CI)	Adjusted Risk Ratio[a] (95% CI)
Underweight (n=574 missing)[b]	<= -0.67 (≤25th)	332/566	56.6 (55.2, 58.0)	1.33 (1.24, 1.42)
	-0.68 - 0.00 (26-50th)	425/839	49.3 (40.9, 59.5)	1.15 (1.03, 1.30)
	0.01 - 0.67 (51-75th)	413/957	42.7 (39.4, 46.2)	1
	> 0.67 (>75th)	213/600	35.2 (30.2, 41.2)	0.83 (0.68, 1.00)
Normal (n=1331 missing)[b]	<= -0.67 (≤25th)	775/1682	45.3 (42.0, 48.9)	1.45 (1.38, 1.54)
	-0.68 - 0.00 (26-50th)	755/1897	38.1 (35.9, 40.3)	1.22 (1.13, 1.31)
	0.01 - 0.67 (51-75th)	755/2310	31.2 (27.8, 35.0)	1
	> 0.67 (>75th)	509/1958	24.8 (22.0, 27.9)	0.80 (0.73, 0.87)
Overweight (n=279 missing)[b]	<= -0.67 (≤25th)	114/422	25.5 (22.6, 28.8)	1.27 (1.13, 1.43)
	-0.68 - 0.00 (26-50th)	88/350	23.8 (21.5, 26.3)	1.18 (1.08, 1.30)
	0.01 - 0.67 (51-75th)	102/455	20.1 (19.1, 21.1)	1
	> 0.67 (>75th)	96/542	16.6 (13.8, 19.9)	0.83 (0.71, 0.96)
Obese (n=78 missing)[b]	<= -0.67 (≤25th)	22/111	19.1 (12.6, 28.8)	1.84 (1.24, 2.74)
	-0.68 - 0.00 (26-50th)	16/77	18.6 (13.4, 25.8)	1.80 (1.14, 2.84)
	0.01 - 0.67 (51-75th)	12/101	10.3 (6.2, 17.1)	1
	> 0.67 (>75th)	19/157	10.2 (7.5, 13.9)	0.99 (0.50, 1.97)

[a] Adjusted for maternal age, gestational age at enrollment, maternal height, maternal BMI, parous, and previous preterm birth

[b] Number missing outcome and/or covariate data

Table C6. Risk ratios for the association between gestational weight gain Z-score and small for gestational age (<3rd percentile), stratified by BMI group. Pre-imputation

BMI Group	Weight gain z-score group (percentile range)	Risk (n/N)	Adjusted Risk,[a] % (95% CI)	Adjusted Risk Ratio[a] (95% CI)
Underweight (n=574 missing)[b]	<= -0.67 (≤25th)	179/566	29.7 (28.0, 31.5)	1.58 (1.35, 1.85)
	-0.68 - 0.00 (26-50th)	228/839	25.8 (20.3, 32.7)	1.37 (1.03, 1.82)
	0.01 - 0.67 (51-75th)	185/957	14.1 (12.3, 16.2)	1
	> 0.67 (>75th)	87/600	18.9 (15.7, 22.7)	1.00 (1.00, 1.00)
Normal (n=1331 missing)[b]	<= -0.67 (≤25th)	373/1682	21.1 (19.0, 23.3)	1.52 (1.44, 1.61)
	-0.68 - 0.00 (26-50th)	338/1897	16.3 (13.3, 19.9)	1.17 (1.01, 1.36)
	0.01 - 0.67 (51-75th)	352/2310	9.1 (6.9, 11.9)	1
	> 0.67 (>75th)	197/1958	13.9 (12.4, 15.5)	1.00 (1.00, 1.00)
Overweight (n=279 missing)[b]	<= -0.67 (≤25th)	47/422	8.8 (6.4, 12.2)	1.32 (0.87, 1.99)
	-0.68 - 0.00 (26-50th)	30/350	6.7 (4.8, 9.4)	1.00 (0.69, 1.45)
	0.01 - 0.67 (51-75th)	42/455	5.5 (4.5, 6.7)	1
	> 0.67 (>75th)	35/542	6.7 (6.0, 7.5)	1.00 (1.00, 1.00)
Obese (n=78 missing)[b]	<= -0.67 (≤25th)	13/111	9.9 (7.1, 13.9)	1.73 (0.98, 3.03)
	-0.68 - 0.00 (26-50th)	8/77	8.7 (5.6, 13.6)	1.51 (0.94, 2.45)
	0.01 - 0.67 (51-75th)	7/101	6.8 (5.2, 8.9)	1
	> 0.67 (>75th)	13/157	5.8 (3.9, 8.4)	1.00 (1.00, 1.00)

[a] Adjusted for maternal age, gestational age at enrollment, maternal height, maternal BMI, parous, and previous preterm birth

[b] Number missing outcome and/or covariate data

Table C7. Study population characteristics and outcomes, overall and by cohort. Pre-imputation. Before exclusion of participants without 2+ valid maternal weight measurements*

	Overall (N=16588)		AMANHI-Bangladesh (N=2837)		AMANHI-Pakistan (N=2245)		PreSSMat (N=3548)		GARBH-Ini (N=6863)		ZAPPS (N=1095)	
Characteristic	N	% (n) or median (IQR)	N	% (n) or median (IQR)	N	% (n) or median (IQR)	N	% (n) or median (IQR)	N	% (n) or median (IQR)	N	% (n) or median (IQR)
Maternal age (years)	16568	24.0 (21.0, 28.0)	2837	23.0 (20.0, 26.0)	2245	26.0 (23.0, 30.0)	3547	24.0 (21.0, 29.0)	6863	23.0 (21.0, 26.0)	1076	27.0 (23.0, 32.0)
Gestational age at enrollment (Wks)	16588	13.0 (10.9, 16.0)	2837	13.4 (11.1, 16.6)	2245	13.4 (10.7, 16.7)	3548	12.1 (11.4, 13.6)	6863	12.9 (9.3, 16.0)	1095	16.3 (13.3, 18.4)
Gestational age at first maternal weight measurement (Wks)	16587	12.9 (10.7, 15.9)	2837	12.4 (9.9, 15.7)	2245	13.4 (10.7, 16.7)	3548	12.1 (11.4, 13.6)	6862	12.9 (9.3, 16.0)	1095	16.3 (13.4, 18.4)
Maternal weight at baseline (Kg)	16585	48.3 (42.7, 55.7)	2836	43.8 (40.0, 48.8)	2244	50.5 (44.5, 60.0)	3548	49.5 (44.5, 56.3)	6862	47.5 (42.5, 54.0)	1095	61.1 (54.0, 72.0)
Maternal height (cm)	16588	152.5 (148.7, 156.6)	2837	150.0 (146.2, 153.2)	2245	153.6 (150.0, 158.0)	3548	151.8 (148.2, 155.4)	6863	153.0 (149.2, 156.8)	1095	160.0 (156.0, 164.0)
Body mass index (Kg/m2)	16586	20.7 (18.6, 23.5)	2836	19.5 (18.0, 21.5)	2244	21.5 (18.7, 25.2)	3548	21.6 (19.4, 24.3)	6863	20.3 (18.4, 22.8)	1095	23.9 (21.4, 27.6)
Underweight	16588	23.3% (3862)	2837	32.9% (932)	2245	22.0% (494)	3548	15.8% (562)	6863	26.6% (1823)	1095	4.7% (51)
Normal	16588	59.9% (9938)	2837	60.8% (1725)	2245	51.7% (1160)	3548	63.9% (2266)	6863	61.2% (4198)	1095	53.8% (589)
Overweight	16588	13.3% (2210)	2837	5.4% (153)	2245	19.0% (426)	3548	17.5% (622)	6863	10.5% (720)	1095	26.4% (289)
Obese	16588	3.5% (576)	2837	0.9% (26)	2245	7.3% (164)	3548	2.8% (98)	6863	1.8% (122)	1095	15.2% (166)
Parous	16588	59.8% (9924)	2837	64.2% (1821)	2245	75.9% (1703)	3548	62.8% (2228)	6863	49.7% (3408)	1095	69.8% (764)
Previous preterm birth	9771	11.2% (1090)	1778	5.7% (101)	1692	9.5% (161)	2228	7.5% (168)	3309	10.8% (357)	764	39.7% (303)
Previous stillbirth	9849	7.5% (739)	1821	11.8% (215)	1703	8.5% (145)	2228	4.2% (93)	3398	5.6% (190)	699	13.7% (96)
Maternal years of education	16573	10.0 (6.0, 14.0)	2834	7.0 (5.0, 9.0)	2245	0.0 (0.0, 8.0)	3547	8.0 (5.0, 10.0)	6863	14.0 (12.0, 15.0)	1084	12.0 (9.0, 12.0)
Chronic Hypertension	16466	1.9% (315)	2828	0.2% (6)	2218	6.0% (132)	3502	1.0% (35)	6857	0.3% (20)	1061	11.5% (122)
Diabetes	16455	0.4% (65)	2826	0.3% (8)	2220	0.9% (19)	3465	0.4% (15)	6863	0.2% (13)	1081	0.9% (10)
Total maternal weight measurements	16588	4.0 (3.0, 5.0)	2837	4.0 (3.0, 4.0)	2245	3.0 (3.0, 4.0)	3548	5.0 (4.0, 5.0)	6863	4.0 (3.0, 5.0)	1095	5.0 (4.0, 5.0)
Gestational age at delivery (Wks)	16588	39.1 (38.0, 40.0)	2837	39.1 (38.0, 40.0)	2245	39.0 (37.7, 39.9)	3548	39.0 (38.0, 39.9)	6863	39.1 (38.0, 40.0)	1095	39.6 (38.4, 40.4)
Spontaneous labor	13681	79.9% (10931)	2837	86.4% (2450)	2119	82.8% (1754)	3547	52.4% (1858)	4230	96.5% (4080)	948	83.2% (789)
Cesarean delivery	16458	26.3% (4327)	2837	13.6% (387)	2151	17.4% (374)	3548	47.7% (1693)	6863	23.9% (1642)	1059	21.8% (231)
Preterm birth < 37 weeks	16588	13.5% (2240)	2837	13.0% (370)	2245	16.6% (372)	3548	11.9% (422)	6863	13.6% (935)	1095	12.9% (141)
Preterm birth < 32 weeks	16588	1.7% (280)	2837	1.3% (37)	2245	1.6% (37)	3548	0.9% (32)	6863	1.9% (130)	1095	4.0% (44)
Birth weight	14465	2800 (2500, 3100)	2275	2700 (2400, 3000)	1845	2780 (2505, 3050)	2867	2850 (2570, 3135)	6409	2770 (2500, 3050)	1069	3100 (2800, 3400)
Birthweight <2500 Kg	14465	23.0% (3321)	2275	29.5% (670)	1845	23.5% (434)	2867	18.0% (517)	6409	24.5% (1570)	1069	12.2% (130)
Birthweight <1500 Kg	14465	1.2% (173)	2275	0.6% (14)	1845	0.5% (9)	2867	0.6% (18)	6409	1.6% (105)	1069	2.5% (27)
SGA <10th centile	14093	35.5% (5002)	2275	43.4% (987)	1843	34.3% (633)	2867	29.9% (856)	6068	38.4% (2329)	1040	18.9% (197)
SGA <3rd centile	14093	16.4% (2307)	2275	21.5% (490)	1843	15.1% (278)	2867	12.0% (344)	6068	18.5% (1120)	1040	7.2% (75)
Stillbirth	16588	2.8% (469)	2837	3.8% (109)	2245	3.7% (84)	3548	1.6% (55)	6863	2.5% (175)	1095	4.2% (46)

*By definition, participants with <2 maternal weight measurements could not have an estimated gestational weight gain.

APPENDIX D – USE OF OTHER MEASURES OF GESTATIONAL WEIGHT GAIN IN THE STUDY POPULATION

1: Institute of Medicine (IOM) gestational weight gain adequacy ratio

We used the IOM 2009 guidelines to estimate a GWG adequacy ratio for each study participant.(1). The adequacy ratio was calculated as: (observed/recommended weight gain)×100.(2) A participant with an IOM adequacy ratio of 100 has a total GWG equal to the average recommended weight gain for their gestational age. Recommended weight gain was calculated as: 1st trimester recommended weight gain + [2nd & 3rd trimester recommended weekly weight gain rate×(final gestational age weeks - 13)]. We assumed 1st trimester weight gains of 2kg for underweight/normal BMI participants and 0.5kg for overweight/obese. We assumed 2nd & 3rd trimester weight gain rates of 0.51, 0.42, 0.28, 0.22 kg/week for underweight, normal, overweight, and obese respectively. The IOM adequacy ratio was then grouped into BMI- and cohort-specific quartile groups to estimate risk and ARR of primary outcomes using the same approach outlined above.

1. IOM (Institute of Medicine) and NRC (National Research Council). Weight Gain During Pregnancy: Reexamining the Guidelines. Washington, DC: The National Academies Press; 2009.
2. Hutcheon JA, Bodnar LM, Joseph K, Abrams B, Simhan HN, Platt RW. The bias in current measures of gestational weight gain. Paediatric and perinatal epidemiology. 2012;26(2):109-16.

Table D0. Total gestational weight gain in the study population compared to IOM 2009 guidelines.

BMI	IOM Recommendations[a]		Overall		AMANHI-Bangladesh		AMANHI-Pakistan		PreSSMat		GARBH-Ini		ZAPPS	
	**Interpretation**	**Range, Kg**	**n**	**%**	**n**	**%**	**n**	**%**	**n**	**%**	**n**	**%**	**n**	**%**
Underweight	Inadequate	<12.5	3121	88.3	861	93.6	369	81.5	470	85.6	1391	88.8	30	63.8
	Adequate	12.5-18	404	11.4	57	6.2	81	17.9	78	14.2	171	10.9	17	36.2
	Excessive	>18	11	0.3	2	0.2	3	0.7	1	0.2	5	0.3		0.0
Normal	Inadequate	<11.5	8015	87.3	1568	92.5	915	87.6	1882	85.1	3211	87.3	439	80.1
	Adequate	11.5-16	992	10.8	110	6.5	109	10.4	296	13.4	396	10.8	81	14.8
	Excessive	>16	171	1.9	18	1.1	20	1.9	34	1.5	71	1.9	28	5.1
Overweight	Inadequate	<7	1294	63.2	103	69.6	274	71.4	365	61.0	400	61.6	152	56.5
	Adequate	7-11.5	590	28.8	38	25.7	85	22.1	187	31.3	197	30.4	83	30.9
	Excessive	>11.5	164	8.0	7	4.7	25	6.5	46	7.7	52	8.0	34	12.6
Obese	Inadequate	<5	238	45.4	10	45.5	75	52.1	46	47.4	50	45.5	57	37.7
	Adequate	5-9	176	33.6	6	27.3	47	32.6	38	39.2	30	27.3	55	36.4
	Excessive	>9	110	21.0	6	27.3	22	15.3	13	13.4	30	27.3	39	25.8

[a]IOM 2009 guidelines: https://doi.org/10.17226/12584. Recommendations are for total gestational weight gain at 40 weeks of gestation. Recommendations ranges and corresponding number of participants that fall into each grouping do not adjust for differences in length of gestation across the study population (i.e., shorter gestations result in lower total weight gain).

Figure D1. IOM weight gain adequacy ratio distribution in the study population (AMANHI-Bangladesh).

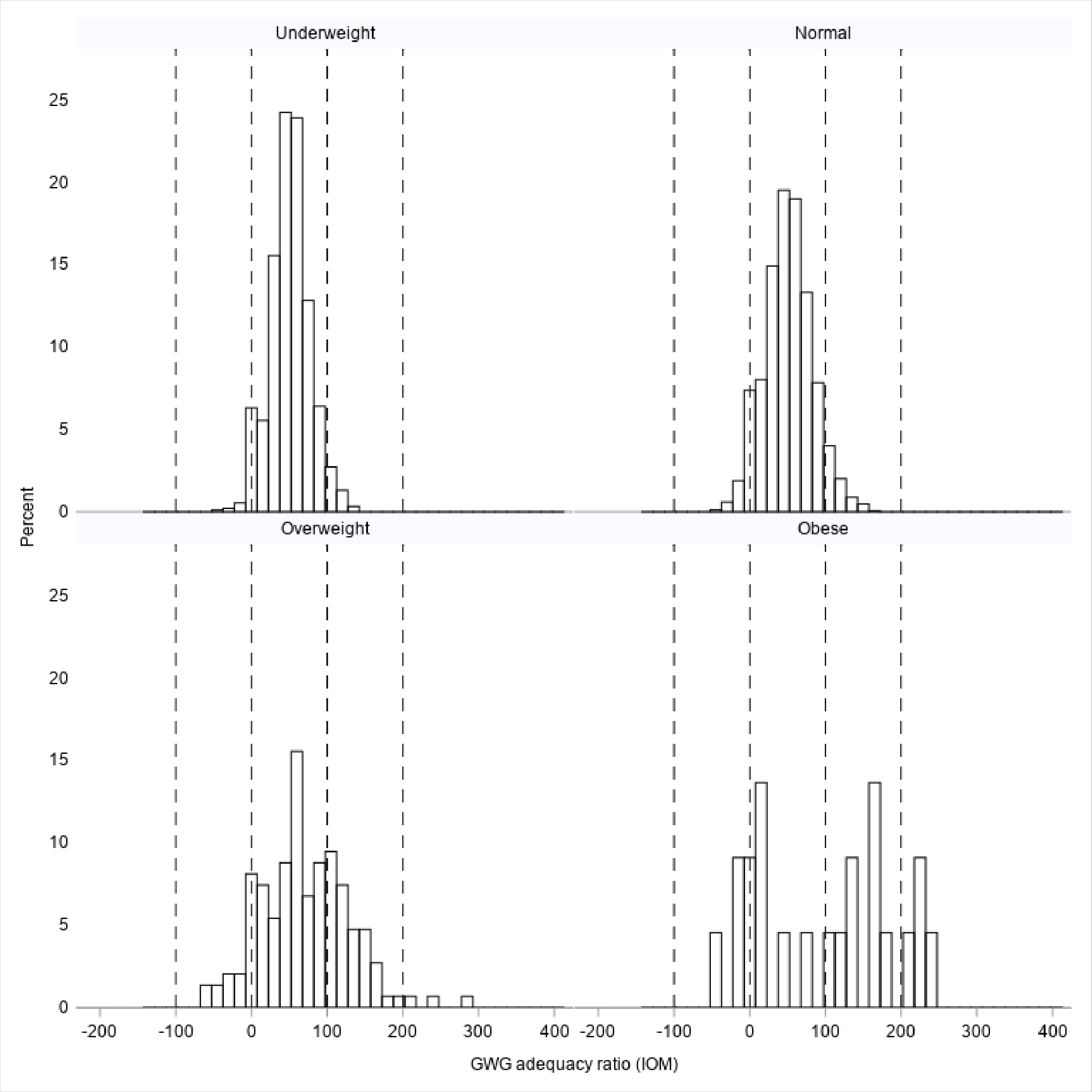

Figure D2. IOM weight gain adequacy ratio distribution in the study population (AMANHI-Pakistan).

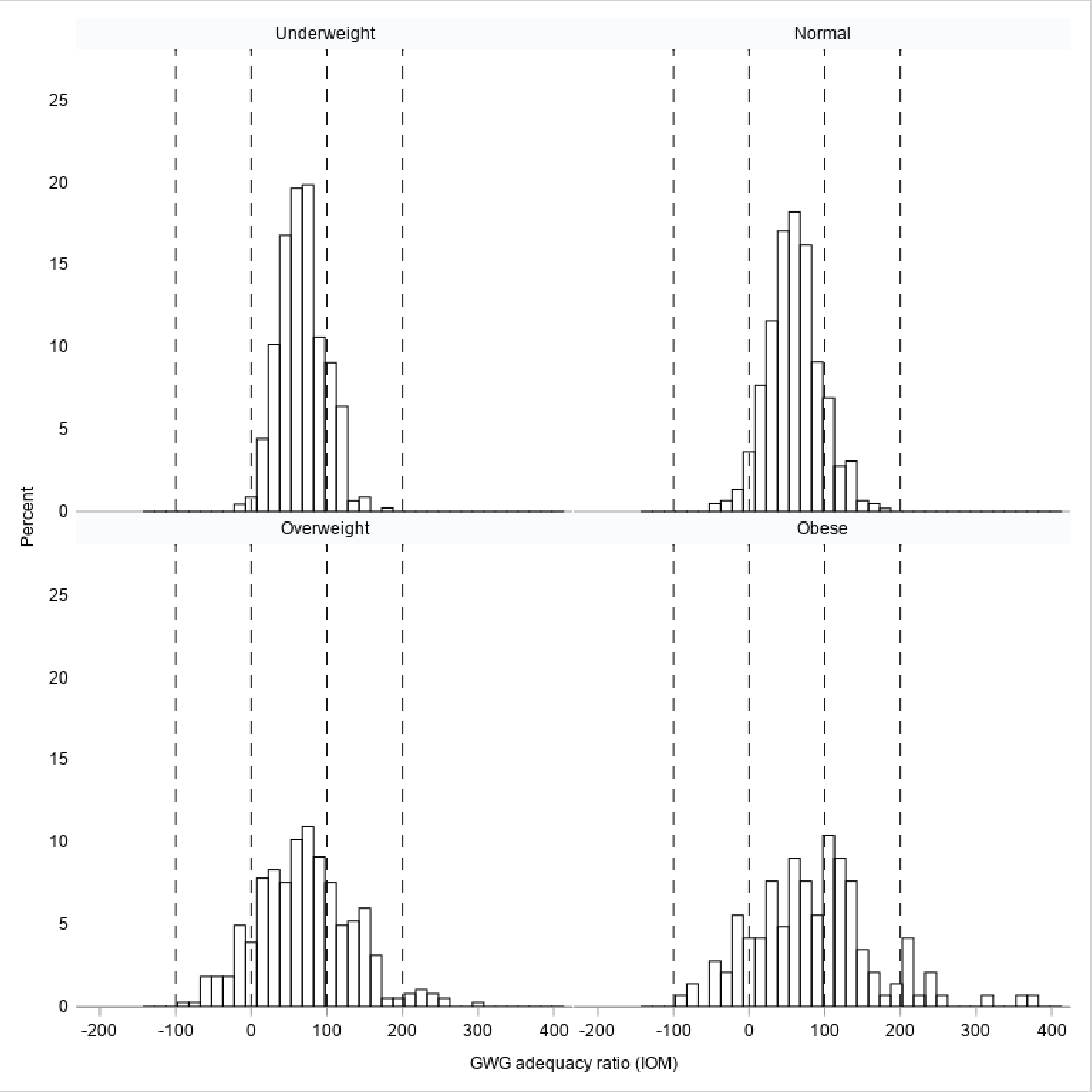

Figure D3. IOM weight gain adequacy ratio distribution in the study population (PreSSMat).

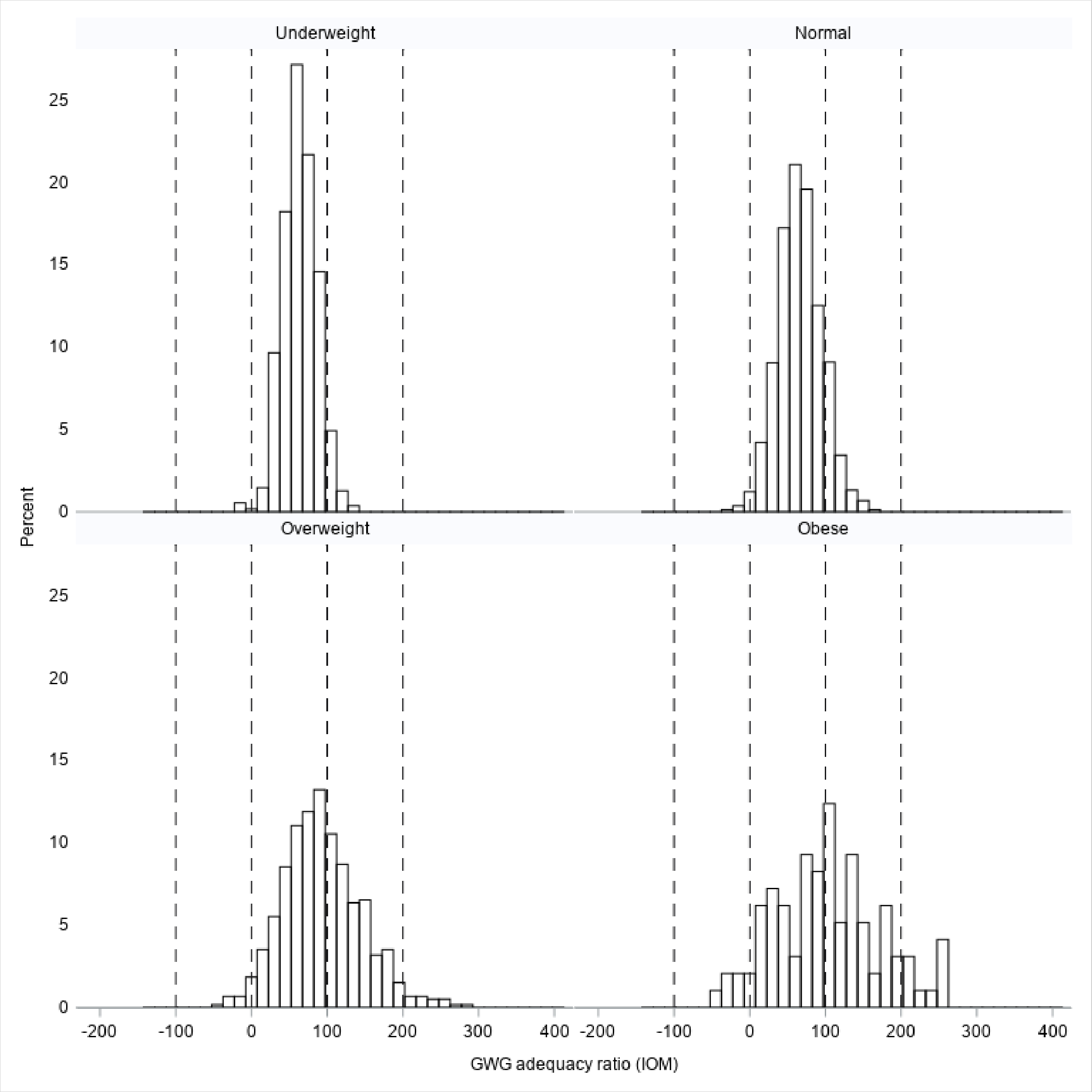

Figure D4. IOM weight gain adequacy ratio distribution in the study population (GARBH-Ini).

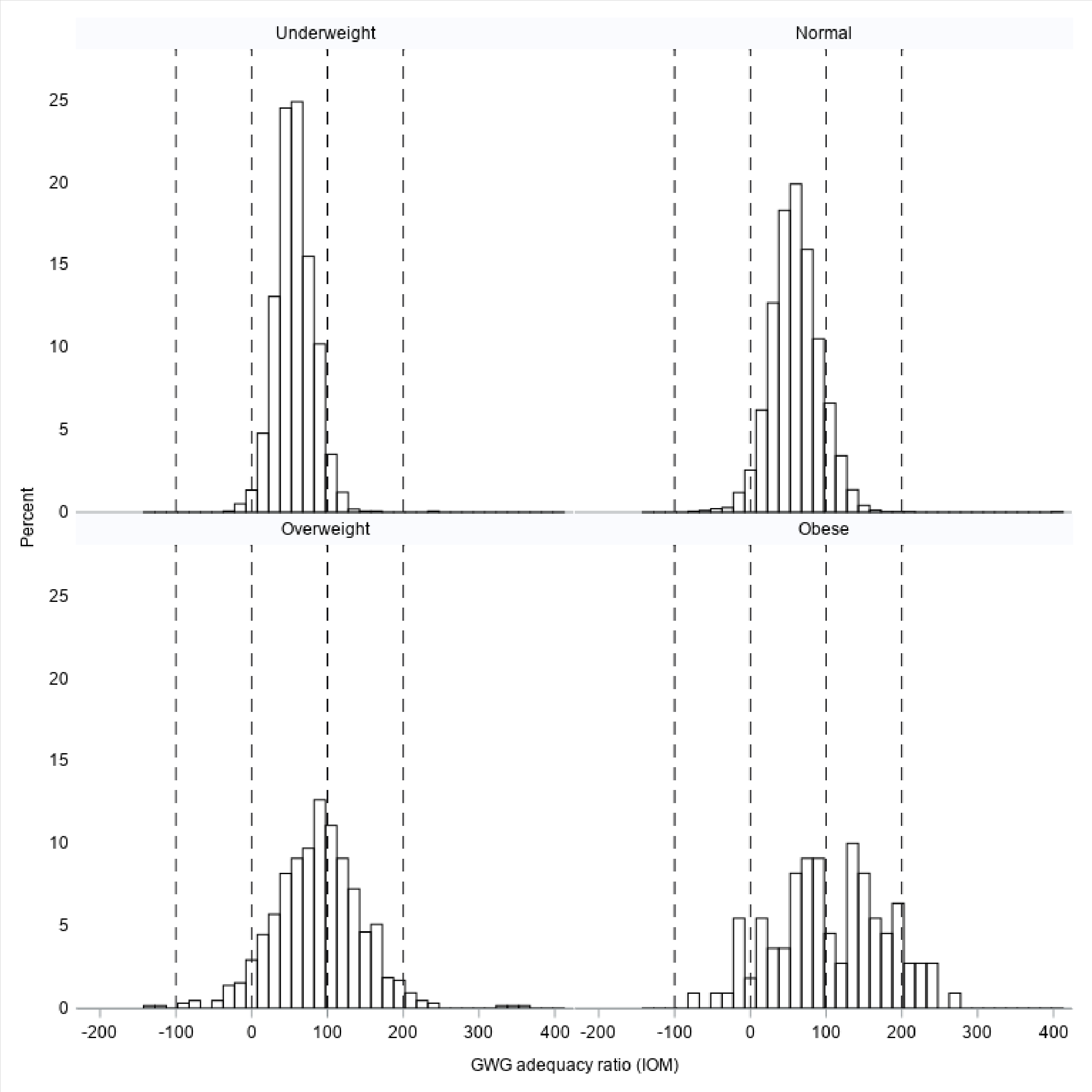

Figure D5. IOM weight gain adequacy ratio distribution in the study population (ZAPPS).

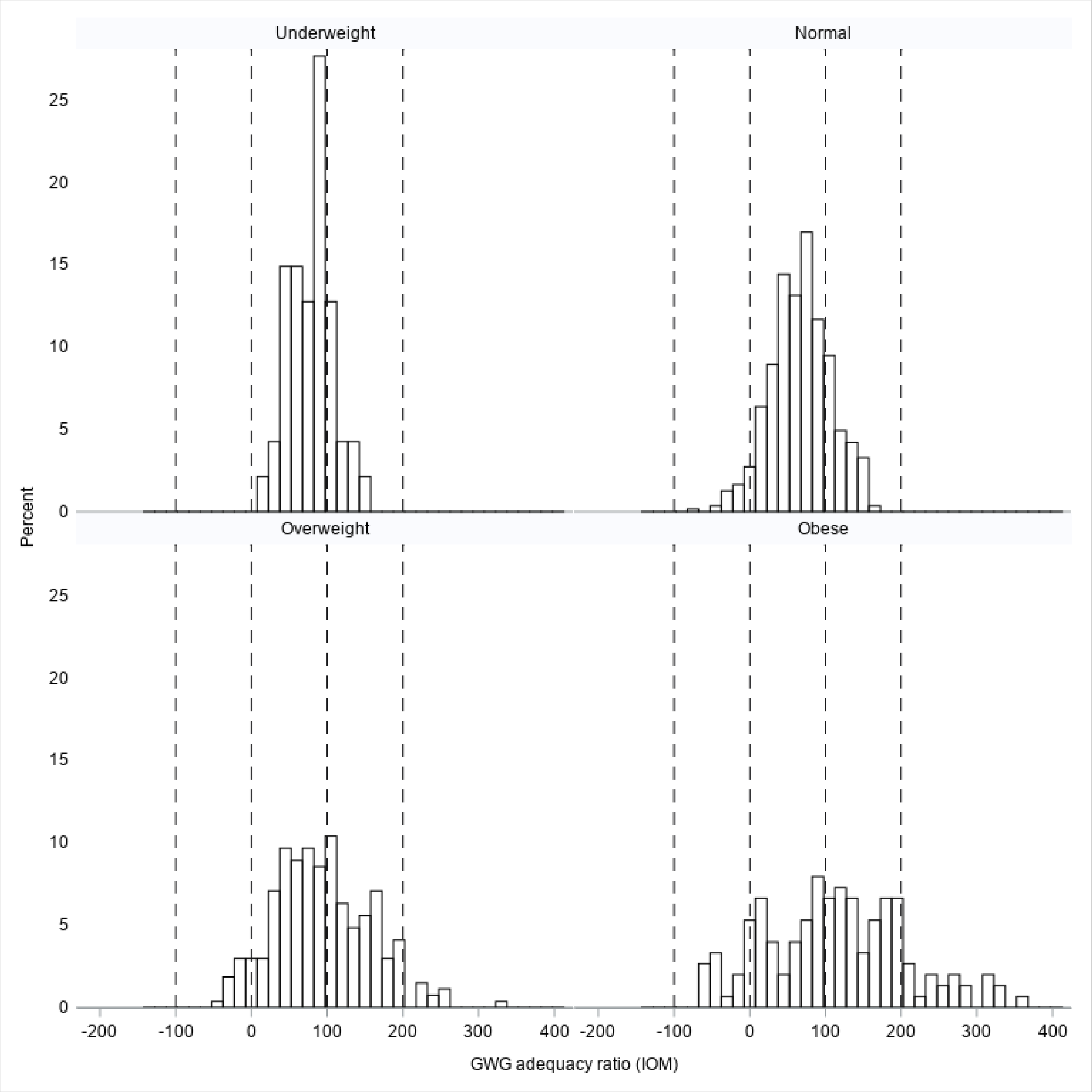

Table D1. Quartile group cut-offs of IOM weight gain adequacy ratio (AMANHI-Bangladesh).

BMI	Quartile group	N	Min	25th Pctl	50th Pctl	75th Pctl	Max
Underweight	1: lowest	230	-46.0	3.0	22.4	30.0	35.1
	2: second	230	35.1	39.9	43.3	47.2	50.6
	3: third	230	50.6	54.9	58.8	62.6	66.4
	4: highest	230	66.5	71.4	80.0	92.2	140.0
Normal	1: lowest	424	-43.2	0.0	13.3	23.2	29.8
	2: second	424	30.0	36.0	41.1	45.9	50.7
	3: third	424	50.7	55.2	60.0	64.8	70.7
	4: highest	424	70.8	77.9	86.9	99.8	168.4
Overweight	1: lowest	37	-64.0	-8.8	1.4	15.2	27.5
	2: second	37	28.5	46.5	53.9	63.0	66.0
	3: third	37	66.0	77.5	86.3	99.2	105.7
	4: highest	37	107.0	115.8	133.5	150.1	282.2
Obese	1: lowest	5	-42.2	-15.8	-8.7	0.0	0.0
	2: second	6	8.4	19.3	31.7	75.1	104.3
	3: third	6	126.1	138.2	150.1	169.5	171.8
	4: highest	5	178.2	214.9	227.1	228.9	242.9

Table D2. Quartile group cut-offs of IOM weight gain adequacy ratio (AMANHI-Pakistan).

BMI	Quartile group	N	Min	25th Pctl	50th Pctl	75th Pctl	Max
Underweight	1: lowest	113	-18.6	23.3	32.9	39.9	45.9
	2: second	113	46.5	50.5	55.2	60.6	65.5
	3: third	114	65.5	69.8	73.9	78.7	85.0
	4: highest	113	85.0	94.8	103.2	116.3	180.1
Normal	1: lowest	261	-50.0	7.8	20.2	29.8	36.7
	2: second	261	37.1	43.8	49.3	53.8	59.5
	3: third	261	59.7	64.0	68.7	73.5	80.1
	4: highest	261	80.2	89.1	100.4	116.8	187.1
Overweight	1: lowest	96	-82.9	-22.1	2.1	16.4	26.0
	2: second	96	26.0	39.9	49.7	59.7	68.5
	3: third	96	68.8	76.7	86.8	97.6	109.8
	4: highest	96	109.9	127.7	146.4	161.6	299.3
Obese	1: lowest	36	-92.4	-23.2	-7.3	17.8	27.7
	2: second	36	30.5	47.8	57.9	71.3	82.0
	3: third	36	85.4	102.5	107.3	113.9	123.8
	4: highest	36	129.6	141.8	170.3	212.7	377.6

Table D3. Quartile group cut-offs of IOM weight gain adequacy ratio (PreSSMat).

BMI	Quartile group	N	Min	25th Pctl	50th Pctl	75th Pctl	Max
Underweight	1: lowest	137	-16.7	32.1	38.7	43.6	49.0
	2: second	137	49.1	53.1	56.6	60.4	63.3
	3: third	138	63.3	66.9	70.7	74.4	78.9
	4: highest	137	78.9	83.9	88.8	98.4	131.7
Normal	1: lowest	553	-32.0	23.4	34.7	41.6	46.5
	2: second	553	46.6	51.7	55.9	60.3	64.8
	3: third	553	64.8	69.5	74.8	79.8	84.6
	4: highest	553	84.6	91.1	99.8	110.6	161.2
Overweight	1: lowest	149	-37.6	20.4	37.5	48.6	58.3
	2: second	150	58.4	66.3	74.4	82.2	89.1
	3: third	150	89.1	96.9	103.1	113.9	123.4
	4: highest	149	123.6	139.1	154.0	181.4	291.0
Obese	1: lowest	24	-41.9	-1.5	20.5	34.7	47.1
	2: second	24	50.5	67.7	79.0	85.7	99.5
	3: third	25	101.1	107.6	116.9	132.5	146.4
	4: highest	137	-16.7	32.1	38.7	43.6	49.0

Table D4. Quartile group cut-offs of IOM weight gain adequacy ratio (GARBH-Ini).

BMI	Quartile group	N	Min	25th Pctl	50th Pctl	75th Pctl	Max
Underweight	1: lowest	392	-29.6	21.1	31.2	36.5	41.2
	2: second	391	41.2	45.1	48.3	52.0	55.8
	3: third	392	55.9	59.5	63.3	67.1	72.7
	4: highest	392	72.7	79.4	85.3	95.4	239.3
Normal	1: lowest	919	-70.7	13.5	25.4	32.5	39.2
	2: second	920	39.2	44.7	49.7	53.9	58.7
	3: third	920	58.7	63.3	68.1	73.5	79.5
	4: highest	919	79.6	87.2	96.5	110.6	408.5
Overweight	1: lowest	162	-139.9	2.5	24.1	40.3	50.3
	2: second	162	50.9	58.7	72.6	81.0	89.3
	3: third	163	89.5	96.3	104.2	113.0	122.4
	4: highest	162	122.8	137.2	149.6	169.4	364.3
Obese	1: lowest	27	-73.3	-11.5	13.9	34.0	56.4
	2: second	28	56.9	67.9	77.3	86.9	101.2
	3: third	28	103.2	127.3	138.3	149.3	160.9
	4: highest	27	165.1	178.7	195.3	222.0	264.2

Table D5. BMI- and cohort-specific quartile groups of IOM weight gain adequacy ratio (ZAPPS).

BMI	Quartile group	N	Min	25th Pctl	50th Pctl	75th Pctl	Max
Underweight	1: lowest	12	8.3	39.0	49.8	51.7	54.6
	2: second	11	57.7	65.2	67.7	74.0	80.1
	3: third	12	83.0	86.1	87.7	94.0	94.2
	4: highest	12	94.7	103.0	110.6	127.6	154.0
Normal	1: lowest	135	-72.2	4.2	20.2	34.0	41.8
	2: second	140	42.0	49.5	55.7	61.0	67.6
	3: third	136	67.8	71.7	77.8	85.1	92.6
	4: highest	137	92.9	102.4	112.8	133.8	169.1
Overweight	1: lowest	67	-47.1	0.0	26.4	41.5	45.7
	2: second	67	47.8	58.3	67.8	76.4	84.5
	3: third	68	84.7	99.7	105.9	121.5	140.8
	4: highest	67	141.6	154.1	170.4	195.5	326.5
Obese	1: lowest	38	-64.4	-27.1	0.0	17.9	37.8
	2: second	37	42.1	71.5	86.5	96.7	108.5
	3: third	38	108.6	125.2	141.3	160.1	174.9
	4: highest	38	177.8	190.6	212.8	268.3	353.9

Table D6. Risk ratios for the association between IOM gestational weight adequacy ratio (grouped into quartiles) and adverse outcomes (preterm birth, PTB; low birthweight)

		Overall			AMANHI-Bangladesh			AMANHI-Pakistan			PreSSMat[b]			GARBH-Ini			ZAPPS		
PTB																			
BMI[a]	IOM[b] qtle	Risk (n/N)	Adj Risk,[c] % (95% CI)	Adj RR[c], (95% CI)	Risk (n/N)	Adj Risk,[c] % (95% CI)	Adj RR[c], (95% CI)	Risk (n/N)	Adj Risk,[c] % (95% CI)	Adj RR[c], (95% CI)	Risk (n/N)	Adj Risk,[c] % (95% CI)	Adj RR[c], (95% CI)	Risk (n/N)	Adj Risk,[c] % (95% CI)	Adj RR[c], (95% CI)	Risk (n/N)	Adj Risk,[c] % (95% CI)	Adj RR[c] (95% CI)
1	1	158/884	17.6 (14.3, 21.8)	1.52 (1.27, 1.81)	39/230	16.1 (11.9, 21.7)	1.47 (0.94, 2.29)	27/113	24.2 (17.4, 33.5)	1.84 (1.02, 3.34)	15/137	8.2 (4.9, 13.9)	0.77 (0.39, 1.54)	75/392	19.4 (15.8, 23.7)	1.68 (1.20, 2.34)	2/12		
	2	129/882	13.5 (11.0, 16.6)	1.16 (0.92, 1.46)	45/230	17.4 (13.3, 22.8)	1.59 (1.04, 2.44)	18/113	15.5 (10.1, 23.8)	1.18 (0.62, 2.23)	13/137	7.6 (4.4, 13.0)	0.71 (0.36, 1.41)	51/391	11.9 (9.2, 15.5)	1.03 (0.72, 1.49)	2/11		
	3	109/886	11.6 (11.1, 12.2)	1	28/230	11.0 (7.7, 15.6)	1	16/114	13.1 (8.1, 21.2)	1	16/138	10.7 (6.7, 17.0)	1.00 (1.00, 1.00)	49/392	11.6 (8.8, 15.1)	1	0/12		
	4	117/884	11.9 (10.0, 14.2)	1.03 (0.86, 1.23)	30/230	11.7 (8.4, 16.1)	1.06 (0.67, 1.70)	26/113	18.8 (13.1, 27.1)	1.43 (0.81, 2.54)	12/137	7.7 (4.4, 13.4)	0.72 (0.37, 1.42)	47/392	10.7 (8.0, 14.1)	0.92 (0.63, 1.35)	2/12		
2	1	352/2292	14.5 (13.6, 15.3)	1.51 (1.42, 1.61)	61/424	13.8 (10.9, 17.6)	1.64 (1.12, 2.41)	41/261	13.7 (10.2, 18.3)	1.79 (1.10, 2.92)	75/553	12.2 (9.7, 15.3)	1.36 (0.97, 1.90)	151/919	15.3 (13.1, 17.8)	1.47 (1.16, 1.86)	24/135	14.7 (9.8, 22.1)	1.50 (0.81, 2.79)
	2	279/2298	11.5 (10.5, 12.7)	1.21 (1.04, 1.40)	62/424	13.5 (10.7, 17.2)	1.61 (1.10, 2.35)	25/261	8.4 (5.7, 12.2)	1.10 (0.64, 1.90)	68/553	10.8 (8.6, 13.6)	1.20 (0.86, 1.69)	111/920	11.5 (9.7, 13.7)	1.10 (0.86, 1.42)	13/140	9.0 (5.4, 14.8)	0.92 (0.46, 1.84)
	3	229/2294	9.6 (8.8, 10.4)	1	38/424	8.4 (6.2, 11.4)	1	22/261	7.6 (5.1, 11.5)	1	51/553	9.0 (6.9, 11.6)	1.00 (1.00, 1.00)	101/920	10.4 (8.7, 12.5)	1	17/136	9.8 (5.9, 16.3)	1
	4	247/2294	10.3 (9.0, 11.9)	1.08 (0.89, 1.31)	42/424	9.7 (7.3, 12.9)	1.15 (0.76, 1.74)	44/261	14.7 (11.1, 19.5)	1.93 (1.20, 3.12)	55/553	9.5 (7.4, 12.3)	1.06 (0.74, 1.52)	94/919	9.8 (8.1, 11.9)	0.94 (0.72, 1.22)	12/137	6.8 (3.8, 12.1)	0.69 (0.34, 1.41)
3	1	77/511	13.7 (11.0, 17.1)	1.42 (0.95, 2.13)	5/37			19/96	18.3 (11.9, 28.2)	1.54 (0.79, 2.99)	22/149	11.9 (8.1, 17.6)	1.62 (0.85, 3.07)	25/162	13.3 (9.1, 19.6)	1.64 (0.89, 3.03)	6/67	6.1 (2.9, 12.8)	0.46 (0.20, 1.05)
	2	80/512	14.6 (13.0, 16.3)	1.51 (1.10, 2.08)	6/36			14/96	13.4 (8.0, 22.4)	1.12 (0.55, 2.28)	25/150	14.6 (10.1, 21.1)	1.98 (1.05, 3.74)	24/162	13.0 (9.0, 18.8)	1.60 (0.87, 2.94)	11/67	12.3 (6.7, 22.6)	0.93 (0.48, 1.80)
	3	53/514	9.6 (6.9, 13.3)	1	0/37			12/96	11.9 (7.2, 19.7)	1	13/150	7.4 (4.3, 12.7)	1.00 (1.00, 1.00)	14/163	8.1 (4.9, 13.3)	1	14/68	13.3 (7.9, 22.3)	1
	4	76/511	13.8 (10.9, 17.6)	1.44 (0.92, 2.25)	5/35			19/96	18.3 (12.0, 27.9)	1.54 (0.79, 2.98)	20/149	13.5 (8.9, 20.5)	1.83 (0.93, 3.59)	26/162	14.6 (10.1, 21.2)	1.80 (0.98, 3.30)	6/67	5.6 (2.5, 12.5)	0.42 (0.17, 1.04)
4	1	22/130	16.4 (10.1, 26.8)	1.06 (0.47, 2.41)	0/5			10/36	23.3 (13.6, 40.1)	3.71 (1.06,12.97)	4/24	12.8 (4.6, 35.8)	0.82 (0.26, 2.61)	6/27	16.9 (7.8, 36.7)	0.78 (0.32, 1.95)	2/38		
	2	19/131	13.2 (10.8, 16.1)	0.85 (0.50, 1.47)	0/6			6/36	11.9 (5.6, 24.9)	1.88 (0.52, 6.84)	4/24	14.5 (6.6, 31.9)	0.92 (0.32, 2.68)	5/28	16.3 (7.2, 36.7)	0.76 (0.26, 2.19)	4/37		
	3	24/133	15.4 (8.3, 28.7)	1	1/6			3/36	6.3 (1.9, 20.7)	1	8/25	15.7 (6.8, 36.5)	1.00 (1.00, 1.00)	9/28	21.5 (11.5, 40.1)	1	3/38		
	4	22/130	15.1 (11.5, 19.9)	0.98 (0.49, 1.96)	2/5			7/36	14.6 (6.6, 32.4)	2.33 (0.57, 9.46)	6/24	13.3 (5.3, 33.4)	0.84 (0.36, 2.01)	2/27	7.5 (1.9, 30.2)	0.35 (0.09, 1.39)	5/38		
LBW <2500G																			
BMI[a]	IOM[b] qtle	Risk (n/N)	Adj Risk,[c] % (95% CI)	Adj RR[c], (95% CI)	Risk (n/N)	Adj Risk,[c] % (95% CI)	Adj RR[c], (95% CI)	Risk (n/N)	Adj Risk,[c] % (95% CI)	Adj RR[c], (95% CI)	Risk (n/N)	Adj Risk,[c] % (95% CI)	Adj RR[c], (95% CI)	Risk (n/N)	Adj Risk,[c] % (95% CI)	Adj RR[c], (95% CI)	Risk (n/N)	Adj Risk,[c] % (95% CI)	Adj RR[c] (95% CI)
1	1	389/884	43.2 (38.1, 49.0)	1.55 (1.43, 1.69)	119/230	50.3 (43.8, 57.8)	1.65 (1.31, 2.10)	51/113	46.6 (37.6, 57.7)	1.76 (1.21, 2.55)	46/137	29.8 (22.6, 39.4)	1.50 (0.95, 2.36)	170/392	43.0 (38.0, 48.6)	1.48 (1.21, 1.80)	3/12		
	2	321/882	34.8 (29.2, 41.5)	1.25 (1.07, 1.46)	111/230	45.4 (39.2, 52.6)	1.49 (1.17, 1.90)	38/113	32.6 (24.8, 42.9)	1.23 (0.82, 1.84)	36/137	24.3 (17.9, 33.0)	1.22 (0.77, 1.94)	132/391	31.9 (27.7, 36.9)	1.10 (0.89, 1.35)	4/11		
	3	252/886	27.8 (24.7, 31.4)	1	73/230	30.4 (25.0, 37.1)	1	32/114	26.5 (19.5, 36.0)	1	27/138	19.9 (13.8, 28.7)	1	118/392	29.1 (24.9, 34.0)	1	1/12		
	4	203/884	21.8 (19.0, 25.0)	0.78 (0.68, 0.90)	61/230	25.1 (20.0, 31.4)	0.83 (0.61, 1.11)	35/113	26.0 (19.1, 35.4)	0.98 (0.65, 1.49)	23/137	16.5 (11.1, 24.6)	0.83 (0.49, 1.40)	83/392	20.2 (16.6, 24.6)	0.69 (0.54, 0.89)	1/12		
2	1	746/2292	31.3 (28.9, 33.9)	1.59 (1.45, 1.74)	161/424	36.7 (31.9, 42.2)	1.78 (1.40, 2.25)	88/261	32.1 (26.6, 38.8)	1.64 (1.20, 2.23)	153/553	26.5 (22.8, 30.7)	1.59 (1.24, 2.02)	312/919	32.9 (30.0, 36.2)	1.47 (1.26, 1.71)	30/135	17.7 (12.4, 25.3)	1.78 (0.99, 3.18)
	2	589/2298	24.1 (21.8, 26.8)	1.23 (1.07, 1.41)	144/424	31.8 (27.5, 36.9)	1.54 (1.21, 1.96)	63/261	22.5 (17.9, 28.2)	1.14 (0.82, 1.60)	124/553	20.0 (16.9, 23.7)	1.20 (0.93, 1.54)	239/920	24.9 (22.2, 27.8)	1.11 (0.94, 1.30)	19/140	12.5 (8.2, 19.3)	1.26 (0.69, 2.29)
	3	476/2294	19.7 (17.4, 22.2)	1	94/424	20.7 (17.0, 25.1)	1	53/261	19.6 (15.2, 25.3)	1	97/553	16.7 (13.7, 20.3)	1	214/920	22.4 (19.9, 25.3)	1	18/136	9.9 (6.1, 16.2)	1
	4	360/2294	15.0 (11.9, 18.8)	0.76 (0.62, 0.94)	96/424	21.6 (17.9, 26.0)	1.04 (0.80, 1.36)	53/261	18.5 (14.2, 24.1)	0.94 (0.65, 1.36)	52/553	8.9 (6.6, 12.0)	0.53 (0.38, 0.76)	146/919	15.6 (13.4, 18.2)	0.70 (0.57, 0.84)	13/137	7.1 (4.2, 12.1)	0.71 (0.36, 1.41)
3	1	106/511	18.9 (15.2, 23.3)	1.46 (1.10, 1.95)	7/37			26/96	23.7 (16.4, 34.3)	1.73 (0.94, 3.20)	31/149	16.4 (11.3, 23.7)	1.98 (1.04, 3.77)	37/162	20.5 (15.1, 27.8)	1.49 (0.94, 2.35)	6/67	7.1 (3.1, 16.4)	0.55 (0.21, 1.45)
	2	104/512	18.2 (15.9, 20.9)	1.41 (1.03, 1.94)	6/37			21/96	19.0 (11.9, 30.2)	1.38 (0.72, 2.68)	34/150	20.6 (14.9, 28.5)	2.49 (1.30, 4.80)	34/162	17.9 (13.1, 24.6)	1.30 (0.82, 2.06)	9/67	11.5 (6.3, 20.9)	0.89 (0.40, 1.99)
	3	72/514	12.9 (10.2, 16.2)	1	7/37			15/96	13.7 (8.2, 23.0)	1	14/150	8.3 (4.6, 14.8)	1	26/163	13.8 (9.6, 19.8)	1	11/68	12.9 (7.1, 23.5)	1
	4	60/511	11.2 (9.5, 13.4)	0.87 (0.71, 1.08)	5/37			13/96	12.4 (7.4, 20.9)	0.91 (0.43, 1.90)	14/149	9.3 (5.3, 16.3)	1.13 (0.51, 2.48)	21/162	12.4 (8.2, 18.7)	0.90 (0.52, 1.53)	6/67	7.3 (3.2, 16.7)	0.57 (0.21, 1.50)
4	1	27/130	18.8 (11.0, 32.1)	2.23 (0.88, 5.65)	1/5			13/36	29.6 (17.3, 50.8)	8.68 (1.64,45.85)	5/24	1.5 (0.0, 1423)	1.87 (0.47, 7.43)	6/27	0.4 (0.0, 373.8)	1.10 (0.31, 3.95)	3/38	6.3 (1.5, 25.9)	1.19 (0.23, 5.98)
	2	21/131	13.8 (9.2, 20.8)	1.64 (0.71, 3.76)	1/6			9/36	21.1 (11.2, 39.9)	6.18 (1.11,34.54)	3/24	1.6 (0.0, 1341)	2.07 (0.40,10.70)	4/28	0.3 (0.0, 220.6)	0.85 (0.18, 4.17)	4/37	8.3 (2.9, 24.0)	1.57 (0.40, 6.14)
	3	14/133	8.5 (4.9, 14.5)	1	0/6			2/36	3.4 (0.7, 17.5)	1	3/25	0.8 (0.0, 806.9)	1	6/28	0.4 (0.0, 374.0)	1	3/38	5.3 (1.5, 19.0)	1
	4	16/130	9.7 (7.0, 13.4)	1.14 (0.63, 2.06)	1/5			4/36	7.9 (1.7, 36.6)	2.33 (0.33,16.49)	4/24	0.8 (0.0, 868.2)	1.00 (0.17, 5.83)	4/27	0.3 (0.0, 364.2)	0.91 (0.21, 3.95)	3/38	5.2 (1.8, 15.3)	0.98 (0.21, 4.54)

[a] BMI groups: 1, underweight; 2, normal; 3, overweight; 4, obese
[b] IOM adequacy ratio grouped into BMI- and site-specific quartiles
[c] Adjusted for maternal age, gestational age at enrollment, maternal height, maternal BMI, parous, and previous preterm birth

Table D7. Risk ratios for the association between IOM gestational weight adequacy ratio (grouped into quartiles) and small-for-gestational age ($<10^{th}$ percentile, SGA10; $<3^{rd}$ percentile, SGA3)

		Overall			AMANHI-Bangladesh			AMANHI-Pakistan			PreSSMat[b]			GARBH-Ini			ZAPPS		
SGA10																			
BMI[a]	IOM[b] qtle	Risk (n/N)	Adj Risk,[c] % (95% CI)	Adj RR[c], (95% CI)	Risk (n/N)	Adj Risk,[c] % (95% CI)	Adj RR[c], (95% CI)	Risk (n/N)	Adj Risk,[c] % (95% CI)	Adj RR[c], (95% CI)	Risk (n/N)	Adj Risk,[c] % (95% CI)	Adj RR[c], (95% CI)	Risk (n/N)	Adj Risk,[c] % (95% CI)	Adj RR[c], (95% CI)	Risk (n/N)	Adj Risk,[c] % (95% CI)	Adj RR[c] (95% CI)
1	1	497/883	54.1 (50.7, 57.7)	1.24 (1.15, 1.34)	145/230	61.2 (54.6, 68.7)	1.31 (1.09, 1.56)	55/113	48.1 (39.3, 58.9)	1.20 (0.90, 1.61)	74/137	50.5 (41.8, 61.1)	1.42 (1.05, 1.91)	220/391	53.7 (48.7, 59.2)	1.18 (1.02, 1.36)	3/12		
	2	439/881	48.7 (43.2, 54.8)	1.12 (1.02, 1.22)	137/230	58.2 (51.9, 65.3)	1.24 (1.04, 1.48)	56/113	47.5 (38.7, 58.3)	1.19 (0.89, 1.59)	51/137	36.0 (28.4, 45.5)	1.01 (0.72, 1.41)	191/390	47.9 (43.1, 53.3)	1.05 (0.90, 1.22)	4/11		
	3	389/885	43.6 (40.3, 47.3)	1	109/230	46.9 (40.7, 54.0)	1	48/114	40.0 (31.9, 50.1)	1	50/138	35.6 (28.1, 45.2)	1	181/391	45.6 (40.9, 50.9)	1	2/12		
	4	316/884	35.6 (31.9, 39.6)	0.82 (0.77, 0.87)	89/230	38.1 (32.0, 45.3)	0.81 (0.65, 1.01)	44/113	35.3 (27.3, 45.7)	0.88 (0.63, 1.24)	37/137	26.7 (19.7, 36.2)	0.75 (0.51, 1.10)	146/392	37.8 (33.1, 43.2)	0.83 (0.70, 0.98)	0/12		
2	1	1017/2285	43.7 (41.2, 46.4)	1.41 (1.30, 1.53)	217/424	49.8 (44.9, 55.2)	1.38 (1.17, 1.62)	118/261	44.6 (38.8, 51.4)	1.40 (1.12, 1.75)	220/553	39.2 (34.9, 43.9)	1.55 (1.29, 1.87)	426/913	45.9 (42.6, 49.5)	1.31 (1.17, 1.47)	35/134	25.6 (18.6, 35.2)	1.81 (1.08, 3.02)
	2	911/2294	38.0 (35.4, 40.8)	1.22 (1.14, 1.31)	189/424	43.0 (38.3, 48.2)	1.19 (1.00, 1.42)	95/261	34.9 (29.4, 41.3)	1.09 (0.86, 1.39)	195/552	33.2 (29.3, 37.6)	1.32 (1.08, 1.60)	391/917	41.2 (38.1, 44.5)	1.17 (1.05, 1.32)	41/140	28.3 (21.6, 37.0)	1.99 (1.23, 3.22)
	3	746/2291	31.1 (27.4, 35.2)	1	160/424	36.1 (31.7, 41.2)	1	87/261	32.0 (26.8, 38.1)	1	146/553	25.2 (21.6, 29.4)	1	333/918	35.1 (32.2, 38.3)	1	20/135	14.2 (9.5, 21.2)	1
	4	606/2293	25.4 (22.2, 29.0)	0.82 (0.76, 0.87)	142/424	32.0 (27.7, 36.9)	0.89 (0.74, 1.07)	75/261	26.7 (21.7, 33.0)	0.84 (0.64, 1.09)	111/553	19.5 (16.2, 23.4)	0.77 (0.61, 0.97)	261/918	27.5 (24.8, 30.6)	0.78 (0.69, 0.90)	18/137	12.6 (8.1, 19.6)	0.89 (0.49, 1.61)
3	1	144/511	26.7 (24.0, 29.6)	1.36 (1.14, 1.64)	9/37	21.7 (10.1, 46.3)	0.92 (0.39, 2.13)	29/96	28.2 (20.3, 39.1)	1.19 (0.74, 1.89)	44/149	26.8 (20.1, 35.7)	1.68 (1.06, 2.68)	48/162	28.8 (22.4, 37.0)	1.40 (0.97, 2.01)	13/67	16.9 (9.6, 29.8)	1.96 (0.84, 4.58)
	2	135/511	24.5 (22.3, 26.9)	1.25 (1.02, 1.54)	7/37	14.7 (7.0, 31.0)	0.62 (0.26, 1.46)	26/96	24.6 (16.8, 35.9)	1.04 (0.62, 1.72)	38/150	25.4 (19.0, 33.9)	1.60 (0.99, 2.58)	50/161	27.3 (21.4, 34.9)	1.33 (0.94, 1.88)	13/67	15.1 (9.3, 24.6)	1.75 (0.77, 3.97)
	3	110/514	19.6 (16.6, 23.1)	1	11/37	23.7 (13.7, 41.0)	1	25/96	23.7 (16.5, 34.1)	1	26/150	15.9 (10.8, 23.4)	1	41/163	20.6 (15.6, 27.2)	1	8/68	8.6 (4.3, 17.2)	1
	4	79/511	14.7 (12.0, 17.9)	0.75 (0.65, 0.87)	9/37	23.8 (11.9, 47.7)	1.01 (0.44, 2.31)	17/96	16.8 (10.7, 26.3)	0.71 (0.40, 1.25)	17/149	11.3 (6.8, 18.7)	0.71 (0.38, 1.33)	28/162	15.5 (11.0, 21.9)	0.75 (0.49, 1.16)	7/67	9.2 (4.4, 19.2)	1.06 (0.41, 2.75)
4	1	27/128	20.5 (14.9, 28.1)	1.65 (1.22, 2.23)	1/5			10/36	21.4 (10.0, 45.8)	1.50 (0.56, 4.02)	6/24			7/26	17.2 (6.2, 48.4)	1.87 (0.54, 6.43)	3/37	6.7 (1.7, 26.7)	1.45 (0.19, 11.30)
	2	21/131	14.7 (9.5, 22.6)	1.18 (0.83, 1.68)	1/6			7/36	19.1 (9.1, 40.3)	1.34 (0.45, 4.00)	1/24			6/28	12.5 (5.2, 30.3)	1.35 (0.26, 7.10)	6/37	8.8 (3.9, 20.3)	1.90 (0.44, 8.22)
	3	19/133	12.4 (8.7, 17.7)	1	1/6			7/36	14.3 (6.3, 32.4)	1	3/25			5/28	9.2 (2.0, 42.7)	1	3/38	4.7 (1.1, 20.3)	1
	4	13/130	8.3 (5.8, 11.8)	0.67 (0.41, 1.08)	0/5			3/36	5.5 (1.7, 17.9)	0.38 (0.10, 1.49)	3/24			4/27	7.5 (3.2, 17.6)	0.82 (0.15, 4.48)	3/38	6.2 (2.1, 18.7)	1.34 (0.27, 6.57)
SGA3																			
BMI[a]	IOM[b] qtle	Risk (n/N)	Adj Risk,[c] % (95% CI)	Adj RR[c], (95% CI)	Risk (n/N)	Adj Risk,[c] % (95% CI)	Adj RR[c], (95% CI)	Risk (n/N)	Adj Risk,[c] % (95% CI)	Adj RR[c], (95% CI)	Risk (n/N)	Adj Risk,[c] % (95% CI)	Adj RR[c], (95% CI)	Risk (n/N)	Adj Risk,[c] % (95% CI)	Adj RR[c], (95% CI)	Risk (n/N)	Adj Risk,[c] % (95% CI)	Adj RR[c] (95% CI)
1	1	273/883	29.0 (26.0, 32.4)	1.47 (1.15, 1.89)	83/230	34.3 (28.4, 41.4)	1.74 (1.25, 2.40)	29/113	22.5 (15.5, 32.8)	1.38 (0.82, 2.34)	42/137	25.9 (18.7, 35.7)	2.45 (1.36, 4.42)				2/12		
	2	237/881	25.8 (21.7, 30.6)	1.31 (1.04, 1.64)	78/230	31.7 (26.0, 38.6)	1.60 (1.15, 2.23)	29/113	22.7 (15.8, 32.7)	1.39 (0.83, 2.33)	25/137	15.9 (10.8, 23.5)	1.51 (0.81, 2.82)				4/11		
	3	179/885	19.7 (15.7, 24.6)	1	48/230	19.8 (15.0, 26.0)	1	21/114	16.3 (10.9, 24.4)	1	15/138	10.5 (6.3, 17.6)	1				1/12		
	4	133/884	14.7 (12.4, 17.4)	0.75 (0.65, 0.86)	40/230	16.6 (12.3, 22.4)	0.84 (0.56, 1.26)	16/113	12.5 (7.4, 21.0)	0.77 (0.40, 1.48)	14/137	9.8 (5.7, 16.9)	0.93 (0.46, 1.90)				0/12		
2	1	508/2285	21.1 (18.8, 23.7)	1.54 (1.44, 1.65)	120/424	26.7 (22.3, 32.0)	1.46 (1.12, 1.91)	56/261	20.0 (15.4, 26.0)	1.55 (1.04, 2.29)	98/553	17.2 (14.1, 21.0)	1.64 (1.19, 2.27)				11/134	5.4 (2.7, 10.6)	1.01 (0.42, 2.42)
	2	421/2294	16.8 (14.4, 19.6)	1.23 (1.13, 1.33)	104/424	23.1 (19.2, 27.8)	1.26 (0.97, 1.65)	51/261	17.7 (13.7, 23.1)	1.38 (0.93, 2.04)	74/552	11.9 (9.4, 15.2)	1.13 (0.80, 1.60)				17/140	7.4 (4.2, 13.1)	1.39 (0.64, 3.00)
	3	344/2291	13.7 (11.9, 15.8)	1	84/424	18.3 (14.8, 22.6)	1	37/261	12.9 (9.4, 17.6)	1	64/553	10.5 (8.1, 13.6)	1				10/135	5.4 (2.8, 10.3)	1
	4	243/2293	9.7 (7.5, 12.5)	0.71 (0.62, 0.81)	59/424	13.0 (10.0, 16.9)	0.71 (0.52, 0.98)	30/261	10.0 (6.9, 14.5)	0.77 (0.48, 1.24)	33/553	5.3 (3.7, 7.7)	0.51 (0.33, 0.79)				6/137	3.2 (1.5, 7.1)	0.60 (0.22, 1.65)
3	1	56/511	8.9 (6.6, 11.9)	1.34 (0.83, 2.18)	3/37			8/96	6.2 (2.9, 13.6)	0.96 (0.36, 2.61)	21/149	10.2 (6.1, 17.0)	3.10 (1.17, 8.21)				5/67	4.1 (1.1, 14.8)	1.42 (0.30, 6.65)
	2	58/511	8.6 (6.8, 11.0)	1.30 (0.90, 1.88)	4/37			9/96	6.1 (2.8, 13.3)	0.94 (0.35, 2.52)	13/150	8.1 (4.6, 14.1)	2.45 (0.90, 6.72)				4/67	3.7 (1.4, 10.1)	1.29 (0.27, 6.09)
	3	45/514	6.6 (5.1, 8.6)	1	7/37			10/96	6.5 (3.0, 14.0)	1	6/150	3.3 (1.4, 8.0)	1				3/68	2.9 (0.8, 10.9)	1
	4	28/511	4.8 (3.6, 6.3)	0.72 (0.56, 0.94)	3/37			6/96	4.2 (1.9, 9.1)	0.65 (0.24, 1.79)	4/149	2.5 (0.9, 7.3)	0.77 (0.20, 2.95)				2/67	2.5 (0.7, 9.4)	0.88 (0.13, 5.82)
4	1	14/128	9.7 (7.0, 13.5)	1.43 (0.63, 3.24)	0/5			6/36	4.8 (1.4, 16.1)	1.61 (0.28, 9.28)	4/24						2/37	4.8 (0.9, 27.3)	1.25 (0.09, 16.83)
	2	13/131	8.1 (5.2, 12.8)	1.20 (0.65, 2.21)	1/6			4/36	8.2 (3.1, 21.8)	2.74 (0.48, 15.74)	0/24						4/37	5.6 (2.0, 15.9)	1.45 (0.23, 9.28)
	3	11/133	6.8 (3.8, 12.0)	1	0/6			2/36	3.0 (0.5, 16.7)	1	1/25						2/38	3.9 (0.7, 21.7)	1
	4	8/130	5.2 (3.8, 6.9)	0.76 (0.40, 1.46)	0/5			2/36	2.5 (0.5, 13.0)	0.82 (0.12, 5.78)	2/24						2/38	3.7 (0.8, 17.8)	0.97 (0.12, 8.06)

[a] BMI groups: 1, underweight; 2, normal; 3, overweight; 4, obese

[b] IOM adequacy ratio grouped into BMI- and site-specific quartiles

[c] Adjusted for maternal age, gestational age at enrollment, maternal height, maternal BMI, parous, and previous preterm birth

2. Weight-gain-for-gestational-age Z-scores using INTERGROWTH-21st standards

Similar to the calculation of GWG Z-scores using the reference values from our study sub-population, we calculated weight gain Z-scores using the INTERGROWTH-21st standards.(1) This analysis was limited to normal BMI participants delivering at 14-40 weeks because this is the INTERGROWTH-21st target population.(9) To estimate the associated risk and ARR of primary outcomes, the INTERGROWTH-21st Z-scores were then categorized into 4 groups that correspond to the cut-offs for the ≤25th, 26-50th, 51-75th, and >75th percentiles of the standard normal distribution, respectively.

1. Ismail LC, Bishop DC, Pang R, Ohuma EO, Kac G, Abrams B, et al. Gestational weight gain standards based on women enrolled in the Fetal Growth Longitudinal Study of the INTERGROWTH-21st Project: a prospective longitudinal cohort study. bmj. 2016;352.

Figure D6. Study observed maternal weight gain (circles) and predicted percentile curves (blue), with overlayed INTERGROWTH-21st percentiles curves (red) in the reference sub-population. Normal BMI participants.

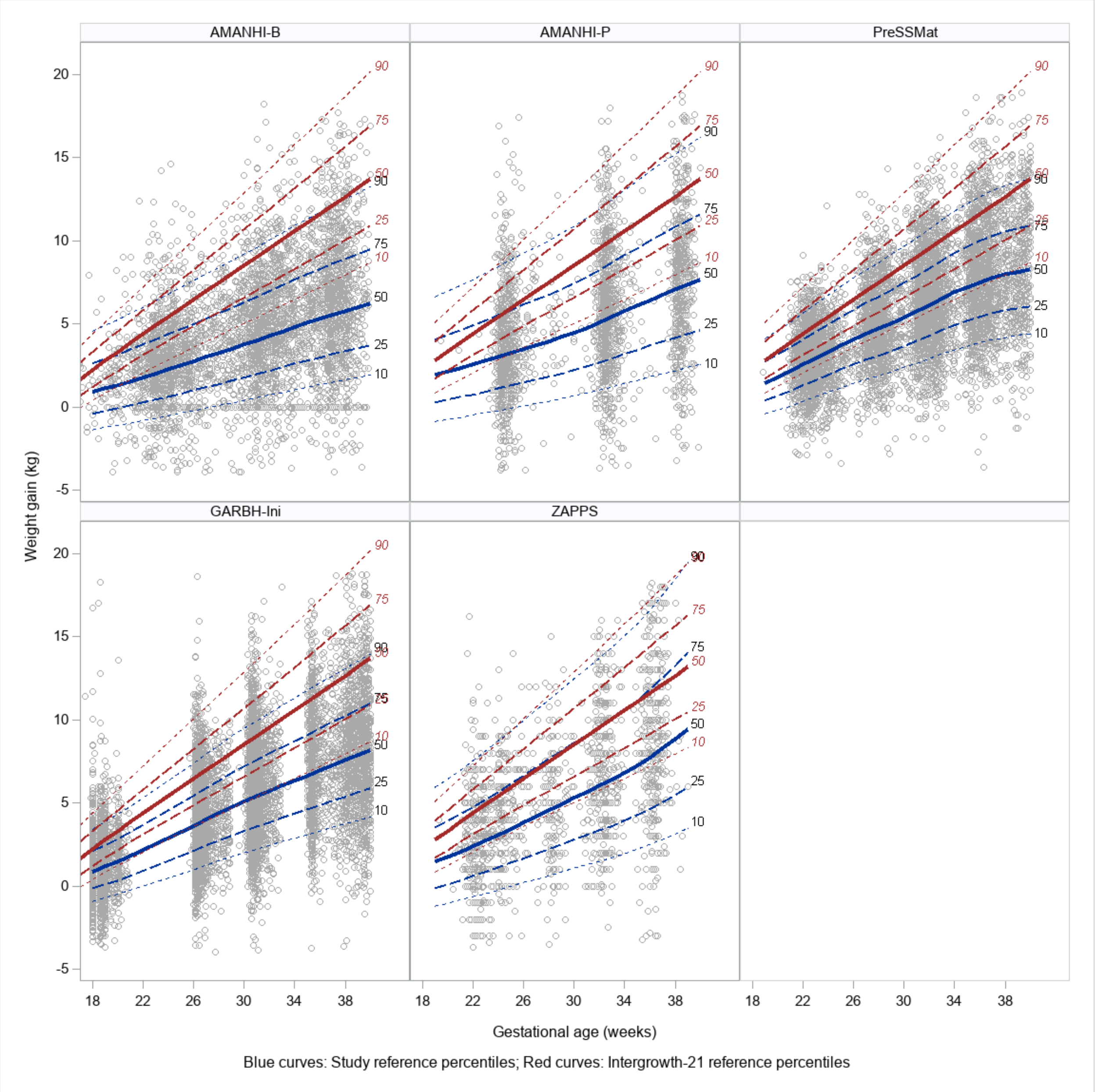

Table D8 – Study-specific percentiles of gestational weight gain for AMANHI-Bangladesh vs INTERGROWTH-21st standards, Normal BMI (Kg)

	Percentiles based on study reference sub-population (Kg)						Percentiles based on INTERGROWTH-21st standards (Kg)				
Gestational age, weeks	Observations, n	p10	p25	p50	p75	p90	p10	p25	p50	p75	p90
18	14	-1.37	-0.44	0.89	2.60	4.56	0.37	1.19	2.19	3.28	4.37
19	28	-1.25	-0.28	1.10	2.87	4.90	0.82	1.69	2.75	3.92	5.08
20	48	-1.11	-0.11	1.31	3.15	5.25	1.24	2.17	3.30	4.55	5.78
21	65	-0.97	0.07	1.54	3.44	5.61	1.65	2.64	3.84	5.17	6.49
22	129	-0.83	0.24	1.77	3.74	5.99	2.05	3.10	4.37	5.79	7.20
23	304	-0.69	0.43	2.00	4.04	6.37	2.44	3.54	4.90	6.41	7.91
24	286	-0.54	0.61	2.24	4.35	6.76	2.82	3.99	5.42	7.02	8.61
25	188	-0.39	0.80	2.49	4.66	7.15	3.19	4.42	5.94	7.63	9.32
26	140	-0.24	0.99	2.73	4.98	7.55	3.56	4.85	6.45	8.24	10.02
27	88	-0.09	1.18	2.98	5.30	7.95	3.92	5.28	6.96	8.84	10.73
28	97	0.07	1.38	3.23	5.62	8.36	4.29	5.71	7.47	9.45	11.44
29	114	0.22	1.57	3.48	5.94	8.77	4.65	6.14	7.98	10.06	12.15
30	179	0.38	1.77	3.74	6.27	9.18	5.01	6.56	8.49	10.67	12.86
31	349	0.54	1.97	3.99	6.61	9.60	5.37	6.99	9.00	11.28	13.58
32	308	0.70	2.17	4.25	6.94	10.02	5.73	7.42	9.52	11.89	14.30
33	231	0.86	2.37	4.52	7.28	10.45	6.09	7.84	10.03	12.51	15.02
34	172	1.02	2.58	4.78	7.62	10.88	6.45	8.27	10.55	13.12	15.74
35	146	1.18	2.78	5.04	7.95	11.30	6.82	8.70	11.06	13.74	16.47
36	226	1.34	2.97	5.29	8.28	11.70	7.18	9.14	11.58	14.36	17.20
37	393	1.49	3.16	5.53	8.59	12.10	7.55	9.57	12.11	14.99	17.93
38	301	1.63	3.34	5.76	8.89	12.47	7.92	10.01	12.63	15.62	18.67
39	120	1.77	3.52	5.99	9.18	12.84	8.30	10.45	13.16	16.25	19.41
40	37	1.91	3.69	6.22	9.48	13.22	8.67	10.90	13.69	16.89	20.16

Table D9 – Study-specific percentiles of gestational weight gain for AMANHI-Pakistan vs INTERGROWTH-21st standards, Normal BMI (Kg)

Gestational age, weeks	Percentiles based on study reference sub-population (Kg)						Percentiles based on INTERGROWTH-21st standards (Kg)				
	Observations, n	p10	p25	p50	p75	p90	p10	p25	p50	p75	p90
19	1	-0.89	0.26	1.90	4.06	6.58	0.82	1.69	2.75	3.92	5.08
21	1	-0.64	0.58	2.32	4.62	7.29	1.65	2.64	3.84	5.17	6.49
22	1	-0.50	0.74	2.54	4.91	7.66	2.05	3.10	4.37	5.79	7.20
23	5	-0.37	0.92	2.77	5.21	8.04	2.44	3.54	4.90	6.41	7.91
24	297	-0.23	1.10	3.01	5.51	8.43	2.82	3.99	5.42	7.02	8.61
25	380	-0.09	1.28	3.24	5.82	8.83	3.19	4.42	5.94	7.63	9.32
26	52	0.05	1.45	3.48	6.13	9.22	3.56	4.85	6.45	8.24	10.02
27	20	0.19	1.63	3.70	6.43	9.60	3.92	5.28	6.96	8.84	10.73
28	17	0.33	1.80	3.93	6.73	9.99	4.29	5.71	7.47	9.45	11.44
29	10	0.47	1.99	4.19	7.06	10.41	4.65	6.14	7.98	10.06	12.15
30	6	0.64	2.20	4.46	7.42	10.86	5.01	6.56	8.49	10.67	12.86
31	8	0.81	2.42	4.75	7.80	11.35	5.37	6.99	9.00	11.28	13.58
32	317	0.99	2.66	5.06	8.21	11.87	5.73	7.42	9.52	11.89	14.30
33	410	1.19	2.91	5.39	8.65	12.44	6.09	7.84	10.03	12.51	15.02
34	60	1.40	3.18	5.74	9.10	13.01	6.45	8.27	10.55	13.12	15.74
35	20	1.60	3.43	6.07	9.54	13.58	6.82	8.70	11.06	13.74	16.47
36	5	1.80	3.68	6.41	9.98	14.14	7.18	9.14	11.58	14.36	17.20
37	8	1.99	3.93	6.73	10.40	14.68	7.55	9.57	12.11	14.99	17.93
38	279	2.18	4.17	7.04	10.81	15.20	7.92	10.01	12.63	15.62	18.67
39	236	2.35	4.40	7.34	11.20	15.70	8.30	10.45	13.16	16.25	19.41
40	12	2.53	4.63	7.64	11.60	16.21	8.67	10.90	13.69	16.89	20.16

Table D10 – Study-specific percentiles of gestational weight gain for PreSSMat vs INTERGROWTH-21st standards, Normal BMI (Kg)

Gestational age, weeks	Percentiles based on study reference sub-population (Kg)						Percentiles based on INTERGROWTH-21st standards (Kg)				
	Observations, n	p10	p25	p50	p75	p90	p10	p25	p50	p75	p90
19	1	-0.44	0.36	1.43	2.71	4.07	0.82	1.69	2.75	3.92	5.08
21	95	0.06	0.96	2.14	3.56	5.08	1.65	2.64	3.84	5.17	6.49
22	774	0.33	1.28	2.53	4.03	5.63	2.05	3.10	4.37	5.79	7.20
23	626	0.61	1.60	2.92	4.49	6.18	2.44	3.54	4.90	6.41	7.91
24	274	0.88	1.92	3.30	4.95	6.72	2.82	3.99	5.42	7.02	8.61
25	107	1.14	2.23	3.67	5.40	7.24	3.19	4.42	5.94	7.63	9.32
26	61	1.40	2.53	4.03	5.83	7.75	3.56	4.85	6.45	8.24	10.02
27	363	1.64	2.82	4.37	6.24	8.23	3.92	5.28	6.96	8.84	10.73
28	536	1.87	3.09	4.70	6.63	8.69	4.29	5.71	7.47	9.45	11.44
29	239	2.11	3.37	5.03	7.03	9.16	4.65	6.14	7.98	10.06	12.15
30	103	2.35	3.65	5.38	7.44	9.64	5.01	6.56	8.49	10.67	12.86
31	480	2.61	3.95	5.74	7.87	10.15	5.37	6.99	9.00	11.28	13.58
32	771	2.87	4.27	6.11	8.32	10.68	5.73	7.42	9.52	11.89	14.30
33	409	3.15	4.59	6.50	8.79	11.23	6.09	7.84	10.03	12.51	15.02
34	101	3.41	4.90	6.87	9.23	11.75	6.45	8.27	10.55	13.12	15.74
35	315	3.64	5.17	7.20	9.63	12.22	6.82	8.70	11.06	13.74	16.47
36	584	3.85	5.42	7.49	9.98	12.63	7.18	9.14	11.58	14.36	17.20
37	365	4.03	5.63	7.75	10.28	12.99	7.55	9.57	12.11	14.99	17.93
38	171	4.18	5.80	7.95	10.53	13.28	7.92	10.01	12.63	15.62	18.67
39	252	4.29	5.94	8.12	10.73	13.51	8.30	10.45	13.16	16.25	19.41
40	217	4.38	6.04	8.24	10.87	13.68	8.67	10.90	13.69	16.89	20.16

Table D11 – Study-specific percentiles of gestational weight gain for GARBH-Ini vs INTERGROWTH-21st standards, Normal BMI (Kg)

Gestational age, weeks	Percentiles based on study reference sub-population (Kg)						Percentiles based on INTERGROWTH-21st standards (Kg)				
	Observations, n	p10	p25	p50	p75	p90	p10	p25	p50	p75	p90
18	1086	-0.93	-0.16	0.86	2.09	3.43	0.37	1.19	2.19	3.28	4.37
19	1168	-0.72	0.08	1.16	2.46	3.86	0.82	1.69	2.75	3.92	5.08
20	213	-0.50	0.34	1.47	2.83	4.30	1.24	2.17	3.30	4.55	5.78
21	20	-0.28	0.61	1.79	3.22	4.77	1.65	2.64	3.84	5.17	6.49
26	1624	0.97	2.09	3.59	5.40	7.36	3.56	4.85	6.45	8.24	10.02
27	674	1.24	2.42	3.98	5.88	7.92	3.92	5.28	6.96	8.84	10.73
28	162	1.51	2.73	4.36	6.33	8.46	4.29	5.71	7.47	9.45	11.44
29	54	1.76	3.03	4.72	6.77	8.99	4.65	6.14	7.98	10.06	12.15
30	238	2.00	3.31	5.06	7.19	9.48	5.01	6.56	8.49	10.67	12.86
31	2005	2.22	3.57	5.38	7.58	9.94	5.37	6.99	9.00	11.28	13.58
32	243	2.43	3.83	5.70	7.95	10.39	5.73	7.42	9.52	11.89	14.30
33	50	2.65	4.09	6.01	8.33	10.84	6.09	7.84	10.03	12.51	15.02
35	465	3.09	4.61	6.63	9.09	11.74	6.82	8.70	11.06	13.74	16.47
36	265	3.30	4.86	6.94	9.47	12.18	7.18	9.14	11.58	14.36	17.20
37	124	3.52	5.12	7.25	9.84	12.63	7.55	9.57	12.11	14.99	17.93
38	325	3.73	5.37	7.56	10.21	13.07	7.92	10.01	12.63	15.62	18.67
39	532	3.95	5.63	7.87	10.59	13.51	8.30	10.45	13.16	16.25	19.41
40	603	4.16	5.88	8.17	10.95	13.95	8.67	10.90	13.69	16.89	20.16

Table D12 – Study-specific percentiles of gestational weight gain for ZAPPS vs INTERGROWTH-21st standards, Normal BMI (Kg)

Gestational age, weeks	Percentiles based on study reference sub-population (Kg)						Percentiles based on INTERGROWTH-21st standards (Kg)				
	Observations, n	p10	p25	p50	p75	p90	p10	p25	p50	p75	p90
19	1	-1.21	-0.13	1.44	3.51	5.93	0.82	1.69	2.75	3.92	5.08
20	1	-1.03	0.10	1.74	3.90	6.44	1.24	2.17	3.30	4.55	5.78
21	30	-0.85	0.33	2.05	4.32	6.97	1.65	2.64	3.84	5.17	6.49
22	224	-0.66	0.58	2.38	4.75	7.52	2.05	3.10	4.37	5.79	7.20
23	140	-0.46	0.84	2.72	5.20	8.10	2.44	3.54	4.90	6.41	7.91
24	133	-0.25	1.11	3.07	5.66	8.70	2.82	3.99	5.42	7.02	8.61
25	81	-0.03	1.38	3.43	6.14	9.32	3.19	4.42	5.94	7.63	9.32
26	24	0.18	1.66	3.80	6.63	9.94	3.56	4.85	6.45	8.24	10.02
27	18	0.40	1.94	4.17	7.12	10.57	3.92	5.28	6.96	8.84	10.73
28	101	0.62	2.22	4.54	7.61	11.20	4.29	5.71	7.47	9.45	11.44
29	49	0.84	2.50	4.91	8.10	11.83	4.65	6.14	7.98	10.06	12.15
30	6	1.06	2.78	5.28	8.59	12.46	5.01	6.56	8.49	10.67	12.86
31	32	1.27	3.06	5.65	9.07	13.08	5.37	6.99	9.00	11.28	13.58
32	239	1.48	3.33	6.01	9.55	13.69	5.73	7.42	9.52	11.89	14.30
33	117	1.70	3.61	6.37	10.03	14.31	6.09	7.84	10.03	12.51	15.02
34	15	1.93	3.91	6.77	10.55	14.98	6.45	8.27	10.55	13.12	15.74
35	31	2.19	4.24	7.21	11.14	15.73	6.82	8.70	11.06	13.74	16.47
36	232	2.48	4.61	7.70	11.78	16.56	7.18	9.14	11.58	14.36	17.20
37	122	2.80	5.02	8.24	12.50	17.48	7.55	9.57	12.11	14.99	17.93
38	10	3.14	5.45	8.81	13.25	18.45	7.92	10.01	12.63	15.62	18.67
39	2	3.49	5.91	9.41	14.05	19.47	8.30	10.45	13.16	16.25	19.41

Table D13. Risk ratios for the association between gestational weight gain and adverse outcomes among normal BMI participants. Weight-gain-for-gestational age Z-scores defined using INTERGROWTH-21st (IG-21) weight gain standards.

		Overall			AMANHI-Bangladesh			AMANHI-Pakistan			PreSSMat[b]			GARBH-Ini			ZAPPS		
	IG-21 GWG group	Risk (n/N)	Adj Risk,[a] % (95% CI)	Adj RR[a], (95% CI)	Risk (n/N)	Adj Risk,[a] % (95% CI)	Adj RR[a], (95% CI)	Risk (n/N)	Adj Risk,[a] % (95% CI)	Adj RR[a], (95% CI)	Risk (n/N)	Adj Risk,[a] % (95% CI)	Adj RR[a], (95% CI)	Risk (n/N)	Adj Risk,[a] % (95% CI)	Adj RR[a], (95% CI)	Risk (n/N)	Adj Risk,[a] % (95% CI)	Adj RR[a], (95% CI)
PTB	1	786/5978	12.5 (11.2, 14.0)	1.04 (0.86, 1.25)	161/1362	11.2 (9.6, 13.0)	1.26 (0.61, 2.60)	84/771	9.7 (7.8, 12.0)	0.61 (0.37, 0.99)	170/1325	11.5 (9.9, 13.5)	1.19 (0.78, 1.81)	332/2180	14.4 (13.0, 16.0)	1.13 (0.82, 1.55)	39/340	9.6 (6.9, 13.4)	0.88 (0.44, 1.74)
	2	172/1365	12.3 (11.4, 13.3)	1.02 (0.87, 1.19)	31/209	14.3 (10.4, 19.8)	1.61 (0.74, 3.55)	17/128	11.3 (7.5, 17.1)	0.71 (0.39, 1.30)	45/413	10.5 (8.0, 13.7)	1.08 (0.68, 1.73)	64/525	12.1 (9.6, 15.1)	0.95 (0.65, 1.38)	15/90	13.8 (8.2, 23.3)	1.26 (0.59, 2.70)
	3	91/731	12.1 (10.4, 14.0)	1	7/78	8.9 (4.3, 18.2)	1	16/90	15.9 (10.1, 25.1)	1	22/222	9.7 (6.5, 14.4)	1	37/271	12.8 (9.4, 17.3)	1	9/70	11.0 (6.0, 20.0)	1
	4	58/309	17.7 (12.9, 24.1)	1.46 (1.10, 1.95)	4/32	12.3 (4.8, 31.2)	1.38 (0.43, 4.47)	15/54	23.5 (15.3, 36.0)	1.48 (0.82, 2.66)	12/62	19.5 (11.7, 32.6)	2.02 (1.07, 3.81)	24/113	20.7 (14.5, 29.7)	1.62 (1.02, 2.59)	3/48	3.9 (1.3, 12.0)	0.36 (0.10, 1.24)
LBW	1	1671/5978	26.6 (24.1, 29.4)	1.61 (1.31, 1.99)	415/1362	28.8 (26.2, 31.7)	1.40 (0.88, 2.23)	200/771	24.6 (21.5, 28.1)	1.30 (0.85, 2.01)	325/1325	22.7 (20.3, 25.3)	2.28 (1.47, 3.54)	677/2180	30.1 (28.2, 32.2)	1.54 (1.19, 1.98)	55/340	13.1 (9.9, 17.4)	1.10 (0.59, 2.05)
	2	251/1365	17.7 (15.1, 20.7)	1.07 (0.91, 1.26)	51/209	23.4 (18.3, 29.9)	1.14 (0.68, 1.91)	24/128	16.9 (11.5, 24.9)	0.90 (0.52, 1.55)	58/413	13.7 (10.5, 17.9)	1.38 (0.84, 2.26)	105/525	19.9 (16.7, 23.8)	1.02 (0.75, 1.37)	12/90	10.4 (6.0, 17.9)	0.87 (0.41, 1.86)
	3	124/731	16.5 (12.6, 21.6)	1	17/78	20.6 (12.9, 32.8)	1	18/90	18.9 (12.5, 28.6)	1	24/222	10.0 (6.5, 15.3)	1	54/271	19.6 (15.3, 25.0)	1	10/70	11.9 (6.7, 21.1)	1
	4	50/309	15.9 (11.2, 22.4)	0.96 (0.70, 1.32)	8/32	25.6 (13.7, 48.1)	1.24 (0.56, 2.77)	14/54	24.6 (15.3, 39.6)	1.30 (0.70, 2.42)	6/62	9.3 (3.9, 22.2)	0.93 (0.36, 2.45)	18/113	15.4 (9.9, 23.9)	0.79 (0.48, 1.30)	4/48	5.2 (1.9, 14.2)	0.44 (0.14, 1.36)
SGA 10	1	2333/5969	37.6 (34.9, 40.5)	1.57 (1.33, 1.86)	600/1362	42.5 (39.7, 45.5)	1.30 (0.93, 1.82)	295/771	37.1 (33.7, 40.9)	1.51 (1.02, 2.24)	447/1324	32.2 (29.5, 35.1)	1.88 (1.35, 2.61)	905/2173	40.4 (38.3, 42.6)	1.40 (1.14, 1.70)	86/339	24.5 (20.1, 29.9)	2.08 (1.07, 4.06)
	2	362/1362	25.6 (22.1, 29.6)	1.07 (0.94, 1.21)	62/209	28.5 (22.9, 35.5)	0.87 (0.59, 1.30)	41/128	29.7 (22.8, 38.8)	1.21 (0.77, 1.92)	89/413	21.1 (17.2, 25.8)	1.23 (0.85, 1.77)	157/522	29.2 (25.5, 33.4)	1.01 (0.80, 1.27)	13/90	13.6 (8.1, 22.8)	1.16 (0.51, 2.61)
	3	178/729	23.9 (18.9, 30.2)	1	27/78	32.8 (23.4, 45.9)	1	23/90	24.5 (16.7, 36.0)	1	40/222	17.2 (12.5, 23.6)	1	79/270	28.9 (23.9, 35.1)	1	8/69	11.8 (6.3, 22.1)	1
	4	70/308	22.7 (17.3, 29.9)	0.95 (0.63, 1.44)	12/32	35.7 (21.8, 58.7)	1.09 (0.59, 2.00)	15/54	26.9 (17.0, 42.3)	1.09 (0.61, 1.95)	15/62	24.9 (15.6, 39.8)	1.45 (0.82, 2.57)	20/112	17.2 (11.3, 26.2)	0.60 (0.38, 0.94)	7/48	13.8 (6.7, 28.6)	1.18 (0.45, 3.06)
SGA 3	1	1110/5969	17.2 (15.0, 19.7)	1.92 (1.32, 2.78)	323/1362	22.2 (19.7, 24.9)	2.29 (1.06, 4.95)	143/771	16.9 (14.2, 20.1)	2.20 (1.04, 4.67)	185/1324	12.7 (10.8, 14.9)	2.84 (1.44, 5.62)	427/2173	18.6 (16.9, 20.4)	1.40 (1.02, 1.92)	32/339	6.2 (3.9, 9.8)	1.31 (0.50, 3.43)
	2	156/1362	10.5 (8.3, 13.4)	1.18 (0.90, 1.54)	27/209	12.2 (8.4, 17.8)	1.26 (0.54, 2.96)	17/128	11.2 (6.9, 18.3)	1.46 (0.62, 3.46)	34/413	7.4 (5.1, 10.8)	1.66 (0.78, 3.55)	73/522	13.2 (10.6, 16.6)	1.00 (0.69, 1.46)	5/90	3.8 (1.5, 9.7)	0.80 (0.23, 2.83)
	3	70/729	9.0 (5.7, 14.0)	1	8/78	9.7 (4.4, 21.1)	1	8/90	7.7 (3.7, 16.0)	1	12/222	4.5 (2.3, 8.7)	1	38/270	13.2 (9.7, 18.0)	1	4/69	4.8 (2.0, 11.5)	1
	4	27/308	8.3 (6.2, 11.2)	0.93 (0.53, 1.64)	4/32	10.9 (3.9, 30.7)	1.13 (0.31, 4.09)	7/54	12.1 (5.9, 24.7)	1.58 (0.58, 4.33)	5/62	6.9 (2.7, 17.3)	1.55 (0.50, 4.84)	9/112	7.4 (3.9, 14.1)	0.56 (0.27, 1.14)	2/48	3.1 (0.7, 13.6)	0.66 (0.12, 3.53)

PTB, preterm birth; LBW, low birthweight (<2500g), SGA10 (small-for-gestational age <10th percentile), SGA3 (small-for-gestational-age <3rd percentile)

IG-21 GWG group z-score ranges (percentile). 1: ≤ -0.67 (≤ 25th percentile); 2: -0.68 - 0.00 (26 - 50th percentile); 3: 0.01 - 0.67 (51 - 75th percentile); 4: > 0.067 (> 75th percentile)

[a] Adjusted for maternal age, gestational age at enrollment, maternal height, maternal BMI, parous, previous preterm birth

[b] For GAPPS-B only: reporting results from complete-case analysis for PTB among the underweight, overweight, and obese because those participants did not have any missing covariate data
